# The use of pledget-reinforced sutures during surgical aortic valve replacement: A systematic review and *meta*-analysis

**DOI:** 10.1016/j.ijcha.2024.101494

**Published:** 2024-08-22

**Authors:** J.W. Taco Boltje, Mathijs T. Carvalho Mota, Michiel D. Vriesendorp, Alexander B.A. Vonk, Rolf H.H. Groenwold, Robert J.M. Klautz, Bart J.J. Velders

**Affiliations:** a Department of Cardiothoracic Surgery, Amsterdam University Medical Center Location AMC, Amsterdam, the Netherlands; b Department of Cardiothoracic Surgery, Leiden University Medical Center, Leiden, the Netherlands; c Department of Clinical Epidemiology, Leiden University Medical Center, Leiden, the Netherlands; d Department of Biomedical Data Science, Leiden University Medical Center, Leiden, the Netherlands

**Keywords:** Surgical aortic valve replacement, Sutures, Pledgets, Paravalvular leakage, Mortality, Mean-pressure gradient, Effective orifice area

## Abstract

**Objective:**

Literature presents conflicting results on the pros and cons of pledget-reinforced sutures during surgical aortic valve replacement (SAVR). We aimed to investigate the effect of pledget-reinforced sutures versus sutures without pledgets during SAVR on different outcomes in a systematic review and *meta*-analysis.

**Methods:**

A literature search was performed in five different medical literature databases. Studies must include patients undergoing SAVR and must compare any pledget-reinforced with any suturing technique without pledgets. The primary outcome was paravalvular leakage (PVL), and secondary outcomes comprised thromboembolism, endocarditis, mortality, mean pressure gradient (MPG) and effective orifice area (EOA). Results were pooled using a random-effects model as risk ratios (RRs) or mean differences (MDs) for which the no pledgets group served as reference.

**Results:**

Nine observational studies met the inclusion criteria. The risk of bias was critical in seven studies, and high and moderate in two other. The pooled RR for moderate or greater PVL was 0.59 (95 % confidence interval [CI] 0.13, 2.73). The pooled RR for mortality at 30-days was 1.02 (95 % CI 0.48, 2.18) and during follow-up was 1.15 (95 % CI 0.67, 2.00). For MPG and EOA at 1-year follow-up, the pooled MDs were 0.60 mmHg (95 % CI −4.92, 6.11) and −0.03 cm^2^ (95 % CI −0.18, 0.12), respectively.

**Conclusions:**

Literature on the use of pledget-reinforced sutures during SAVR is at high risk of bias. Pooled results are inconclusive regarding superiority of either pledget-reinforced sutures or sutures without pledgets. Hence, there is no evidence to support or oppose the use of pledget-reinforced sutures.

## Graphical abstract

The figure demonstrating the suturing techniques is reproduced and adapted from Saisho *et al.*[24] with permission from Oxford University Press.

**Figure:**
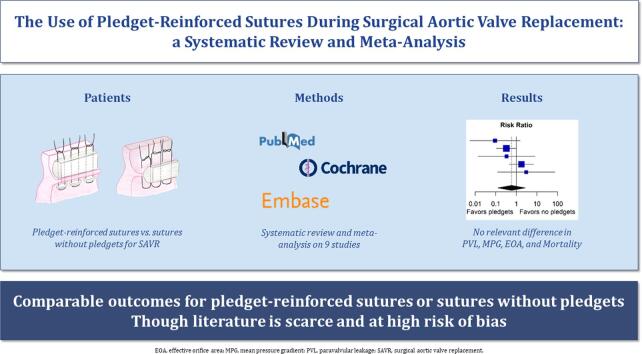

## 1 Introduction

More than 60 years ago the first successful surgical aortic valve replacement (SAVR) took place [1]. Since then, numerous advancements have been made to improve the outcomes of individual patients. However, there is still no consensus on some aspects of this surgical procedure including whether pledget-reinforced sutures should be used to implant the prosthetic valve. Experience learns that, even within one center, it strongly depends on the training and preference of the surgeon which suturing technique is applied.

Previous studies have reported conflicting results for outcomes that could be affected by pledgets [2], [3], [4], [5], [6], [7]. For example, Englberger *et al.* [2] reported lower incidences of paravalvular leak (PVL) when pledgets were used while other studies found similar incidences for suturing techniques with and without pledgets [3], [4], [5], [6], [7]. To explore the quality of the available literature and examine pooled effects, a systematic review and *meta*-analysis was performed. Specifically, this study aimed to investigate the effect of any pledget-reinforced suturing technique, as compared to any suturing technique without pledgets, during SAVR on different hemodynamic and clinical outcomes. The goal of this *meta*-analysis is to provide a clinical recommendation for or against the use of pledgets during SAVR.

## 2 Methods

For this *meta*-analysis, the Preferred Reporting Items for Systematic Reviews and Meta-Analyses (PRISMA) guidelines were followed [8] and for the development of the protocol, the PRISMA guidelines for protocols (PRISMA-P) [9]. The protocol was preregistered prior to the start of the study on PROSPERO with ID number 433066 (CRD42023433066). The primary outcome was moderate or greater PVL post-implantation up to 30 days. Secondary outcomes included thromboembolism, endocarditis, mortality, mean pressure gradient (MPG), and effective orifice area (EOA). All outcomes were measured post-implantation up to 30 days and during mid-term follow-up.

### 2.1 Study selection, data extraction and risk of bias assessment

A literature search was performed in PubMed, Cumulative Index to Nursing and Allied Health Literature (CINAHL), Cochrane, Embase and Web of Science on June the 7th 2023. Together with a librarian a search string was developed, which is included in the supplementary files. The main components were based on the population, patients undergoing SAVR, and the intervention, pledget-reinforced sutures. Studies were selected according to the following eligibility criteria: studies must include patients undergoing SAVR, with or without concomitant cardiac surgery, and must compare any pledget-reinforced suturing technique with any suturing technique without pledgets. Observational studies and randomized controlled trials published in peer-reviewed journals were included. Systematic reviews, *meta*-analyses, and conference abstracts were excluded as well as studies in any language other than English. Two researchers (TB & MC) independently performed title/abstract and full-text screening, using Rayyan software [10], as well as data extraction and risk of bias assessment on study level. Any disagreement was discussed with a third researcher (BV). Data extraction was performed using a prespecified form based on the Cochrane format. If studies included more than one treatment arm with pledget-reinforced sutures or sutures without pledgets, these were grouped to one arm with and without pledgets. If this was not possible, the largest group with pledget-reinforced sutures and the largest group with sutures without pledgets were contrasted. The Risk Of Bias In Non-randomized Studies of Interventions (ROBINS-I) was use [11].

### 2.2 Statistical analysis

For dichotomous outcomes, risk ratios (RRs) including 95 % confidence interval (CI) were extracted or calculated using the cumulative incidences per treatment group. If multiple results on the same outcome were reported, e.g., unadjusted and adjusted for potential confounders, the risk ratio after confounding adjustment was preferred. For continuous outcomes like MPG and EOA, mean differences (MDs) were pooled. Results were pooled using a Hartung-Knapp random-effects model [12] and results were presented using forest plots. The Hartung-Knapp model was preferred because this model provides a realistic estimation of the uncertainty in treatment effect when only limited studies are available [12], [13]. To assess heterogeneity, the I^2^ was estimated and a 95 % prediction interval was calculated around the pooled estimate [14]. This prediction interval depicts the expected range of the true treatment effect in a new study [15]. Furthermore, the potential of publication bias was evaluated using Egger’s test [16] and visualized in funnel plots. The Grading of Recommendations, Assessment, Development, and Evaluations (GRADE) framework was used for making clinical practice recommendations about the use of pledget-reinforced sutures during SAVR [17]. All statistical analyses were executed using the statistical software R (R Foundation for Statistical Computing, Vienna, Austria, www.r-project.org), specifically the R packages *meta* and *robvis*. The data extraction forms, risk of bias assessments, final study data and R script are all available in the supplementary files.

## 3 Results

### 3.1 Systematic review

**Fig. 1:**
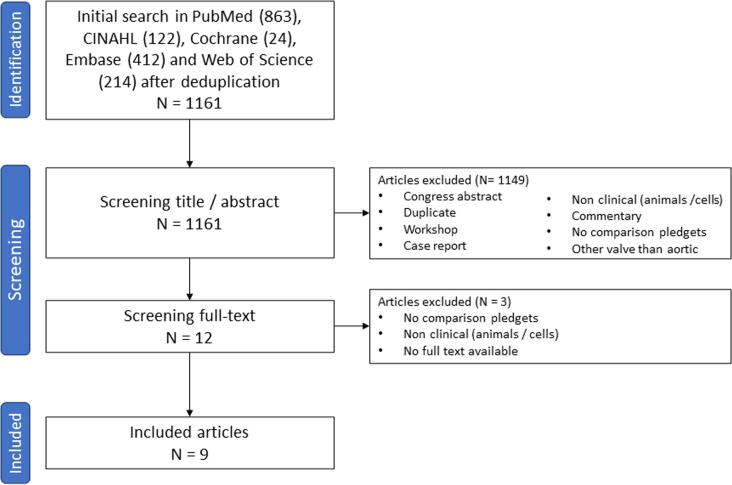
**Flowchart of the selection process of studies on the use of pledget-reinforced sutures during surgical aortic valve replacement.** A schematic presentation of the literature review executed according to the PRISMA guidelines. PRISMA, Preferred Reporting Items for Systematic Reviews and Meta-Analyses.

**Fig. 2:**
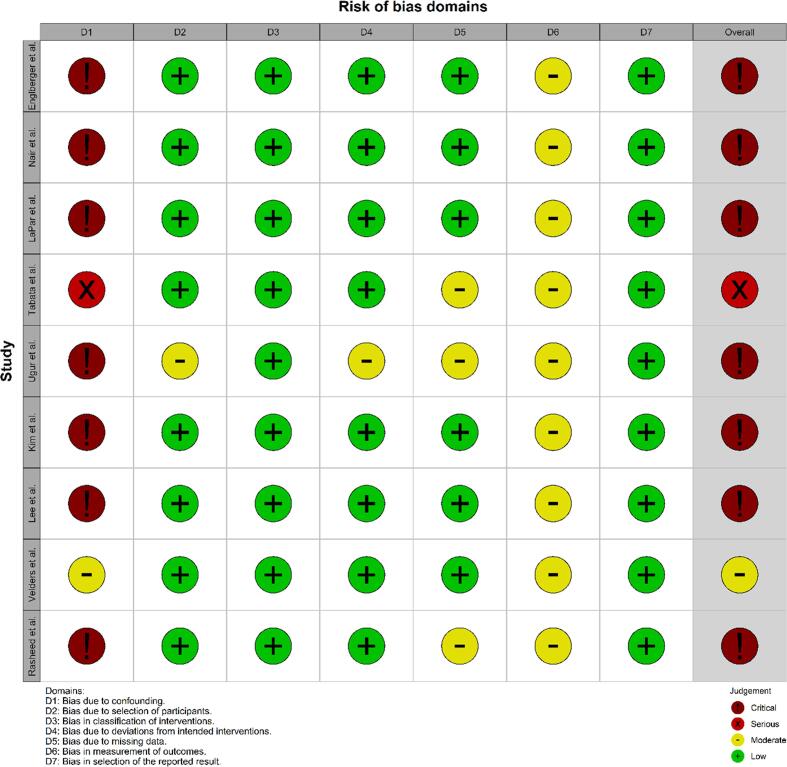
**Risk of bias assessment for studies on the use of pledget-reinforced sutures during surgical aortic valve replacement.** Risk of bias assessment displayed per article according to ROBINS-I. ROBINS-I, Risk Of Bias In Non-randomized Studies of Interventions.

**Fig. 3:**
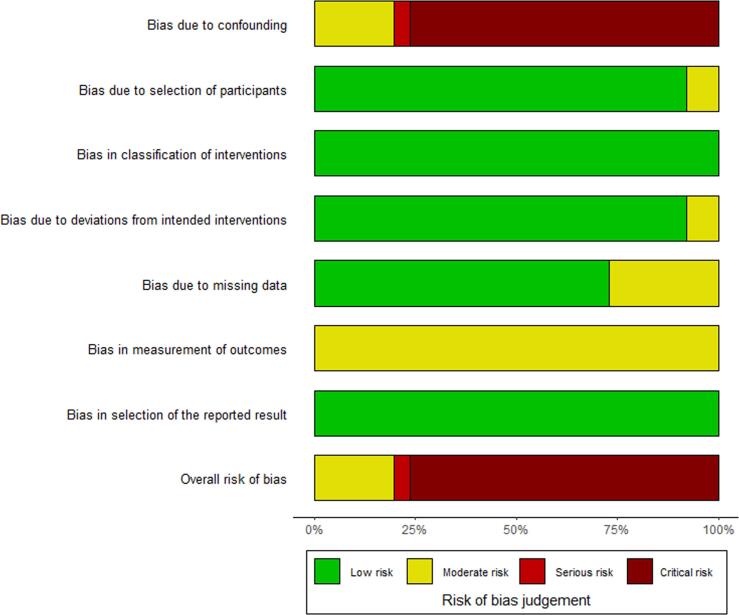
**Risk of bias assessment per domain for the included articles on the use of pledget-reinforced sutures during surgical aortic valve replacement.** Risk of bias assessment displayed per domain according to ROBINS-I. ROBINS-I, Risk Of Bias In Non-randomized Studies of Interventions.

The literature search provided a total of 1161 unique studies. After title and abstract screening, 12 articles were selected for full-text reading. Three studies were excluded because these lacked a comparison of pledget-reinforced sutures and sutures without pledgets, no human subjects were involved or no full-text was available [18], [19], [20]. Nine studies were eligible for analysis [2], [3], [4], [5], [6], [7], [21], [22], [23]. Fig. 1 illustrates the selection process through a flowchart. The selected studies were all observational studies, of which four with prospective and five with retrospective data collection. Two studies were a secondary analysis of an RCT, however, the patients in this study were not randomized to pledget-reinforced sutures but to two types of prosthetic valves [2], [21]. An overview of the study characteristics, patient characteristics, and clinical outcomes is provided in Table 1 and of the risk of bias assessment in Fig. 2, Fig. 3.

**Table 1:** Overview of studies on the use of pledget-reinforced sutures during surgical aortic valve replacement.

Study characteristics									Patient characteristics		Clinical outcomes
Reference	**Year**	**Design**	**N**	**Pledgets / no pledgets**	**Suturing technique, Pledgets / no pledgets**	FU*	**Primary indication**	**Valve type / size**	**Age (y)**	**Male (%)**	**Endpoints**
Englberger *et al.* (2)	2005	Secondary analysis of RCT	549^a^	414 / 135	P/NP: Simple interrupted, continuous, non-everted and everted mattress, and figure-of-eight	30.6	Stenosis (46.7 %), Insufficiency (22.3 %), Mixed (30.7 %)	Mechanical; all sizes	61.3	58.9	PVL
Nair *et al.* (21)	2010	Secondary analysis of RCT	126	43 / 83	P: interrupted buttressed sutures.NP: Semi-continuous	120	Stenosis (72.2 %), Insufficiency (17.8 %)	Mechanical; all sizes	62.7	79.4	PVL
LaPar *et al.* (3)	2011	Retrospective cohort	802	291 / 511	P: horizontal mattress.NP: horizontal mattress	82	Stenosis (81.8 %), Insufficiency (31.6 %),	Mechanical and biological; all sizes	65.2	59.6	PVL, mortality and thromboembolism
Tabata *et al.* (4)	2014	Retrospective cohort	152	102 / 50	P: Non-everting mattressNP: simple interrupted	12	Stenosis (92.1 %), Insufficiency unknown	Biological; 19 or 21	76.6	26.3	PVL, EOA
Ugur *et al.* (5)	2014	Prospective cohort	346	269 / 77	P: Non-everting mattressNP: Everting mattrass, simple interrupted or continuous	12	Unknown	Biological; 19 or 21	75.5	29.5	PVL, MPG, EOA
Kim *et al.* (6)	2020	Retrospective cohort	439	212 / 227	P: Interrupted mattress NP: interrupted mattress or figure-of-eight	16	Stenosis (96.8 %)^b^, Insufficiency (15.5 %)^b^	Mechanical and biological; all sizes, sub-analysis < 21	64.2	57.4	PVL, mortality, EOA
Lee *et al.* (22)	2020	Retrospective cohort	215	136 / 79	P: Non-everting mattressNP: simple interrupted	9.6	Stenosis (100 %)^c^	Mechanical and biological; all sizes, sub-analysis < 23	66.9	62.3	PVL, MPG, EOA
Velders *et al.* (7)	2023	Prospective cohort	1082	640 / 442	P: Non-everting or everting mattressNP: Non-everted or everted mattress, and simple interrupted	60	Isolated or mixed stenosis (94.5 %)	Biological; all sizes, sub-analysis < 23	70.2	75.5	PVL, mortality, endocarditis,thromboembolism, MPG, EOA
Rasheed *et al.* (23)	2023	Retrospective cohort	629	570 / 59	P: horizontal mattressNP: figure-of-eight	NA	Stenosis (62.2 %), Insufficiency (43.4 %)^d^	Mechanical and biological; all sizes, sub-analysis < 23	64.2	35.2	Predicted EOA index

a. Only aortic valve replacements of the 807 aortic and mitral valve replacements.

b. Only severe aortic valve stenosis or regurgitation.

c. Patients were only included with severe aortic stenosis.

d. Moderate to severe aortic valve regurgitation

*Follow-up length is expressed in months. EOA, effective orifice area; FU, follow-up; MPG, mean pressure gradient; NA, not available; NP, non-pledget suturing techniques; P, pledget suturing techniques; PVL, paravalvular leak; RCT, randomized controlled trial.

Englberger *et al.* [2] performed a retrospective analysis of their AVERT RCT data which included both mechanical SAVR and mitral valve replacement. The analysis comprised a total of 549 aortic valve patients. Englberger *et al.* reported PVL, had a mean follow-up of 30.6 months and risk of bias was critical mainly because no adjustment for confounding was made.

Nair *et al.* [21] retrospectively analyzed data from another RCT. 126 patients received a mechanical aortic valve prosthesis. The primary endpoint was PVL and the risk of bias was critical due to the risk of bias in confounding.

LaPar *et al.* [3] executed a retrospective cohort study with 802 patients, which included both mechanical and biological SAVRs. The mean follow-up was 82.0 months. Outcome measures included PVL, mortality and thromboembolism and the risk of bias was critical mainly because no adjustment for confounding was made.

Tabata *et al.* [4] included 152 SAVR patients who received a 19- or 21-mm biological valve. In this retrospective cohort study, PVL and EOA were reported up to one year post-SAVR and risk of bias was serious since multivariable outcome regression was used for a few confounding factors namely sex, body surface area, ejection fraction, annulus size and implantation of 19-mm prosthesis.

Ugur *et al.* [5] included 346 SAVR patients who were implanted with a 19- or 21 mm bioprosthesis. In this prospective cohort study, the mean follow-up was 12 months at which PVL, MPG and EOA were measured. Due to a lack of adjustment methods for confounding, risk of bias was judged as critical.

Kim *et al.* [6] performed a retrospective cohort study including 439 mechanical or biological SAVR patients. The mean follow-up was 16 months. PVL, mortality and EOA were reported and the risk of bias was critical mainly because the study lacked adjustment methods for confounding.

Lee *et al.* [6] included 215 mechanical or biological SAVR patients in a retrospective cohort study. MPG, EAO and PVL were reported up to a median follow-up of 9.6 months and PVL up to 26 months post-operatively. Mainly because no adjustment method for confounding was used, the risk of bias was judged as critical.

Velders *et al.* [7] performed a prospective cohort study which included 1082 biological SAVR patients. The authors reported on PVL, mortality, endocarditis, thromboembolism, MPG and EOA up to 60 months post-operatively. Propensity score matching was used based on multiple confounding variables and the risk of bias was judged as moderate.

Rasheed *et al.* [23] included 629 mechanical or biological SAVR patients in a retrospective cohort study. The predicted EOA index was reported as the outcome. Risk of bias was critical mainly because no adjustment method for confounding was used.

### 3.2 *Meta*-analysis

Outcomes were pooled if reported by at least three individual studies. An overview of the reported outcomes (including the time of outcome measurement) per study is provided in Supplementary Tables S1 andTable S2. In this section the results reported are the outcomes from the random effects model.

**Fig. 4:**
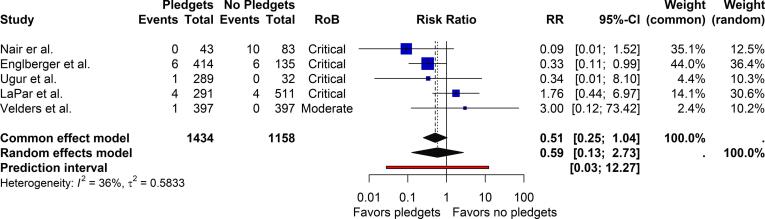
**Forest plot on moderate or greater paravalvular leak at mid-term follow-up after surgical aortic valve replacement.** CI, confidence interval; RoB, risk of bias; RR, risk ratio.

**Fig. 5:**
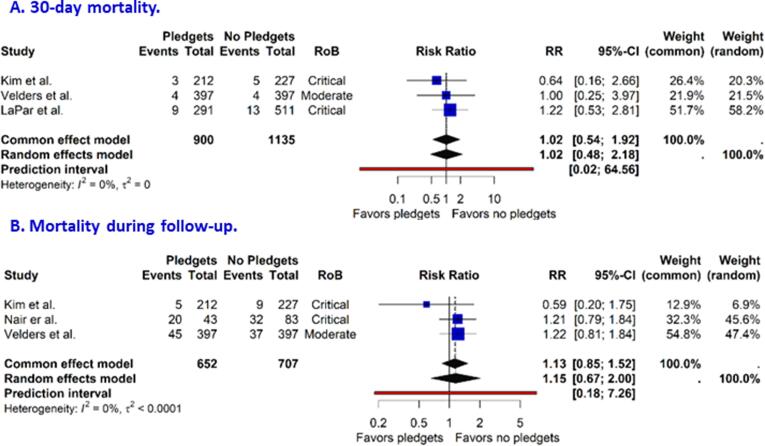
**Forest plot on 30-day mortality after surgical aortic valve replacement.** CI, confidence interval; RoB, risk of bias; RR, risk ratio.

**Fig. 6:**
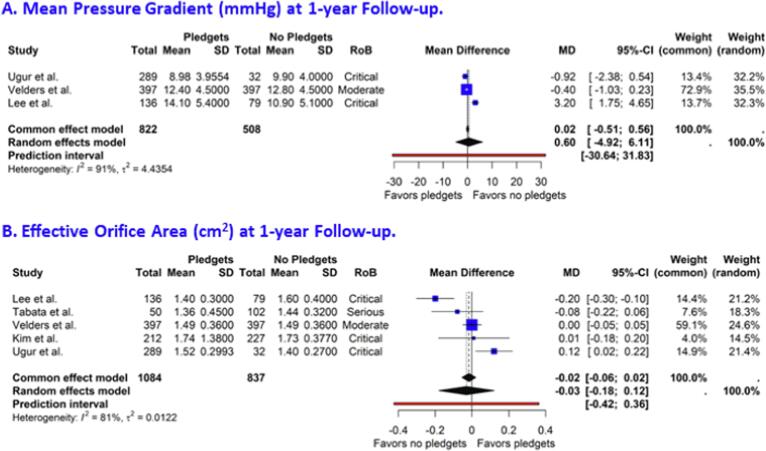
**Forest plot on mean pressure gradient and effective orifice area at 1-year follow-up after surgical aortic valve replacement.** CI, confidence interval; SD, standard deviation; MD, mean difference; RoB, risk of bias.

Moderate or greater PVL post-implantation was reported by five studies at mid-term follow-up. The risk ratio (RR) for pledget-reinforced sutures versus sutures without pledgets was 0.59 (95 % CI 0.13, 2.73, Fig. 4). The 95 % prediction interval ranged from 0.01 to 46.20. Three studies reported on 30-day mortality. The pooled RR was 1.02 (95 % CI 0.48, 2.18, Fig. 5a). The pooled RR for mortality during follow-up, reported by three studies, was 1.15 (95% CI, 0.67, 2.00, Fig. 5b). The MPG and the EOA at 1-year follow-up were reported by three and five studies, respectively. The pooled MDs were 0.60 mmHg (95 % CI -4.92, 6.11, Fig. 6a) for MPG and −0.03 cm^2^ (95 % CI −0.18, 0.12, Fig. 6b) for EOA, both numerically in favor of sutures without pledgets. The 95 % prediction intervals for the MD in MPG and EOA were large: −30.64 to 31.83 mmHg and −0.42 to 0.36 cm^2^, respectively. For the outcomes reported above, funnel plots are presented in Figure S1. These indicated a low suspicion on publication bias which was also reiterated by high p-values for the Egger’s test: 0.98 for PVL, 0.36 for mortality, 0.64 for MPG and 0.77 for EOA, respectively.

For outcomes which were reported by less than three studies the results are summarized in the supplementary files (Figure S2 - S3). The risks on moderate or greater PVL, mortality, thromboembolism and infective endocarditis at 30-days and during mid-term follow-up were low in both the pledget-reinforced sutures and sutures without pledgets group.

## 4 Discussion

This systematic review and *meta*-analysis provides an overview of the available studies that compared pledget-reinforced sutures to sutures without pledgets for SAVR. Literature on this topic is scarce and at high risk of bias. The pooled results do not demonstrate superiority for any of the two techniques for valve-related outcomes including PVL, mortality, MPG, and EOA.

Numerically, the results for PVL were slightly in favor of pledget-reinforced sutures, while the pooled results for MPG and EOA favored suturing techniques without pledgets. However, the wide confidence and prediction intervals indicate large uncertainty because of the limited amount of included studies and the low number of clinical events. Furthermore, the pooled differences for MPG and EOA were very small (0.60 mmHg and −0.04 cm^2^) and therefore unlikely to be clinically relevant. To note, the pooled estimates for MPG and EOA represent the difference at 1-year post-implantation and these could become larger with longer follow-up. For example, Velders *et al.* [7] reported that the EOA in the pledget-reinforced suture group was about 0.10 cm^2^ smaller 5 years after SAVR. This requires further confirmation in future studies. Besides using the Hartung Knapp method for analysis the common effect model was also used. This method, just as the random effects model, has no significant outcomes and the RR or MD differed only minimally from the Hartung Knapp method.

Patients for which the choice between pledget-reinforced sutures and sutures without pledgets could be extra important are the ones with a small aortic annulus. Several included studies have separately reported their outcomes for labelled valve sizes smaller than 21-mm or 23-mm [6], [7], [22], [23] or have specifically selected 19- or 21-mm valves [4], [5]. In these analyses, the EOA and MPG were slightly in favor of sutures without pledgets [4], [6], [22], [23], except for one subgroup analysis in which comparable results were found [7]. Again, differences were small and unfortunately the reported information was too limited to present in a subanalysis. Current literature is insufficient to draw any firm conclusions for patients with a small aortic annulus and more studies are needed.

The focus of this review was on the difference between any pledget-reinforced suturing technique versus any technique without pledgets. However, there are multiple suturing techniques which can be used for prosthetic valve implantation. Saisho *et al.* tested different suture techniques in an ex vivo study; non-everting mattress sutures with pledgets, and single interrupted, continuous and figure-of-eight sutures without pledgets [24]. Figure-of-eight sutures provided the largest EOAs. Two clinical studies included in this review specifically analyzed this suturing technique as a separate treatment group but did not find larger EOAs [6], [23]. No further differentiating between suturing techniques was done because of the already small number of patients per study arm. This and the fact that six of the nine included studies did not report their results separately per suturing technique made it hard to provide conclusive and reliable answers [22], [3], [4], [5], [6], [7]. Specifically for pledget-reinforced sutures, two studies have reported to have used both everting and non-everting sutures where the pledget is placed at the aortic and ventricular side respectively [2], [7]. The limited amount of available data on these differences made analysis on this subject very unreliable and was therefore not peformed. Furthermore, Kim *et al.* reported longer cardiopulmonary bypass and aortic cross-clamp times when these techniques were used [6]. Further research on the optimal suturing technique for SAVR is of interest to optimize hemodynamic performance and to facilitate the best lifetime management.

With regard to the latter, the suturing technique during the primary SAVR procedure might influence the feasibility and outcomes of future interventions. Redo surgery might be harder when pledget-reinforced sutures have initially been used. For future valve-in-valve procedures, it is essential to create the best possible set-up during the index SAVR. If specific suturing techniques could improve the EOA of the initial surgical valve, these would be preferred to optimize the outcomes of subsequent transcatheter reinterventions.

### 4.1 GRADE recommendations

According to the GRADE framework, the evidence summarized in our *meta*-analysis is considered to have a low level of certainty [17]. The magnitude of the observed effects was small, insignificant and imprecise. In addition, the included studies were at high risk of bias. There is currently no scientific argument to plead for or against the use of pledgets. These findings suggests that surgeons can stick to their preferred suturing technique until more conclusive evidence is available.

### 4.2 Limitations

The limitations of this systematic review and *meta*-analysis comprise the small number of available studies and their generally low methodological quality. Most included studies reported on few endpoints at varying follow-up times. We have chosen not to include literature written in any other language than English, which might have influenced the amount of available literature. Furthermore, the prevalence of these endpoints was also low in this *meta*-analysis. Moreover, in the included observational studies, the impact of the surgeon on outcomes could be an inextricable source of confounding. If experienced surgeons favor a particular suturing technique, the comparison between pledgets and no pledgets would be intertwined with a comparison in surgical experience. The condition of the native annulus could also have affected the decision to use pledgets and the outcomes after SAVR. However, most studies did not provide any information regarding this aspect. In addition, in some of the included studies older generation surgical valves were used which might rarely be used in contemporary practice. Lastly, limited additional details for subgroups like patients with a small annulus or for specific suturing techniques could be provided. On the contrary, this systematic review and *meta*-analysis generated a comprehensive overview of all available evidence on the use of pledget-reinforced sutures during SAVR. The analysis was executed conform a preregistered protocol and full access is provided to the study data and the statistical code.

## 5 Conclusions

For the choice between pledget-reinforced sutures or sutures without pledgets during SAVR, literature is scarce and at high risk of bias. Pooled results are inconclusive regarding superiority of either pledget-reinforced sutures or sutures without pledgets. There is no evidence to support or oppose the use of pledget-reinforced sutures.

## Funding statement

This work was not funded.

## 7 Ethics statement

No original patient data were used in this review. The review data and R script with the statistical code are available in the supplementary files.

## CRediT authorship contribution statement

**J.W. Taco Boltje:** Writing – original draft, Methodology. **Mathijs T. Carvalho Mota:** Writing – original draft, Methodology. **Michiel D. Vriesendorp:** Writing – review & editing, Supervision, Conceptualization. **Alexander B.A. Vonk:** Writing – review & editing. **Rolf H.H. Groenwold:** Writing – review & editing, Supervision. **Robert J.M. Klautz:** Writing – review & editing, Supervision. **Bart J.J. Velders:** Writing – review & editing, Supervision, Conceptualization.

## Declaration of competing interest

The authors declare that they have no known competing financial interests or personal relationships that could have appeared to influence the work reported in this paper.
