# Supplementary material for: The use of pledget-reinforced sutures during surgical aortic valve replacement: A systematic review and meta-analysis
Source: Int J Cardiol Heart Vasc. 2024 Aug 22;54:101494. doi: 10.1016/j.ijcha.2024.101494 (PMC11387225; doi:10.1016/j.ijcha.2024.101494)
Supplement: Supplementary Data 1 [file mmc1.docx]

**Supplementary files -** **The Use of Pledget-Reinforced Sutures During Surgical Aortic Valve Replacement: a Systematic Review and Meta-Analysis**

J.W. Taco Boltje MD, Mathijs T. Carvalho Mota MD, Michiel D. Vriesendorp MD, PhD, Alexander B.A. Vonk MD, PhD, Rolf H.H. Groenwold MD, PhD, Robert J.M. Klautz MD, PhD, Bart J.J. Velders MD

Contents

[**Table S1. 30-day follow-up outcomes per included study.** 2](#_Toc158025323)

[**Table S2. Long term follow-up outcomes per included study** 3](#_Toc158025324)

[**Figure S1. Funnel plots of paravalvular leak, mortality, MPG and EOA.** 4](#_Toc158025325)

[**Figure S2. Remaining outcomes at 30-day follow-up.** 5](#_Toc158025326)

[**Figure S3. Remaining outcomes at mid-term follow-up.** 6](#_Toc158025327)

[**File S1. Search string.** 7](#_Toc158025328)

[**File S2. Data extraction forms.** 9](#_Toc158025329)

[**File S3. Risk of bias assessments.** 80](#_Toc158025330)

# **Table S1. 30-day follow-up outcomes per included study.**

| **STUDY CHARACTERISTICS** | | |  | **30-DAY OUTCOMES** | | |  |  |  |
| --- | --- | --- | --- | --- | --- | --- | --- | --- | --- |
| **Author** | **Year** | **Group** | **Sample size** | **PVL*** | **Thrombo-embolism** | **Infective endocarditis** | **Mortality** | **MPG** | **EOA** |
| Englberger *et al*. | 2005 | Pledgets | 414 |  |  |  |  |  |  |
|  |  | No pledgets | 135 |  |  |  |  |  |  |
| Nair *et al.* | 2010 | Pledgets | 43 |  |  |  |  |  |  |
|  |  | No pledgets | 83 |  |  |  |  |  |  |
| LaPar *et al*. | 2011 | Pledgets | 291 |  |  |  | 9 |  |  |
|  |  | No pledgets | 511 |  |  |  | 13 |  |  |
| Tabata *et al.* | 2014 | Pledgets | 50 |  |  |  |  |  | 1,30 |
|  |  | No pledgets | 102 |  |  |  |  |  | 1,42 |
| Ugur *et al*. | 2014 | Pledgets | 289 |  |  |  |  |  |  |
|  |  | No pledgets | 32 |  |  |  |  |  |  |
| Kim *et al*. | 2020 | Pledgets | 212 |  | 1 |  | 3 |  |  |
|  |  | No pledgets | 227 |  | 1 |  | 5 |  |  |
| Lee *et al.* | 2020 | Pledgets | 136 | 0 |  |  |  | 17,00 | 1,40 |
|  |  | No pledgets | 79 | 0 |  |  |  | 13,30 | 1,60 |
| Velders *et al.* | 2023 | Pledgets | 397 | 1 | 8 | 2 | 4 | 12,70 | 1,55 |
|  |  | No pledgets | 397 | 0 | 4 | 0 | 4 | 13,50 | 1,54 |
| Rasheed *et al.* | 2023 | Pledgets | 570 |  |  |  |  |  |  |
|  |  | No pledgets | 59 |  |  |  |  |  |  |

* includes moderate or greater PVL. PVL, paravalvular leak; MPG, mean pressure gradient; EOA, effective orifice area.

# **Table S2. Long term follow-up outcomes per included study**

| **STUDY CHARACTERISTICS** | | |  | **FOLLOW-UP OUTCOMES** | | |  |  |  |  |
| --- | --- | --- | --- | --- | --- | --- | --- | --- | --- | --- |
| **Author** | **Year** | **Group** | **Sample size** | **PVL*** | **Thrombo-embolism** | **Infective endocarditis** | **Mortality** | **MPG** | **EOA** | **Reported at:** |
| Englberger *et al*. | 2005 | Pledgets | 414 | 6 |  |  |  |  |  | 30.6m** |
|  |  | No pledgets | 135 | 6 |  |  |  |  |  | 30.6m** |
| Nair *et al.* | 2010 | Pledgets | 43 | 0 |  |  | 20 |  |  | 10y** |
|  |  | No pledgets | 83 | 10 |  |  | 32 |  |  | 10y** |
| LaPar *et al*. | 2011 | Pledgets | 291 | 4 | 6 |  |  |  |  | 82m** |
|  |  | No pledgets | 511 | 4 | 12 |  |  |  |  | 82m** |
| Tabata *et al.* | 2014 | Pledgets | 50 |  |  |  |  |  | 1,36 | 1y |
|  |  | No pledgets | 102 |  |  |  |  |  | 1,44 | 1y |
| Ugur *et al*. | 2014 | Pledgets | 289 | 1 |  |  |  | 8,98 | 1,52 | 1y |
|  |  | No pledgets | 32 | 0 |  |  |  | 9,90 | 1,40 | 1y |
| Kim *et al*. | 2020 | Pledgets | 212 |  |  |  | 5 |  | 1,74 | 1y |
|  |  | No pledgets | 227 |  |  |  | 9 |  | 1,73 | 1y |
| Lee *et al.* | 2020 | Pledgets | 136 |  |  |  |  | 14,10 | 1,40 | 9.6m** |
|  |  | No pledgets | 79 |  |  |  |  | 10,90 | 1,60 | 9.6m** |
| Velders *et al.* | 2023 | Pledgets | 397 | 1 | 22 | 20 | 45 | 12,30 | 1,35 | 5y |
|  |  | No pledgets | 397 | 0 | 22 | 15 | 37 | 12,30 | 1,44 | 5y |
| Rasheed *et al.* | 2023 | Pledgets | 570 |  |  |  |  |  |  |  |
|  |  | No pledgets | 59 |  |  |  |  |  |  |  |

* includes moderate or greater PVL. ** If no time-point was reported the median follow-up is presented. PVL, paravalvular leak; MPG, mean pressure gradient; EOA, effective orifice area; m, months; y, year.

# **Figure S1. Funnel plots of paravalvular leak, mortality, MPG and EOA.**


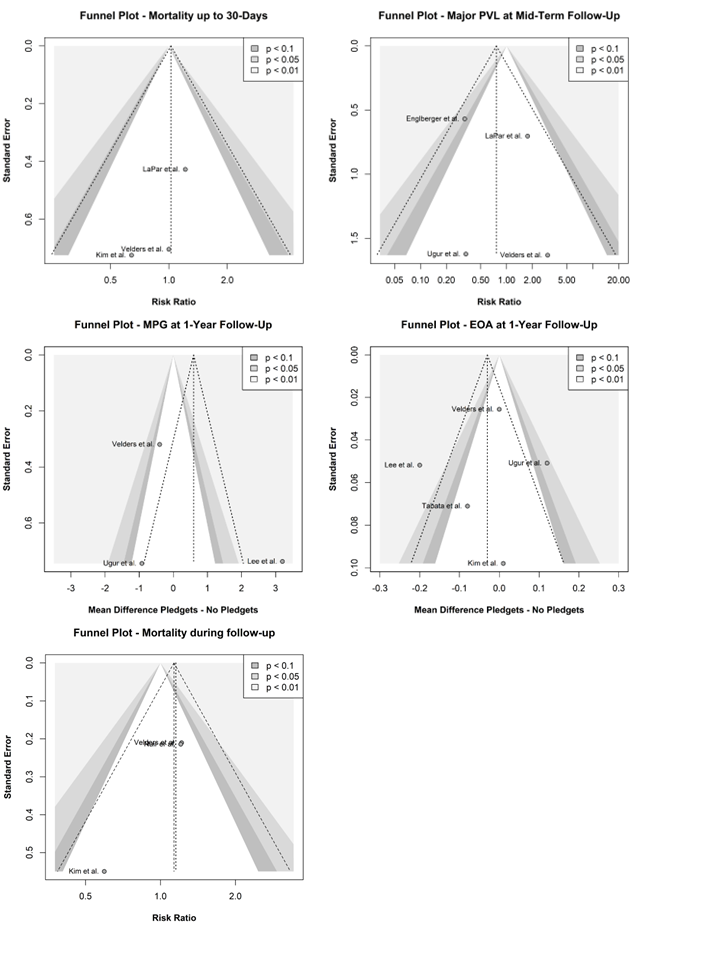


EOA, effective orifice area; MPG, mean pressure gradient; PVL, paravalvular leak.

# **Figure S2. Remaining outcomes at 30-day follow-up.**

**
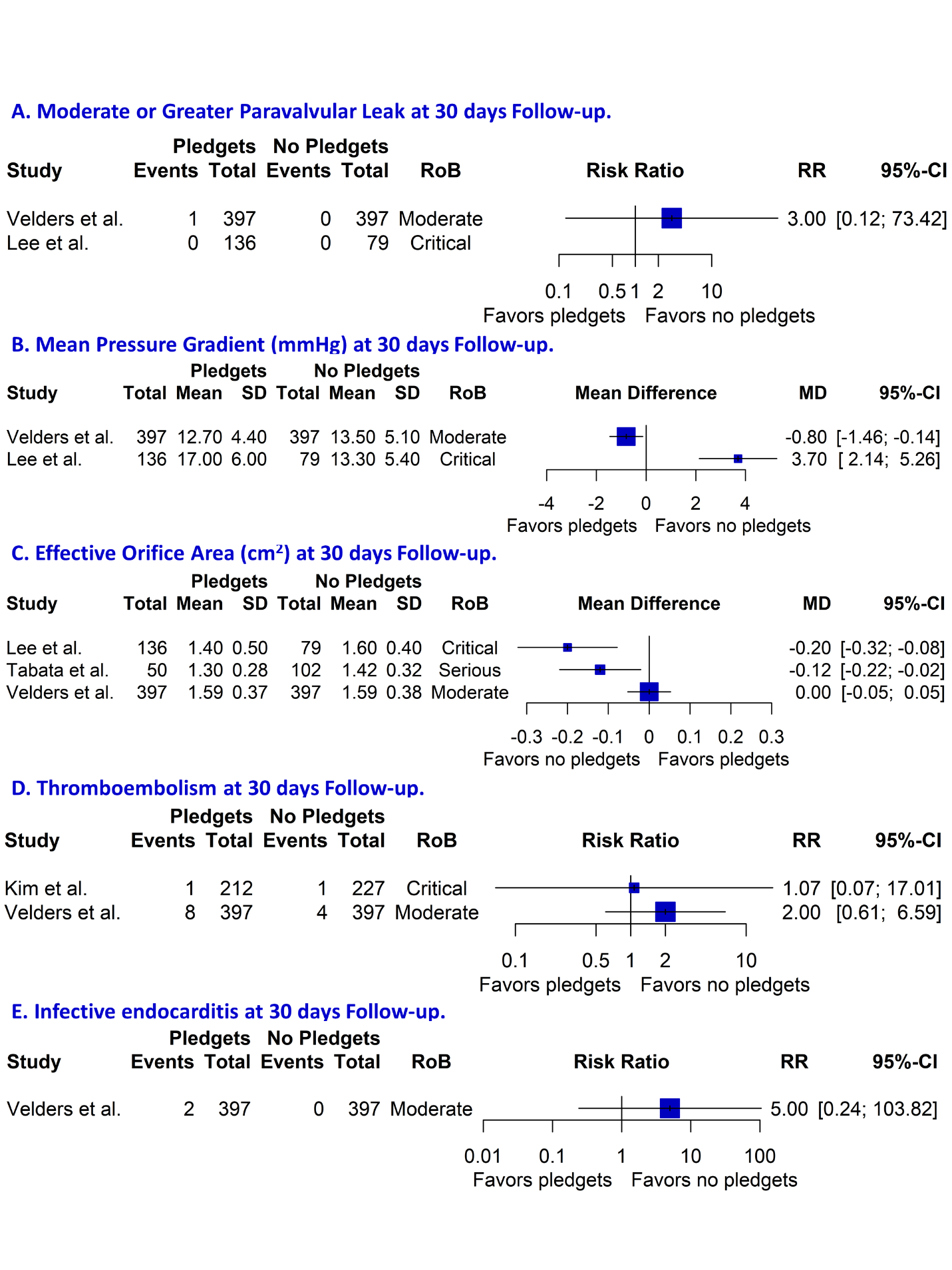
**

CI, confidence interval; SD, standard deviation; MD, mean difference; RoB, risk of bias.

# **Figure S3. Remaining outcomes at mid-term follow-up.**

**
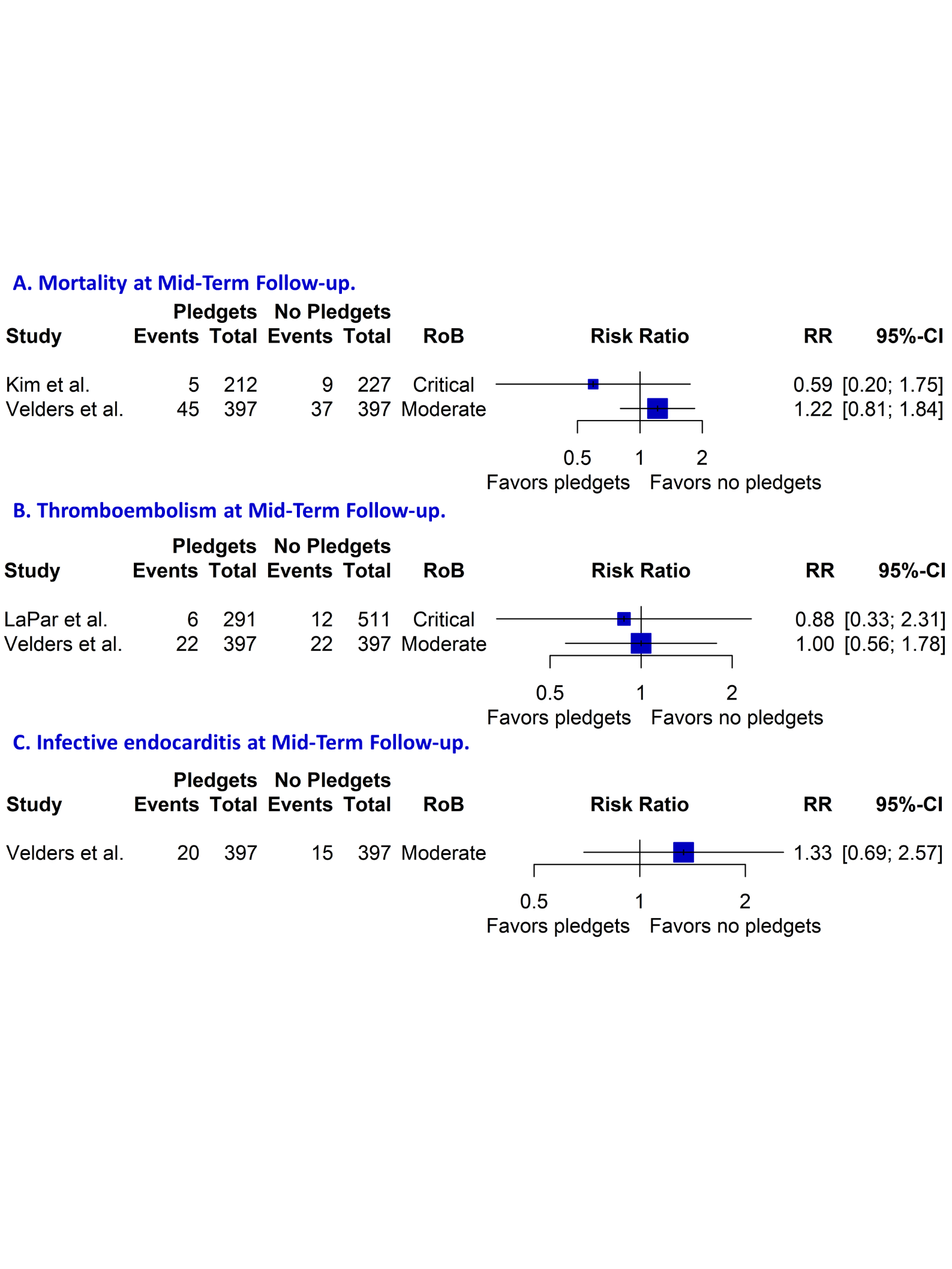
**

CI, confidence interval; MD, mean difference; RoB, risk of bias.

# **File S1. Search string.**

| Database(s): Ovid MEDLINE(R) ALL 1946 to June 06, 2023 | |  |
| --- | --- | --- |
| Search Strategy: | |  |
| # | Searches | Results |
| 1 | exp Aortic Valve/ | 39513 |
| 2 | (aortic adj3 valve* adj3 (surger* or surgical* or operation* or replac* or substitut* or reconstruct* or transplant* or grafting or implant* or repair* or correct*)).ti,ab,kf. | 34686 |
| 3 | or/1-2 | 56081 |
| 4 | exp Suture Techniques/ | 49356 |
| 5 | pledget*.ti,ab,kf. | 888 |
| 6 | (sutur* adj2 (technique* or method* or procedure* or approach*)).ti,ab,kf. | 9008 |
| 7 | or/4-6 | 54373 |
| 8 | 3 and 7 | 863 |

| Database(s): Embase Classic+Embase 1947 to 2023 June 06 | |  |
| --- | --- | --- |
| # | Searches | Results |
| 1 | exp aortic valve replacement/ | 41586 |
| 2 | (aortic adj3 valve* adj3 (surger* or surgical* or operation* or replac* or substitut* or reconstruct* or transplant* or grafting or implant* or repair* or correct*)).ti,ab,kf. | 56872 |
| 3 | or/1-2 | 66453 |
| 4 | exp suture technique/ | 7501 |
| 5 | pledget*.ti,ab,kf. | 1362 |
| 6 | (sutur* adj2 (technique* or method* or procedure* or approach*)).ti,ab,kf. | 13498 |
| 7 | or/4-6 | 20081 |
| 8 | 3 and 7 | 412 |

| Wednesday, June 07, 2023 8:39:23 AM Database - CINAHL Plus with Full Tekst | |  |
| --- | --- | --- |
| # | Query | Results |
| S8 | (S4 OR S5 OR S6) AND (S3 AND S7) | 122 |
| S7 | S4 OR S5 OR S6 | 10.427 |
| S6 | TX (sutur* N2 (technique* or method* or procedure* or approach*)) | 8.795 |
| S5 | TX pledget* | 331 |
| S4 | (MH "Suture Techniques+") | 8.188 |
| S3 | S1 OR S2 | 14.018 |
| S2 | TX (aortic N3 valve* N3 (surger* or surgical* or operation* or replac* or substitut* or reconstruct* or transplant* or grafting or implant* or repair* or correct*)) | 12.984 |
| S1 | (MM "Aortic Valve") | 3.499 |

| Search Name: | 230602 T Boltjes SR pledglet surtering in aotic valve repacement |  |
| --- | --- | --- |
| Date Run: | 6/7/2023 9:42 |  |
| ID | Search | Hits |
| #1 | MeSH descriptor: [Aortic Valve] explode all trees | 720 |
| #2 | (aortic NEAR/3 valve* NEAR/3 (surger* or surgical* or operation* or replac* or substitut* or reconstruct* or transplant* or grafting or implant* or repair* or correct*)) | 2265 |
| #3 | {or #1-#2} | 2430 |
| #4 | MeSH descriptor: [Suture Techniques] explode all trees | 2440 |
| #5 | pledget* | 106 |
| #6 | (sutur* NEAR/2 (technique* or method* or procedure* or approach*)) | 3643 |
| #7 | {or #4-#6} | 4066 |
| #8 | #3 AND #7 | 24 trials |

| # Web of Science Search Strategy (v0.1) Date Run: Wed Jun 07 2023  # Database: Web of Science Core Collection  - WOS.SCI: 1975 to 2023  - WOS.AHCI: 1975 to 2023  - WOS.ESCI: 2005 to 2023  - WOS.SSCI: 1975 to 2023 | |
| --- | --- |
| # Searches: |  |
| 1: TS=(aortic NEAR/3 valve* NEAR/3 (surger* or surgical* or operation* or replac* or substitut* or reconstruct* or transplant* or grafting or implant* or repair* or correct*)) | Results: 39288 |
| 2: TS=( pledget* or (sutur* NEAR/2 (technique* or method* or procedure* or approach*))) | Results: 10824 |
| 3: #1 AND #2 | Results: 214 |

# **File S2. Data extraction forms.**

Data extraction form:

The Use of Pledget-Reinforced Sutures During Surgical Aortic Valve Replacement: a Systematic Review and Meta-Analysis

Notes on using data extraction form:

Be consistent in the order and style you use to describe the information for each report.

Record any missing information as unclear or not described, to make it clear that the information was not found in the study report(s), not that you forgot to extract it.

Include any instructions and decision rules on the data collection form, or in an accompanying document. It is important to practice using the form and give training to any other authors using the form.

| Review title or ID | Importance of implant technique on risk of major paravalvular leak (PVL) after St. Jude mechanical heart valve replacement: a report from the Artificial Valve Endocarditis Reduction Trial (AVERT) |
| --- | --- |
| Study ID *(surname of first author and year first full report of study was published e.g. Smith 2001)* | Englberger et al. (2005) |
| Report ID |  |
| Report ID of other reports of this study |  |
| Notes | |

General Information

| Date form completed *(dd/mm/yyyy)* | 14-09-2023 |
| --- | --- |
| Name/ID of person extracting data | Taco Boltje |
| Reference citation | Englberger, L., Schaff, H. V., Jamieson, W. R., Kennard, E. D., Im, K. A., Holubkov, R., Carrel, T. P., & AVERT Investigators (2005). Importance of implant technique on risk of major paravalvular leak (PVL) after St. Jude mechanical heart valve replacement: a report from the Artificial Valve Endocarditis Reduction Trial (AVERT). *European journal of cardio-thoracic surgery : official journal of the European Association for Cardio-thoracic Surgery*, *28*(6), 838–843. https://doi.org/10.1016/j.ejcts.2005.09.014 |
| Study author contact details |  |
| Publication type *(e.g. full report, abstract, letter)* | Full text |
| Notes: | |

Study eligibility

| Study Characteristics | Eligibility criteria | | Eligibility criteria met? | | | Location in text or source *(pg & ¶/fig/table/other)* |
| --- | --- | --- | --- | --- | --- | --- |
|  |  | | Yes | No | Unclear |  |
| Type of study | Randomised Controlled Trial / Observational study | |  |  |  | p. 839 study protocol |
| Language | English | |  |  |  |  |
| Participants | Patients undergoing SAVR | |  |  |  | p. 839 study protocol |
| Types of comparison | Pledget-reinforced suturing technique with suturing technique without pledgets | |  |  |  | Table 7. |
| INCLUDE | | EXCLUDE | | | | |
| Reason for exclusion |  | | | | | |
| Notes: Both aortic and mitral valve replacement included, no subgroup analyses for AVR alone. | | | | | | |

**DO NOT PROCEED IF STUDY EXCLUDED FROM REVIEW**

Characteristics of included studies

Methods

|  | **Descriptions as stated in report/paper** | | **Location in text or source** *(pg & ¶/fig/table/other)* |
| --- | --- | --- | --- |
| **Aim of study** *(e.g. efficacy, equivalence, pragmatic)* | Usint the AVERT database to examine risk factor for major PVL after mechanical heart valve replacement | | p. 839 introduction |
| **Design***(e.g. parallel, crossover, non-RCT)* | RCT (secondary analysis) | | p. 839 study protocol |
| **Unit of allocation**  *(by individuals, cluster/ groups or body parts)* | Individuals | | p. 839 study protocol |
| **Start date** | July 1998 | | p. 839 study protocol |
| **End date** | January 2000 | | p. 839 study protocol |
| **Duration of participation**  *(from recruitment to last follow-up)* | 3652 valve years (1837 and 1815 years for silzone and conventional cuffed valves, respectively). | | p. 839 patient population |
| **Ethical approval needed/ obtained for study** | Yes No Unclear |  |  |
| **Notes:** | | | |

Participants

|  | Description  *Include comparative information for each intervention or comparison group if available* | | Location in text or source *(pg & ¶/fig/table/other)* |
| --- | --- | --- | --- |
| Setting  *(including location and social context)* | 807 patients undergoing surgical valve replacement within 12 North-American and 7 European centres. | | p. 839 study protocol |
| Inclusion criteria | Patients requiring replacement of the aortic and/or mitral valve with a mechanical prosthesis | | p. 839 study protocol |
| Exclusion criteria | The presence of prosthetic valve that do not require replacement and the need for tricuspid valve replacement | | Initial paper AVERT trial. |
| Informed consent obtained | Yes No Unclear | Reported in initial papers of AVERT trial, informed consent obtained. |  |
| Total no. randomised  *(or total pop. at start of study for NRCTs)* | 807 | | p. 839 study protocol |
| Sample size per treatment arm  *(if applicable, no., type, no. people per cluster)* | Silzone = 403, conventional = 404  549 aortic valve (75.4% pledgets used) | | p. 839 patient population  p.840 table 5 |
| Baseline imbalances | Not reported | |  |
| Withdrawals and exclusions  *(if not provided below by outcome)* | Three major PVL associated with endocarditis were excluded from the analysis | | p. 839 |
| Age *(mean)* | 61.3 +- 10.6 | | Table 1 |
| Sex *(percentage)* | Male 58.9% | | Table 1 |
| Follow-up length | Mean follow up was 30.6 months | | p. 838 Abtract |
| Notes: | | | |

Intervention groups

*Copy and paste table for each intervention and comparison group*

**Intervention Group 1**

|  | Description as stated in report/paper | Location in text or source *(pg & ¶/fig/table/other)* |
| --- | --- | --- |
| Group name | Pledgets | p. 839 patient population |
| No. randomised to group  *(specify whether no. people or clusters)* | 414 | p. 839 patient population |
| Type of prosthetic valve implanted (mechanical or biological) | Mechanical | p. 839 study protocol |
| Size of prosthetic valve implanted | N/A |  |
| Confounders adjusted for | Pledgets | P. 841 table 7 |
| Adjustment method | N/A |  |
| Notes: | | |

**Comparisson Group 1**

|  | Description as stated in report/paper | Location in text or source *(pg & ¶/fig/table/other)* |
| --- | --- | --- |
| Group name | Non-pledgets | P. 839 patient population |
| No. randomised to group  *(specify whether no. people or clusters)* | 135 | p. 839 patient population |
| Type of prosthetic valve implanted (mechanical or biological) | Mechanical | p. 839 study protocol |
| Size of prosthetic valve implanted | N/A |  |
| Confounders adjusted for |  | P. 841 table 7 |
| Adjustment method | N/A |  |
| Notes: | | |

Outcomes

*Copy and paste table for each outcome.*

**Primary outcome: parvalvular leak**

|  | Description as stated in report/paper | Location in text or source *(pg & ¶/fig/table/other)* |
| --- | --- | --- |
| Outcome name | Major paravalvular leak | P. 841 table 6 |
| Time points measured  *(specify whether from start or end of intervention)* | N/A |  |
| Time points reported | N/A |  |
| Outcome definition *(with diagnostic criteria if relevant)* | A PVL event was collected per study protocol as a non-structural dysfunction event, defined as any abnormality resulting in stenosis or regurgitation at the study value that is not intrinsic to the valve itself | P. 839 Event definition |
| Imputation of missing data *(e.g. assumptions made for ITT analysis)* | N/A |  |
| Notes: a major PVL event occurred in 5.8% (10/172) of patients with no pledget use versus only 1.7% (11/635) of patients with pledget use. (P 841 results)  6/414 (pledgets AVR) 6/135 (non pledgets AVR) | | |

**Secondary outcomes measured post-implantation up to 30 days and during mid-term follow-up**

**Copy table for each outcome present:**

**Thromboembolism, endocarditis, mortality, mean pressure gradient, effective orifice area**

|  | Description as stated in report/paper | Location in text or source *(pg & ¶/fig/table/other)* |
| --- | --- | --- |
| Outcome name |  |  |
| Time points measured  *(specify whether from start or end of intervention)* |  |  |
| Time points reported |  |  |
| Outcome definition *(with diagnostic criteria if relevant)* |  |  |
| Imputation of missing data *(e.g. assumptions made for ITT analysis)* |  |  |
| Notes: | | |

Other

| **Study funding sources** *(including role of funders)* | St. Jude medical | p. 842 |
| --- | --- | --- |
| **Possible conflicts of interest** *(for study authors)* |  |  |
| **Notes:** | | |

Data extraction form:

The Use of Pledget-Reinforced Sutures During Surgical Aortic Valve Replacement: a Systematic Review and Meta-Analysis

Notes on using data extraction form:

Be consistent in the order and style you use to describe the information for each report.

Record any missing information as unclear or not described, to make it clear that the information was not found in the study report(s), not that you forgot to extract it.

Include any instructions and decision rules on the data collection form, or in an accompanying document. It is important to practice using the form and give training to any other authors using the form.

| Review title or ID | Impact of suture techniques for aortic valve replacement on prosthesis-patient mismatch. |
| --- | --- |
| Study ID *(surname of first author and year first full report of study was published e.g. Smith 2001)* | Kim et al. (2020) |
| Report ID |  |
| Report ID of other reports of this study |  |
| Notes | |

General Information

| Date form completed *(dd/mm/yyyy)* | 14-09-2023 |
| --- | --- |
| Name/ID of person extracting data | Taco Boltje |
| Reference citation | Kim, H. H., Lee, S., Joo, H. C., Kim, J. H., Youn, Y. N., Yoo, K. J., & Lee, S. H. (2020). Impact of Suture Techniques for Aortic Valve Replacement on Prosthesis-Patient Mismatch. *The Annals of thoracic surgery*, *109*(3), 661–667. https://doi.org/10.1016/j.athoracsur.2019.09.012 |
| Study author contact details |  |
| Publication type *(e.g. full report, abstract, letter)* | Full text |
| Notes: | |

Study eligibility

| Study Characteristics | Eligibility criteria | | Eligibility criteria met? | | | Location in text or source *(pg & ¶/fig/table/other)* |
| --- | --- | --- | --- | --- | --- | --- |
|  |  |  | Yes | No | Unclear |  |
| Type of study | Randomised Controlled Trial / Observational study | |  |  |  | p. 662 patients |
| Language | English | |  |  |  | p. 662 patients |
| Participants | Patients undergoing SAVR | |  |  |  | p. 662 patients |
| Types of comparison | Pledget-reinforced suturing technique with suturing technique without pledgets | |  |  |  | p. 662 patients |
| INCLUDE | | EXCLUDE | | | | |
| Reason for exclusion |  | | | | | |
| Notes: | | | | | | |

**DO NOT PROCEED IF STUDY EXCLUDED FROM REVIEW**

Characteristics of included studies

Methods

|  | **Descriptions as stated in report/paper** | | **Location in text or source** *(pg & ¶/fig/table/other)* |
| --- | --- | --- | --- |
| **Aim of study** *(e.g. efficacy, equivalence, pragmatic)* | Evaluate the hemodynamic performance after AVR according to the three different suture techniques. We focused on the clinical results, including prosthesis-patient mismatch (PPM) in small-size AVR (18 to 21 mm). | | p. 662 Introduction |
| **Design***(e.g. parallel, crossover, non-RCT)* | Observational retrospective cohort | | p. 662 patients |
| **Unit of allocation**  *(by individuals, cluster/ groups or body parts)* | By individual | | p. 662 patients |
| **Start date** | January 2015 | | p. 662 patients |
| **End date** | September 2018 | | p. 662 patients |
| **Duration of participation**  *(from recruitment to last follow-up)* | Mean follow-up 16 +- 10.5 months  Mean echocardiographic follow up 12.1 +- 4.5 months | | P. 663 results |
| **Ethical approval needed/ obtained for study** | Yes No Unclear |  | p. 662 patients |
| **Notes:** | | | |

Participants

|  | Description  *Include comparative information for each intervention or comparison group if available* | | Location in text or source *(pg & ¶/fig/table/other)* |
| --- | --- | --- | --- |
| Setting  *(including location and social context)* | Yonsei university college of medicine | | p. 662 patients |
| Inclusion criteria | Patients who underwent AVR (isolated AVR, combined with CABG, mitral valve repair, tricuspid repair and aorta replacement.) | | p. 662 patients |
| Exclusion criteria | Concurrent mitral or tricuspid valve replacement. Annulus enlargement cases | | p. 662 patients |
| Informed consent obtained | Yes No Unclear | waiver | p. 662 patients |
| Total no. randomised  *(or total pop. at start of study for NRCTs)* | 439 | | p. 663 results |
| Sample size per treatment arm  *(if applicable, no., type, no. people per cluster)* | Pledgeted (n = 212), nonpledgeted (n = 122), figure-of-eight (n = 105) | | p. 663 table 1 |
| Baseline imbalances | Number of patients with hypertension and left atrial volume index | | p. 663 table 1 |
| Withdrawals and exclusions  *(if not provided below by outcome)* | 3 patients lost to follow up. 1 year follow up rate was 99.3% | | p. 663 results |
| Age *(mean)* | Per group”: 63.2 +- 13, 65.2 +- 13.1, 65.0 +-11.3 | | p. 663 table 1 |
| Sex *(percentage)* | Per group: 131 (61.8%), 71 (58.2%), 50 (47.6%) | | p. 663 table 1 |
| Follow-up length | Mean follow-up 16 +- 10.5 months  Mean echocardiographic follow up 12.1 +- 4.5 months | | p. 663 results |
| Notes: | | | |

Intervention groups

*Copy and paste table for each intervention and comparison group*

**Intervention Group 1**

|  | Description as stated in report/paper | Location in text or source *(pg & ¶/fig/table/other)* |
| --- | --- | --- |
| Group name | Pledgeted | p. 663 table 1 |
| No. randomised to group  *(specify whether no. people or clusters)* | 212 | p. 663 table 1 |
| Type of prosthetic valve implanted (mechanical or biological) | Mechanical (57.8%) and biological | Appendix 1.1 |
| Size of prosthetic valve implanted | 18-21 mm 117 (55.2%), > 21 mm 95 (44.8%) | p. 664 table 3 |
| Confounders adjusted for | Only for PPM not for other outcome: Age at implant > 70 years, Sex, NYHA class > III, number of suture stitches > 16 | P. 665 table 4 |
| Adjustment method | Univariate and multivariate analysis. | P. 665 table 4 |
| Notes: | | |

**Comparison group 1**

|  | Description as stated in report/paper | Location in text or source *(pg & ¶/fig/table/other)* |
| --- | --- | --- |
| Group name | Non-Pledgeted | p. 663 table 1 |
| No. randomised to group  *(specify whether no. people or clusters)* | 122 | p. 663 table 1 |
| Type of prosthetic valve implanted (mechanical or biological) | Mechanical (45.2%) and biological | Appendix 1.1 |
| Size of prosthetic valve implanted | 18-21 mm 73 (59.8%), > 21 mm 49 (40.2%) | p. 664 table 3 |
| Confounders adjusted for | Only for PPM not for other outcome: Age at implant > 70 years, Sex, NYHA class > III, number of suture stitches > 16 | P. 665 table 4 |
| Adjustment method | Univariate and multivariate analysis. | P. 665 table 4 |
| Notes: | | |

**Comparison group 2**

|  | Description as stated in report/paper | Location in text or source *(pg & ¶/fig/table/other)* |
| --- | --- | --- |
| Group name | Figure-of-eight | p. 663 table 1 |
| No. randomised to group  *(specify whether no. people or clusters)* | 105 | p. 663 table 1 |
| Type of prosthetic valve implanted (mechanical or biological) | Mechanical and biological | Appendix 1.1 |
| Size of prosthetic valve implanted | 18-21 mm 52 (49.5%), > 21 mm 53 (50.5%) | p. 664 table 3 |
| Confounders adjusted for | Only for PPM not for other outcome: Age at implant > 70 years, Sex, NYHA class > III, number of suture stitches > 16 | P. 665 table 4 |
| Adjustment method | Univariate and multivariate analysis. | P. 665 table 4 |
| Notes: | | |

Outcomes

*Copy and paste table for each outcome.*

**Secondary outcomes measured post-implantation up to 30 days and during mid-term follow-up**

**Copy table for each outcome present:**

**Thromboembolism, endocarditis, mortality, mean pressure gradient, effective orifice area**

|  | Description as stated in report/paper | Location in text or source *(pg & ¶/fig/table/other)* |
| --- | --- | --- |
| Outcome name | In-hospital mortality | Appendix 2 |
| Time points measured  *(specify whether from start or end of intervention)* | In-hospital, until discharge | Appendix 2 |
| Time points reported | 1 |  |
| Outcome definition *(with diagnostic criteria if relevant)* | Mortality |  |
| Imputation of missing data *(e.g. assumptions made for ITT analysis)* | N/A |  |
| Notes:  Variables Pledgeted (n=212) Non-pledgeted (n=122) Figure-of-8 (n=105) p Value  In-hospital mortality 3 (1.4%) 2 (1.6%) 3 (2.9%) 0.621  All cause mortality 5 (2.4%) 3 (2.5%) 6 (5.7%) 0.282 | | |

|  | Description as stated in report/paper | Location in text or source *(pg & ¶/fig/table/other)* |
| --- | --- | --- |
| Outcome name | EOA | p. 664 table 3 |
| Time points measured  *(specify whether from start or end of intervention)* | N/A |  |
| Time points reported | N/A | p. 664 table 3 |
| Outcome definition *(with diagnostic criteria if relevant)* | Effective orifice area In cm2 | p. 664 table 3 |
| Imputation of missing data *(e.g. assumptions made for ITT analysis)* | N/A |  |
| Notes: Table 3.  18-21 mm valves EOA, cm2 1.7 +- 1.8 1.9 +- 0.2 1.5 +- 0.3 p-value = .45  >21 mm valves EOA, cm2 1.7 +- 0.3 1.5 +- 0.2 1.5 +- 0.3 p-value = .40  Mean EOA pledgeted suture 1.74±1.38 non-pledgeted 1.73±0.39 p-value = 0.491 (appendix 3) | | |

Other

| **Study funding sources** *(including role of funders)* |  |  |
| --- | --- | --- |
| **Possible conflicts of interest** *(for study authors)* |  |  |
| **Notes:** | | |

Data extraction form:

The Use of Pledget-Reinforced Sutures During Surgical Aortic Valve Replacement: a Systematic Review and Meta-Analysis

Notes on using data extraction form:

Be consistent in the order and style you use to describe the information for each report.

Record any missing information as unclear or not described, to make it clear that the information was not found in the study report(s), not that you forgot to extract it.

Include any instructions and decision rules on the data collection form, or in an accompanying document. It is important to practice using the form and give training to any other authors using the form.

| Review title or ID | Use of a nonpledgeted suture technique is safe and efficient for aortic valve replacement |
| --- | --- |
| Study ID *(surname of first author and year first full report of study was published e.g. Smith 2001)* | LaPar et al. 2011 |
| Report ID |  |
| Report ID of other reports of this study |  |
| Notes | |

General Information

| Date form completed *(dd/mm/yyyy)* | 12-9-2023 |
| --- | --- |
| Name/ID of person extracting data | Taco Boltje |
| Reference citation | LaPar, D. J., Ailawadi, G., Bhamidipati, C. M., Singh, M., Dare, D., Kern, J. A., & Kron, I. L. (2011). Use of a nonpledgeted suture technique is safe and efficient for aortic valve replacement. *The Journal of thoracic and cardiovascular surgery*, *141*(2), 388–393. https://doi.org/10.1016/j.jtcvs.2010.04.011 |
| Study author contact details |  |
| Publication type *(e.g. full report, abstract, letter)* | Full text |
| Notes: | |

Study eligibility

| Study Characteristics | Eligibility criteria | | Eligibility criteria met? | | | Location in text or source *(pg & ¶/fig/table/other)* |
| --- | --- | --- | --- | --- | --- | --- |
|  |  |  | Yes | No | Unclear |  |
| Type of study | Randomised Controlled Trial / Observational study | |  |  |  | p. 389 |
| Language | English | |  |  |  | p. 389 |
| Participants | Patients undergoing SAVR | |  |  |  | p. 389 (operative technique) |
| Types of comparison | Pledget-reinforced suturing technique with suturing technique without pledgets | |  |  |  | p. 389 (operative technique) |
| INCLUDE | | EXCLUDE | | | | |
| Reason for exclusion |  | | | | | |
| Notes: | | | | | | |

**DO NOT PROCEED IF STUDY EXCLUDED FROM REVIEW**

Characteristics of included studies

Methods

|  | **Descriptions as stated in report/paper** | | **Location in text or source** *(pg & ¶/fig/table/other)* |
| --- | --- | --- | --- |
| **Aim of study** *(e.g. efficacy, equivalence, pragmatic)* | Equivalence in operative mortality, major PVL, subsequent aortic valve reoperation | | p. 388 introduction |
| **Design***(e.g. parallel, crossover, non-RCT)* | Retrospective cohort | | p. 388 materials and methdos |
| **Unit of allocation**  *(by individuals, cluster/ groups or body parts)* | By individuals | | p. 388/389 materials and methdos |
| **Start date** | January 1995 | | p. 388 patients |
| **End date** | April 2009 | | p. 388 patients |
| **Duration of participation**  *(from recruitment to last follow-up)* | Mean patient follow-up was 82.0 +- 1.9 months | | p. 389 comparison of early and late outcmes |
| **Ethical approval needed/ obtained for study** | Yes No Unclear |  |  |
| **Notes:** | | | |

Participants

|  | Description  *Include comparative information for each intervention or comparison group if available* | | Location in text or source *(pg & ¶/fig/table/other)* |
| --- | --- | --- | --- |
| Setting  *(including location and social context)* | University of Virginia, USA | | p. 388 patients |
| Inclusion criteria | Isolated AVR | | p. 388 patients |
| Exclusion criteria | No | | p. 389 patients |
| Informed consent obtained | Yes No Unclear | Approved by human invistigation committee | p. 389 patients |
| Total no. randomised  *(or total pop. at start of study for NRCTs)* | 802 | | p. 389 results |
| Sample size per treatment arm  *(if applicable, no., type, no. people per cluster)* | 511 non-pledgeted vs 291 pledgeted | | p. 389 results |
| Baseline imbalances | Age, hypertension, stroke, dyslipidemia, AF, previous CABG, ejection fraction, Aortic stenosis, aortic insufficiency, type of prosthesis. Crossclamp time, bypass time. | | Table 1. |
| Withdrawals and exclusions  *(if not provided below by outcome)* | N/A | |  |
| Age *(mean)* | 67.0 +- 0.7 (nonpledgeted), 62.7 +- 1.5 (pledgeted) | | Table 1 |
| Sex *(percentage)* | Female 200 (39.1%) (nonpledgeted), 122 (41.9%) (pledgeted) | | Table 1 |
| Follow-up length | Mean patient follow-up was 82.0 +- 1.9 months | | p. 389 outcomes |
| Notes: | | | |

Intervention groups

*Copy and paste table for each intervention and comparison group*

**Intervention Group 1**

|  | Description as stated in report/paper | Location in text or source *(pg & ¶/fig/table/other)* |
| --- | --- | --- |
| Group name | Non-pledgeted | Table 1. |
| No. randomised to group  *(specify whether no. people or clusters)* | 511 | Table 1. |
| Type of prosthetic valve implanted (mechanical or biological) | Bioprosthesis 431, and mechanical 77, homograft 3 | Table 1. |
| Size of prosthetic valve implanted | N/A |  |
| Confounders adjusted for | Primary vs non primary AVR | P. 391 results |
| Adjustment method | Stratification | p. 391 results |
| Notes: | | |

**Comparisson Group 1**

|  | Description as stated in report/paper | Location in text or source *(pg & ¶/fig/table/other)* |
| --- | --- | --- |
| Group name | Pledgeted | Table 1. |
| No. randomised to group  *(specify whether no. people or clusters)* | 259 | Table 1. |
| Type of prosthetic valve implanted (mechanical or biological) | Bioprosthesis 160, and mechanical 122, homograft 9 | Table 1. |
| Size of prosthetic valve implanted | N/A |  |
| Confounders adjusted for | Primary vs non primary AVR | P. 391 results |
| Adjustment method | Stratification | p. 391 results |
| Notes: | | |

Outcomes

*Copy and paste table for each outcome.*

**Primary outcome: parvalvular leak**

|  | Description as stated in report/paper | Location in text or source *(pg & ¶/fig/table/other)* |
| --- | --- | --- |
| Outcome name | PVL | Table 3 |
| Time points measured  *(specify whether from start or end of intervention)* | Early 30 days and late follow up, avg 82 months | p. 389 comparison of early and late outcomes |
| Time points reported | 51.3 +- 16.7 months | p. 389 comparison of early and late outcomes |
| Outcome definition *(with diagnostic criteria if relevant)* | PVL |  |
| Imputation of missing data *(e.g. assumptions made for ITT analysis)* | N/A |  |
| Notes:  Nonpledgeted 4 (0.8%) vs pledgeted 4 (1.4%) p=.47 | | |

**Secondary outcomes measured post-implantation up to 30 days and during mid-term follow-up**

**Copy table for each outcome present:**

**Thromboembolism, endocarditis, mortality, mean pressure gradient, effective orifice area**

|  | Description as stated in report/paper | Location in text or source *(pg & ¶/fig/table/other)* |
| --- | --- | --- |
| Outcome name | Mortality | Table 3 |
| Time points measured  *(specify whether from start or end of intervention)* | Early 30 days and late follow up, avg 82 months | p. 389 comparison of early and late outcomes |
| Time points reported | 82.0 +- 1.9 months | p. 389 comparison of early and late outcomes |
| Outcome definition *(with diagnostic criteria if relevant)* | Operative mortality | Table 3 |
| Imputation of missing data *(e.g. assumptions made for ITT analysis)* | - |  |
| Notes:      Nonpledgeted = 13 (2.5%), 9 (3.1%) p .66 | | |

|  | Description as stated in report/paper | Location in text or source *(pg & ¶/fig/table/other)* |
| --- | --- | --- |
| Outcome name | Thromboembolism | Table 3 |
| Time points measured  *(specify whether from start or end of intervention)* | Early 30 days and late follow up, avg 82 months | p. 389 comparison of early and late outcomes |
| Time points reported | 82.0 +- 1.9 months | p. 389 comparison of early and late outcomes |
| Outcome definition *(with diagnostic criteria if relevant)* | Stroke | Table 3 |
| Imputation of missing data *(e.g. assumptions made for ITT analysis)* | - |  |
| Notes:      Nonpledgeted = 12 (2.3%), 6 (2.1%) p >.99 | | |

Other

| **Study funding sources** *(including role of funders)* | N/A |  |
| --- | --- | --- |
| **Possible conflicts of interest** *(for study authors)* | N/A |  |
| **Notes:** | | |

Data extraction form:

The Use of Pledget-Reinforced Sutures During Surgical Aortic Valve Replacement: a Systematic Review and Meta-Analysis

Notes on using data extraction form:

Be consistent in the order and style you use to describe the information for each report.

Record any missing information as unclear or not described, to make it clear that the information was not found in the study report(s), not that you forgot to extract it.

Include any instructions and decision rules on the data collection form, or in an accompanying document. It is important to practice using the form and give training to any other authors using the form.

| Review title or ID | Simple Interrupted Suturing for Aortic Valve Replacement in Patients with Severe Aortic Stenosis |
| --- | --- |
| Study ID *(surname of first author and year first full report of study was published e.g. Smith 2001)* | Lee et al. (2020) |
| Report ID |  |
| Report ID of other reports of this study |  |
| Notes | |

General Information

| Date form completed *(dd/mm/yyyy)* | 12-9-2023 |
| --- | --- |
| Name/ID of person extracting data | Taco Boltje |
| Reference citation | Lee, J. O., Lee, C. H., Kim, H. J., Kim, J. B., Jung, S. H., Joo, S. J., Chung, C. H., & Lee, J. W. (2020). Simple Interrupted Suturing for Aortic Valve Replacement in Patients with Severe Aortic Stenosis. *The Korean journal of thoracic and cardiovascular surgery*, *53*(6), 332–338. https://doi.org/10.5090/kjtcs.20.066 |
| Study author contact details |  |
| Publication type *(e.g. full report, abstract, letter)* | Full text |
| Notes: | |

Study eligibility

| Study Characteristics | Eligibility criteria | | Eligibility criteria met? | | | Location in text or source *(pg & ¶/fig/table/other)* |
| --- | --- | --- | --- | --- | --- | --- |
|  |  |  | Yes | No | Unclear |  |
| Type of study | Randomised Controlled Trial / Observational study | |  |  |  | P. 333 study population |
| Language | English | |  |  |  |  |
| Participants | Patients undergoing SAVR | |  |  |  | P. 333 Study population |
| Types of comparison | Pledget-reinforced suturing technique with suturing technique without pledgets | |  |  |  | P. 333 Surgical procedures |
| INCLUDE | | EXCLUDE | | | | |
| Reason for exclusion |  | | | | | |
| Notes: | | | | | | |

**DO NOT PROCEED IF STUDY EXCLUDED FROM REVIEW**

Characteristics of included studies

Methods

|  | **Descriptions as stated in report/paper** | | **Location in text or source** *(pg & ¶/fig/table/other)* |
| --- | --- | --- | --- |
| **Aim of study** *(e.g. efficacy, equivalence, pragmatic)* | Comparing hemodynamic outcomes of the concentional NMS and SIS techniques after AVR to test the hypothesis that the SIS technique could be a more reasonable option to achieve better EOA after AVR. | | p. 333 introduction |
| **Design***(e.g. parallel, crossover, non-RCT)* | Non-rct retrospective cohort | | p. 333 study population |
| **Unit of allocation**  *(by individuals, cluster/ groups or body parts)* | By individuals | | p. 333 study population |
| **Start date** | March 2015 | | p. 333 study population |
| **End date** | November 2016 | | p. 333 study population |
| **Duration of participation**  *(from recruitment to last follow-up)* | Median follow-up was 9.6 months (IQR 5.8 – 12.9 months) | | p. 333 outcomes of interest and data collection |
| **Ethical approval needed/ obtained for study** | Yes No Unclear | Approved by review board | p. 333 study population |
| **Notes:** | | | |

Participants

|  | Description  *Include comparative information for each intervention or comparison group if available* | | Location in text or source *(pg & ¶/fig/table/other)* |
| --- | --- | --- | --- |
| Setting  *(including location and social context)* | Asian medical centre Seoul Korea. | | Title. |
| Inclusion criteria | Patients with severe AS who underwent AVR with a supra-annular prosthesis between March 2015 and November 2016 | | p. 333 study population |
| Exclusion criteria | Patients who underwent concomitant mitral and tricuspid valve replacement, redo aortic valve surgery, and AVR using other suture techniques | | p. 333 study population |
| Informed consent obtained | Yes No Unclear | Waiver | p. 333 study population |
| Total no. randomised  *(or total pop. at start of study for NRCTs)* | 215 | | p. 333 study population |
| Sample size per treatment arm  *(if applicable, no., type, no. people per cluster)* | SIS technique = 79 patients  NMS technique = 136 patients | | p. 333 study population |
| Baseline imbalances | Age differed (p-value = 0.016) | | p. 334 results |
| Withdrawals and exclusions  *(if not provided below by outcome)* | - | |  |
| Age *(mean)* | SIS = 65.1 +- 9.7 NMS = 68.0 +- 7.9 | | Table 1 |
| Sex *(percentage)* | Male is SIS = 46 (58.2%) and NMS = 88 (64.7%) | | Table 1 |
| Follow-up length | Second follow up 9.6 (iqr 5.8 – 12.9 months)  Third follow up 26 months (iqr 8 – 46 months) | | Table 3 |
| Notes: | | | |

Intervention groups

*Copy and paste table for each intervention and comparison group*

**Intervention Group 1**

|  | Description as stated in report/paper | Location in text or source *(pg & ¶/fig/table/other)* |
| --- | --- | --- |
| Group name | Non-everting mattress suture (NMS) | Table 2. |
| No. randomised to group  *(specify whether no. people or clusters)* | 136 | Table 2. |
| Type of prosthetic valve implanted (mechanical or biological) | 41 mechanical valves  95 bioprosthesis | Table 2. |
| Size of prosthetic valve implanted | 18-20 mm = 21  21-22 mm = 51  23-24 mm = 39  25-27 mm = 25 | Table 2. |
| Confounders adjusted for | Annulus size, suture techniqe | Methods |
| Adjustment method | Stratification | Methods |
| Notes: | | |

**Comparison group 1**

|  | Description as stated in report/paper | Location in text or source *(pg & ¶/fig/table/other)* |
| --- | --- | --- |
| Group name | Simple interrupted suture (SIS) | Table 2. |
| No. randomised to group  *(specify whether no. people or clusters)* | 79 | Table 2. |
| Type of prosthetic valve implanted (mechanical or biological) | 33 mechanical valves  46 bioprosthesis | Table 2. |
| Size of prosthetic valve implanted | 18-20 mm = 7  21-22 mm = 20  23-24 mm = 29  25-27 mm = 23 | Table 2. |
| Confounders adjusted for | Annulus size, suture techniqe | Methods |
| Adjustment method | Stratification | Methods |
| Notes: | | |

Outcomes

*Copy and paste table for each outcome.*

**Secondary outcomes measured post-implantation up to 30 days and during mid-term follow-up**

**Copy table for each outcome present:**

**Thromboembolism, endocarditis, mortality, mean pressure gradient, effective orifice area**

|  | Description as stated in report/paper | Location in text or source *(pg & ¶/fig/table/other)* |
| --- | --- | --- |
| Outcome name | Mean pressure gradient (mm Hg) | Table 3 |
| Time points measured  *(specify whether from start or end of intervention)* | Post-operative EC (within 5 days)  Second follow up (9.6 months (iqr, 5.8 – 12.9 months) | Table 3 |
| Time points reported | SIS = 13.3 +- 5.4 NMS = 17.0 +- 6.0  SIS = 10.9 +-5.1 NMS = 14.1 +- 5.4 | Table 3 |
| Outcome definition *(with diagnostic criteria if relevant)* | Mean pressure gradient in mm Hg | Table 3 |
| Imputation of missing data *(e.g. assumptions made for ITT analysis)* | No missing data |  |
| Notes: subgroup analysis for prosthetic valve size (table 4) | | |

|  | Description as stated in report/paper | Location in text or source *(pg & ¶/fig/table/other)* |
| --- | --- | --- |
| Outcome name | EOA (cm2) | Table 3 |
| Time points measured  *(specify whether from start or end of intervention)* | Post-operative EC (within 5 days)  Second follow up (9.6 months (iqr, 5.8 – 12.9 months) | Table 3 |
| Time points reported | SIS = 1.6 +-0.4 NMS = 1.4 +-0.5  SIS = 1.6 +-0.4 NMS = 1.4 +-0.3 | Table 3 |
| Outcome definition *(with diagnostic criteria if relevant)* | Effective orifice area cm2, continuity equation | Table 3 |
| Imputation of missing data *(e.g. assumptions made for ITT analysis)* | No missing data |  |
| Notes: subgroup analysis for prosthetic valve size (table 4) | | |

Other

| **Study funding sources** *(including role of funders)* | Not provided |  |
| --- | --- | --- |
| **Possible conflicts of interest** *(for study authors)* | No | p. 338 |
| **Notes:** | | |

Data extraction form:

The Use of Pledget-Reinforced Sutures During Surgical Aortic Valve Replacement: a Systematic Review and Meta-Analysis

Notes on using data extraction form:

Be consistent in the order and style you use to describe the information for each report.

Record any missing information as unclear or not described, to make it clear that the information was not found in the study report(s), not that you forgot to extract it.

Include any instructions and decision rules on the data collection form, or in an accompanying document. It is important to practice using the form and give training to any other authors using the form.

| Review title or ID | Figure of eight suture technique in aortic valve replacement decreases prosthesis-patient |
| --- | --- |
| Study ID *(surname of first author and year first full report of study was published e.g. Smith 2001)* | Rasheed et al. (2023) |
| Report ID |  |
| Report ID of other reports of this study |  |
| Notes | |

General Information

| Date form completed *(dd/mm/yyyy)* | 8-9-23 |
| --- | --- |
| Name/ID of person extracting data | Taco Boltje |
| Reference citation | Rasheed NF, Stonebraker C, Li Z, *et al.* Figure of eight suture technique in aortic valve replacement decreases prosthesis-patient mismatch. *J Cardiothorac Surg*. 2023;18(1):117 |
| Study author contact details |  |
| Publication type *(e.g. full report, abstract, letter)* | Full text |
| Notes: | |

Study eligibility

| Study Characteristics | Eligibility criteria | | Eligibility criteria met? | | | Location in text or source *(pg & ¶/fig/table/other)* |
| --- | --- | --- | --- | --- | --- | --- |
|  |  |  | Yes | No | Unclear |  |
| Type of study | Randomised Controlled Trial / Observational study | |  |  |  | Abstract |
| Language | English | |  |  |  | Abstract |
| Participants | Patients undergoing SAVR | |  |  |  | Abstract |
| Types of comparison | Pledget-reinforced suturing technique with suturing technique without pledgets | |  |  |  | p. 2 surgical technique |
| INCLUDE | | EXCLUDE | | | | |
| Reason for exclusion |  | | | | | |
| Notes: | | | | | | |

**DO NOT PROCEED IF STUDY EXCLUDED FROM REVIEW**

Characteristics of included studies

Methods

|  | **Descriptions as stated in report/paper** | | **Location in text or source** *(pg & ¶/fig/table/other)* |
| --- | --- | --- | --- |
| **Aim of study** *(e.g. efficacy, equivalence, pragmatic)* | Superiority | | P. 2 introduction |
| **Design***(e.g. parallel, crossover, non-RCT)* | Retrospective non-RCT | | p. 2 introduction |
| **Unit of allocation**  *(by individuals, cluster/ groups or body parts)* | By individuals | | p. 2 patient selection |
| **Start date** | January 2011 | | p. 2 patient selection |
| **End date** | July 2018 | | p. 2 patient selection |
| **Duration of participation**  *(from recruitment to last follow-up)* | - | |  |
| **Ethical approval needed/ obtained for study** | Yes No Unclear |  | p. 2 patient selection |
| **Notes:** | | | |

Participants

|  | Description  *Include comparative information for each intervention or comparison group if available* | | Location in text or source *(pg & ¶/fig/table/other)* |
| --- | --- | --- | --- |
| Setting  *(including location and social context)* | University of chigao medical center | | p. 2 patient selection |
| Inclusion criteria | Patients undergoing surigical AVR using either pledgeted or figure-of-eigth suture technique | | p.2 patient selection |
| Exclusion criteria | TAVR, other suture technique, EOA unavailable, implanted valve size, valve manufacturer, manufacturer EOA, preoperative height or preoperative weight | | p. 2 patient selection |
| Informed consent obtained | Yes No Unclear | waived | p. 2 patient selection |
| Total no. randomised  *(or total pop. at start of study for NRCTs)* | 629 | | p. 2 patient selection |
| Sample size per treatment arm  *(if applicable, no., type, no. people per cluster)* | Pledget (n=570) figure-of-eigth (n=59) | | Table 2 |
| Baseline imbalances | Age, BSA, Smoking status, pre-op creatine, ejection fraction, AV insufficiency, LOS, LOS surgery to discharge | | Table 1 |
| Withdrawals and exclusions  *(if not provided below by outcome)* | Non | | p. 2 patient selection |
| Age *(mean)* | Pledget 70 (61, 78)     figure-of-eigth 59 (51.5, 67) | | Table 1 |
| Sex *(percentage)* | Pledget 220 (39%) figure of eigth 18 (31%) | | Table 1 |
| Follow-up length | N/A | |  |
| Notes: | | | |

Intervention groups

*Copy and paste table for each intervention and comparison group*

**Intervention Group 1**

|  | Description as stated in report/paper | Location in text or source *(pg & ¶/fig/table/other)* |
| --- | --- | --- |
| Group name | Pledget | Table 1 |
| No. randomised to group  *(specify whether no. people or clusters)* | 570 | Table 1 |
| Type of prosthetic valve implanted (mechanical or biological) | Mechanical or biological | p. 3 results |
| Size of prosthetic valve implanted | 19, 21, 23, 25, 27, 29  19-23mm: 299, 25-29mm: 271 | p. 2 data collection |
| Confounders adjusted for | Valve type and manufacturer | p. 4 |
| Adjustment method | Stratification | Table 2 |
| Notes: | | |

**Comparison Group 1**

|  | Description as stated in report/paper | Location in text or source *(pg & ¶/fig/table/other)* |
| --- | --- | --- |
| Group name | Figure-of-eight | Table 1 |
| No. randomised to group  *(specify whether no. people or clusters)* | 59 | Table 1 |
| Type of prosthetic valve implanted (mechanical or biological) | Mechanical or biological | p. 3 results |
| Size of prosthetic valve implanted | 19, 21, 23, 25, 27, 29  19-23 mm 18, 25-29 mm : 271 | p. 2 data collection |
| Confounders adjusted for | Valve type and manufacturer | p. 4 |
| Adjustment method | N/A |  |
| Notes: | | |

Outcomes

*Copy and paste table for each outcome.*

**Secondary outcomes measured post-implantation up to 30 days and during mid-term follow-up**

**Copy table for each outcome present:**

**Thromboembolism, endocarditis, mortality, mean pressure gradient, effective orifice area**

|  | Description as stated in report/paper | Location in text or source *(pg & ¶/fig/table/other)* |
| --- | --- | --- |
| Outcome name | iEOA | Table 2 |
| Time points measured  *(specify whether from start or end of intervention)* | N/A |  |
| Time points reported | 1 | Table 2 |
| Outcome definition *(with diagnostic criteria if relevant)* | Indexed effective orifice area.  Using the Cardio Valve application (© 2012 Digital Medical Networks), valve EOA was determined. Te Cardio Valve application utilizes an algorithm derived from existing aortic prosthetic valve literature and valve manufacturer information to estimate EOA for a given valve type and size. Indexed efective orifce area (iEOA) was calculated by dividing the EOA from the Cardio Valve application by the preoperative patient body surface area (BSA). | Table 2, data collection |
| Imputation of missing data *(e.g. assumptions made for ITT analysis)* | N/A |  |
| Notes:  19-23 mm iEOA, cm2 /m2 0.86 (0.77, 0.98) - 0.89 (0.82, 0.97) - 0.3  25-29 mm iEOA, cm2 /m2 1.03 (0.93, 1.15) - 1.05 (0.92, 1.15) - 0.77 | | |

Other

| **Study funding sources** *(including role of funders)* | No funding | p. 6 |
| --- | --- | --- |
| **Possible conflicts of interest** *(for study authors)* | No | p. 7 |
| **Notes:** | | |

Data extraction form:

The Use of Pledget-Reinforced Sutures During Surgical Aortic Valve Replacement: a Systematic Review and Meta-Analysis

Notes on using data extraction form:

Be consistent in the order and style you use to describe the information for each report.

Record any missing information as unclear or not described, to make it clear that the information was not found in the study report(s), not that you forgot to extract it.

Include any instructions and decision rules on the data collection form, or in an accompanying document. It is important to practice using the form and give training to any other authors using the form.

| Review title or ID | Simple Interrupted Suturing for Aortic Valve Replacement in Patients with Severe Aortic Stenosis |
| --- | --- |
| Study ID *(surname of first author and year first full report of study was published e.g. Smith 2001)* | Tabata et al. (2014) |
| Report ID |  |
| Report ID of other reports of this study |  |
| Notes | |

General Information

| Date form completed *(dd/mm/yyyy)* | 8-9-23 |
| --- | --- |
| Name/ID of person extracting data | Taco Boltje |
| Reference citation | Tabata, M., Shibayama, K., Watanabe, H., Sato, Y., Fukui, T., & Takanashi, S. (2014). Simple interrupted suturing increases valve performance after aortic valve replacement with a small supra-annular bioprosthesis. *The Journal of thoracic and cardiovascular surgery*, *147*(1), 321–325. https://doi.org/10.1016/j.jtcvs.2012.11.020 |
| Study author contact details |  |
| Publication type *(e.g. full report, abstract, letter)* | Full text |
| Notes: | |

Study eligibility

| Study Characteristics | Eligibility criteria | | Eligibility criteria met? | | | Location in text or source *(pg & ¶/fig/table/other)* |
| --- | --- | --- | --- | --- | --- | --- |
|  |  |  | Yes | No | Unclear |  |
| Type of study | Randomised Controlled Trial / Observational study | |  |  |  | Abstract |
| Language | English | |  |  |  | Abstract |
| Participants | Patients undergoing SAVR | |  |  |  | Abstract |
| Types of comparison | Pledget-reinforced suturing technique with suturing technique without pledgets | |  |  |  | p. 2 surgical technique |
| INCLUDE | | EXCLUDE | | | | |
| Reason for exclusion |  | | | | | |
| Notes: | | | | | | |

**DO NOT PROCEED IF STUDY EXCLUDED FROM REVIEW**

Characteristics of included studies

Methods

|  | **Descriptions as stated in report/paper** | | **Location in text or source** *(pg & ¶/fig/table/other)* |
| --- | --- | --- | --- |
| **Aim of study** *(e.g. efficacy, equivalence, pragmatic)* | Inferiority | | P. 321 introduction |
| **Design***(e.g. parallel, crossover, non-RCT)* | Retrospective cohort | | p. 321 patients and study design |
| **Unit of allocation**  *(by individuals, cluster/ groups or body parts)* | By individuals | | p. 321 patients and study design |
| **Start date** | Jun 2008 | | p. 321 patients and study design |
| **End date** | December 2010 | | p. 321 patients and study design |
| **Duration of participation**  *(from recruitment to last follow-up)* | - | |  |
| **Ethical approval needed/ obtained for study** | Yes No Unclear |  | p. 321 patients and study design |
| **Notes:** | | | |

Participants

|  | Description  *Include comparative information for each intervention or comparison group if available* | | Location in text or source *(pg & ¶/fig/table/other)* |
| --- | --- | --- | --- |
| Setting  *(including location and social context)* | Sakakibara heart institute | | p. 321 patients and study design |
| Inclusion criteria | AVR with 19- or 21-mm carpentier edwards perimount magna | | p. 321 patients and study design |
| Exclusion criteria | Aortic root replacement, annular enlargement, low ejection fraction (<40%), LV outflow tract obstruction, mitral valve prosthesis | | p. 321 patients and study design |
| Informed consent obtained | Yes No Unclear | waived | p. 321 patients and study design |
| Total no. randomised  *(or total pop. at start of study for NRCTs)* | 152 | | p. 321 patients and study design |
| Sample size per treatment arm  *(if applicable, no., type, no. people per cluster)* | Simple interrupted sutures (pledget) 102, non-everting mattress suturing 50 | |  |
| Baseline imbalances | Sex, annulus size, prosthesis-annulus size ratio | | Table 1 |
| Withdrawals and exclusions  *(if not provided below by outcome)* | 11 (7.2%) lost to follow-up at 1 year | | p. 322 echocardiographic evaluation |
| Age *(mean)* | Simple interrupted 76.3 +- 6.8     non-everting mattress 77.2+- 5.1 | | Table 1 |
| Sex *(percentage)* | Woman simple interrupted 83 (81.4%) non-everting mattress 29 (58.0%) | | Table 1 |
| Follow-up length | One year | | Table 3 |
| Notes: | | | |

Intervention groups

*Copy and paste table for each intervention and comparison group*

**Intervention Group 1**

|  | Description as stated in report/paper | Location in text or source *(pg & ¶/fig/table/other)* |
| --- | --- | --- |
| Group name | Simple interrupted sutures | Table 1 |
| No. randomised to group  *(specify whether no. people or clusters)* | 102 | Table 1 |
| Type of prosthetic valve implanted (mechanical or biological) | biological | p. 321 introduction |
| Size of prosthetic valve implanted | 19, 21 | p. 321 introduction |
| Confounders adjusted for | sex, body surface area, ejection fraction, annulus size, and implantation of 19-mm prosthesis | p. 322 statistical analysis |
| Adjustment method | Multivariate regression analysis | p. 322 statistical analysis |
| Notes: | | |

**Comparison Group 1**

|  | Description as stated in report/paper | Location in text or source *(pg & ¶/fig/table/other)* |
| --- | --- | --- |
| Group name | Non-everting mattress sutures | Table 1 |
| No. randomised to group  *(specify whether no. people or clusters)* | 50 | Table 1 |
| Type of prosthetic valve implanted (mechanical or biological) | biological | p. 321 introduction |
| Size of prosthetic valve implanted | 19, 21 | p. 321 introduction |
| Confounders adjusted for | sex, body surface area, ejection fraction, annulus size, and implantation of 19-mm prosthesis | p. 322 statistical analysis |
| Adjustment method | Multivariate regression analysis | p. 322 statistical analysis |
| Notes: | | |

Outcomes

*Copy and paste table for each outcome.*

**Primary outcome: parvalvular leak**

|  | Description as stated in report/paper | Location in text or source *(pg & ¶/fig/table/other)* |
| --- | --- | --- |
| Outcome name | Paravalvular leak | Table 3 |
| Time points measured  *(specify whether from start or end of intervention)* | Post-operative, 1 year | Table 3 |
| Time points reported | 2 | Table 3 |
| Outcome definition *(with diagnostic criteria if relevant)* | Paravalvular leak was assessed with TTE and defined as the presence of any regurgitant jet outside the prosthesis frame. | Methods |
| Imputation of missing data *(e.g. assumptions made for ITT analysis)* | N/A |  |
| Notes:      group A, Group B  Paravalvular leak 3 (3.2%) 2 (4.3%) 1.000 | | |

**Secondary outcomes measured post-implantation up to 30 days and during mid-term follow-up**

**Copy table for each outcome present:**

**Thromboembolism, endocarditis, mortality, mean pressure gradient, effective orifice area**

|  | Description as stated in report/paper | Location in text or source *(pg & ¶/fig/table/other)* |
| --- | --- | --- |
| Outcome name | Mean EOA | Table 3 |
| Time points measured  *(specify whether from start or end of intervention)* | Post-operative, 1 year | Table 3 |
| Time points reported | 1 | Table 3 |
| Outcome definition *(with diagnostic criteria if relevant)* | Mean effective orifice area | Table 3 |
| Imputation of missing data *(e.g. assumptions made for ITT analysis)* | N/A |  |
| Notes:      group A, Group B  Mean 1-y EOA (cm2 ) 1.44 +- 0.32 1.36 +- 0.45 .126 | | |

Other

| **Study funding sources** *(including role of funders)* | No funding | p. 6 |
| --- | --- | --- |
| **Possible conflicts of interest** *(for study authors)* | No | p. 7 |
| **Notes:** | | |

Data extraction form:

The Use of Pledget-Reinforced Sutures During Surgical Aortic Valve Replacement: a Systematic Review and Meta-Analysis

Notes on using data extraction form:

Be consistent in the order and style you use to describe the information for each report.

Record any missing information as unclear or not described, to make it clear that the information was not found in the study report(s), not that you forgot to extract it.

Include any instructions and decision rules on the data collection form, or in an accompanying document. It is important to practice using the form and give training to any other authors using the form.

| Review title or ID | **Suture technique does not affect hemodynamic performance of the small supra-annular Trifecta bioprosthesis** |
| --- | --- |
| Study ID *(surname of first author and year first full report of study was published e.g. Smith 2001)* | Ugur et al. (2014) |
| Report ID |  |
| Report ID of other reports of this study |  |
| Notes | |

General Information

| Date form completed *(dd/mm/yyyy)* | 26-09-2023 |
| --- | --- |
| Name/ID of person extracting data | Taco Boltje |
| Reference citation | Ugur, M., Byrne, J. G., Bavaria, J. E., Cheung, A., Petracek, M., Groh, M. A., Suri, R. M., Borger, M. A., & Schaff, H. V. (2014). Suture technique does not affect hemodynamic performance of the small supra-annular Trifecta bioprosthesis. *The Journal of thoracic and cardiovascular surgery*, *148*(4), 1347–1351. https://doi.org/10.1016/j.jtcvs.2013.12.006 |
| Study author contact details |  |
| Publication type *(e.g. full report, abstract, letter)* | Full text |
| Notes: | |

Study eligibility

| Study Characteristics | Eligibility criteria | | Eligibility criteria met? | | | Location in text or source *(pg & ¶/fig/table/other)* |
| --- | --- | --- | --- | --- | --- | --- |
|  |  |  | Yes | No | Unclear |  |
| Type of study | Randomised Controlled Trial / Observational study | |  |  |  | Abstract |
| Language | English | |  |  |  | Abstract |
| Participants | Patients undergoing SAVR | |  |  |  | Abstract |
| Types of comparison | Pledget-reinforced suturing technique with suturing technique without pledgets | |  |  |  | p. 1348 materials and methods |
| INCLUDE | | EXCLUDE | | | | |
| Reason for exclusion |  | | | | | |
| Notes: | | | | | | |

**DO NOT PROCEED IF STUDY EXCLUDED FROM REVIEW**

Characteristics of included studies

Methods

|  | **Descriptions as stated in report/paper** | | **Location in text or source** *(pg & ¶/fig/table/other)* |
| --- | --- | --- | --- |
| **Aim of study** *(e.g. efficacy, equivalence, pragmatic)* | Effectiveness | | p. 1348 materials and methods |
| **Design***(e.g. parallel, crossover, non-RCT)* | Prospective cohort | | p. 1348 materials and methods |
| **Unit of allocation**  *(by individuals, cluster/ groups or body parts)* | By individuals | | p. 1348 materials and methods |
| **Start date** | August 2007 | | Abstract |
| **End date** | November 2009 | | Abstract |
| **Duration of participation**  *(from recruitment to last follow-up)* | 2 year | | abstract |
| **Ethical approval needed/ obtained for study** | Yes No Unclear | Reported in Bavaria JE, Desai ND, Cheung A, Petracek MR, Groh MA, Borger MA, et al.  The St Jude Medical Trifecta aortic pericardial valve: results from a global,  multicenter, prospective clinical study. J Thorac Cardiovasc Surg. 2013  [Epub ahead of print]. |  |
| **Notes:** | | | |

Participants

|  | Description  *Include comparative information for each intervention or comparison group if available* | | Location in text or source *(pg & ¶/fig/table/other)* |
| --- | --- | --- | --- |
| Setting  *(including location and social context)* | Multicenter study of the Trifecta aortic valve bovine bioprosthesis | | p. 1348 materials and methods |
| Inclusion criteria | Patients undergoing surgical AVR using either non-everting pledget-reinforced mattress sutures or everting mattress sutures, with or without pledget | | p. 1348 materials and methods |
| Exclusion criteria |  | |  |
| Informed consent obtained | Yes No Unclear | Reported in Bavaria JE, Desai ND, Cheung A, Petracek MR, Groh MA, Borger MA, et al.  The St Jude Medical Trifecta aortic pericardial valve: results from a global,  multicenter, prospective clinical study. J Thorac Cardiovasc Surg. 2013  [Epub ahead of print]. |  |
| Total no. randomised  *(or total pop. at start of study for NRCTs)* | 346 | | p. 1348 results |
| Sample size per treatment arm  *(if applicable, no., type, no. people per cluster)* | Non-everting pledget (n=269)  Everting pledget-reinforced (n=20)  Simple suture (n=32) | | p. 1348 results, Table 4 |
| Baseline imbalances | Isolated AVR | | Table 1 |
| Withdrawals and exclusions  *(if not provided below by outcome)* | - | |  |
| Age *(mean)* | - | | Table 1 |
| Sex *(percentage)* | - | | Table 1 |
| Follow-up length | 1-year | |  |
| Notes: The sample sizes per arm do not add up, it is unclear from the paper what kind of suturing technique was used in the remaining 15 patients. | | | |

Intervention groups

*Copy and paste table for each intervention and comparison group*

**Intervention Group 1**

|  | Description as stated in report/paper | Location in text or source *(pg & ¶/fig/table/other)* |
| --- | --- | --- |
| Group name | Pledget |  |
| No. randomised to group  *(specify whether no. people or clusters)* | Non-everting pledget (n=269)  Everting pledget-reinforced (n=20)  Total 289 | p. 1348 results, Table 4 |
| Type of prosthetic valve implanted (mechanical or biological) | Biological (Trifecta aortic valve bovine prosthesis) | p. 1348 materials and methods |
| Size of prosthetic valve implanted | 19, 21 | Abstract |
| Confounders adjusted for | None | p. 1348 statistical analyses |
| Adjustment method | None |  |
| Notes: | | |

**Comparison Group 1**

|  | Description as stated in report/paper | Location in text or source *(pg & ¶/fig/table/other)* |
| --- | --- | --- |
| Group name | Non-pledget | Table 1 |
| No. randomised to group  *(specify whether no. people or clusters)* | Simple suture (n=32) | p. 1348 results, Table 4 |
| Type of prosthetic valve implanted (mechanical or biological) | Biological (Trifecta aortic valve bovine prosthesis) | p. 1348 materials and methods |
| Size of prosthetic valve implanted | 19, 21 | Abstract |
| Confounders adjusted for | None | p. 1348 statistical analyses |
| Adjustment method | None | p. 1348 results, Table 4 |
| Notes: | | |

Outcomes

*Copy and paste table for each outcome.*

**Primary outcome: parvalvular leak**

|  | Description as stated in report/paper | Location in text or source *(pg & ¶/fig/table/other)* |
| --- | --- | --- |
| Outcome name | Major paravalvular leak | p. 1351 discussion |
| Time points measured  *(specify whether from start or end of intervention)* | Not reported |  |
| Time points reported | Not reported |  |
| Outcome definition *(with diagnostic criteria if relevant)* | Not reported |  |
| Imputation of missing data *(e.g. assumptions made for ITT analysis)* | - |  |
| Notes:      1 major PVL case in non-everting pledgets group. None in rest of the study. | | |

**Secondary outcomes measured post-implantation up to 30 days and during mid-term follow-up**

**Copy table for each outcome present:**

**Thromboembolism, endocarditis, mortality, mean pressure gradient, effective orifice area**

|  | Description as stated in report/paper | Location in text or source *(pg & ¶/fig/table/other)* |
| --- | --- | --- |
| Outcome name | Mean gradient | Tables 2-4 |
| Time points measured  *(specify whether from start or end of intervention)* | 1-year | Tables 2-4 |
| Time points reported | 1 | Tables 2-4 |
| Outcome definition *(with diagnostic criteria if relevant)* | Mean aortic valve gradients were obtained from the  echocardiographic instrument’s software system using planimetry of the  Doppler spectral envelope | p. 1348 methods |
| Imputation of missing data *(e.g. assumptions made for ITT analysis)* | No |  |
| Notes: | | |

|  | Description as stated in report/paper | Location in text or source *(pg & ¶/fig/table/other)* |
| --- | --- | --- |
| Outcome name | EOA | Tables 2-4 |
| Time points measured  *(specify whether from start or end of intervention)* | 1-year | Tables 2-4 |
| Time points reported | 1 | Tables 2-4 |
| Outcome definition *(with diagnostic criteria if relevant)* | Continuity equation | p. 1348 methods |
| Imputation of missing data *(e.g. assumptions made for ITT analysis)* | No |  |
| Notes: | | |

Other

| **Study funding sources** *(including role of funders)* | The study was supported by St Jude Medical Inc (St Jude Medical Inc, St Paul, Minn). | Online JTCVS |
| --- | --- | --- |
| **Possible conflicts of interest** *(for study authors)* | The authors were investigators in the Food and Drug Administration–mandated clinical trial of the small supra-annular Trifecta (St Jude Medical Inc) aortic valve bovine bioprosthesis. Joseph E. Bavaria reports consulting fees from W. L. Gore and lecture fees from St Jude and Medtronic Vascular. Anson Cheung reports consulting fees from St Jude, Edwards, and Medtronic and equity ownership in Entourage Medicals and Neovasc Inc. Mark A. Groh reports consulting fees from St Jude. Rakesh M. Suri reports consulting fees from Sorin (Percival Trial PI and patent applications), and grant support from Edwards, Sorin, and St Jude. Michael A. Borger reports lecture fees from St Jude, Edwards, and Medtronic. All other authors have nothing to disclose with regard to commercial support. | Online JTCVS |
| **Notes:** | | |

Data extraction form:

The Use of Pledget-Reinforced Sutures During Surgical Aortic Valve Replacement: a Systematic Review and Meta-Analysis

Notes on using data extraction form:

Be consistent in the order and style you use to describe the information for each report.

Record any missing information as unclear or not described, to make it clear that the information was not found in the study report(s), not that you forgot to extract it.

Include any instructions and decision rules on the data collection form, or in an accompanying document. It is important to practice using the form and give training to any other authors using the form.

| Review title or ID | Simple Interrupted Suturing for Aortic Valve Replacement in Patients with Severe Aortic Stenosis |
| --- | --- |
| Study ID *(surname of first author and year first full report of study was published e.g. Smith 2001)* | Velders et al. (2023) |
| Report ID |  |
| Report ID of other reports of this study |  |
| Notes | |

General Information

| Date form completed *(dd/mm/yyyy)* | 8-9-23 |
| --- | --- |
| Name/ID of person extracting data | Taco Boltje |
| Reference citation | Velders, B. J. J., Vriesendorp, M. D., Sabik, J. F., 3rd, Dagenais, F., Labrousse, L., Bapat, V., Aldea, G. S., Anyanwu, A. C., Cai, Y., & Klautz, R. J. M. (2022). Pledgeted versus nonpledgeted sutures in aortic valve replacement: Insights from a prospective multicenter trial. *JTCVS techniques*, *17*, 23–46. https://doi.org/10.1016/j.xjtc.2022.10.016 |
| Study author contact details |  |
| Publication type *(e.g. full report, abstract, letter)* | Full text |
| Notes: | |

Study eligibility

| Study Characteristics | Eligibility criteria | | Eligibility criteria met? | | | Location in text or source *(pg & ¶/fig/table/other)* |
| --- | --- | --- | --- | --- | --- | --- |
|  |  |  | Yes | No | Unclear |  |
| Type of study | Randomised Controlled Trial / Observational study | |  |  |  | Abstract |
| Language | English | |  |  |  | Abstract |
| Participants | Patients undergoing SAVR | |  |  |  | Abstract |
| Types of comparison | Pledget-reinforced suturing technique with suturing technique without pledgets | |  |  |  | Abstract |
| INCLUDE | | EXCLUDE | | | | |
| Reason for exclusion |  | | | | | |
| Notes: | | | | | | |

**DO NOT PROCEED IF STUDY EXCLUDED FROM REVIEW**

Characteristics of included studies

Methods

|  | **Descriptions as stated in report/paper** | | **Location in text or source** *(pg & ¶/fig/table/other)* |
| --- | --- | --- | --- |
| **Aim of study** *(e.g. efficacy, equivalence, pragmatic)* | Equivalence | | P. 24 introduction |
| **Design***(e.g. parallel, crossover, non-RCT)* | Prospective cohort, retrospective analysis | | P. 24 study design |
| **Unit of allocation**  *(by individuals, cluster/ groups or body parts)* | By individuals | | p. 24 study deisgn |
| **Start date** | N/A | |  |
| **End date** | N/A | |  |
| **Duration of participation**  *(from recruitment to last follow-up)* | 5 year | | p. 24 follow-up and end points |
| **Ethical approval needed/ obtained for study** | Yes No Unclear |  | p. study deisgn |
| **Notes:** | | | |

Participants

|  | Description  *Include comparative information for each intervention or comparison group if available* | | Location in text or source *(pg & ¶/fig/table/other)* |
| --- | --- | --- | --- |
| Setting  *(including location and social context)* | Multi-center study with sites in US, Canada and Europe | | p. 24 study design |
| Inclusion criteria | symptomatic patients with moderate or severe aortic stenosis or chronic, severe aortic regurgitation who were admitted for surgical AVR according to clinical indication. Concomitant procedure like CABG, left atrial appendage ligation, patent foramen ovale closure, ascending aortic aneurysm or dissection not requiring circulatory arrest, and subaortic membrane resection not require myectomy allowed. | | p.24 study design |
| Exclusion criteria | Previous AVR, figure-of-eight sutures, unknown suture status | | p. 24 study design |
| Informed consent obtained | Yes No Unclear |  | p. 24 study design |
| Total no. randomised  *(or total pop. at start of study for NRCTs)* | 1082 | | Table 2 |
| Sample size per treatment arm  *(if applicable, no., type, no. people per cluster)* | Pledget (n=640) no pledgets (n=442) | | Table 2 |
| Baseline imbalances | Age, BSA, BMI, STS risk, hypertensions, left ventricular hypertrophy, AF, isolated or mixed aortic stenosis as the primary indication for ACR, minimally invasive surgical approach, concomitant procedures, implanted valve sizes | | p. 27 results |
| Withdrawals and exclusions  *(if not provided below by outcome)* | N/A | |  |
| Age *(mean)* | Pledget 69.6 +- 8.5    no pledgets 71.0 +- 9.4 | | Table 2 |
| Sex *(percentage)* | Male Pledget 494 (77.2%) no pledgets 323 (73.1%) | | Table 2 |
| Follow-up length | 5 years | | p. 24 study design |
| Notes: | | | |

Intervention groups

*Copy and paste table for each intervention and comparison group*

**Intervention Group 1**

|  | Description as stated in report/paper | Location in text or source *(pg & ¶/fig/table/other)* |
| --- | --- | --- |
| Group name | Pledget PSM | Table 2 |
| No. randomised to group  *(specify whether no. people or clusters)* | 397 | Table 2 |
| Type of prosthetic valve implanted (mechanical or biological) | Biological | p. 23 introduction |
| Size of prosthetic valve implanted | 19, 21, 23, 25, 27, 29 | Table 2 |
| Confounders adjusted for | Age, sex, BSA, STS, NYHA class III/IV, coronary artery disease, COPD, hypertension, previous MI, renal dysfunction/insufficiency, diabetes mellitus, atrial fibrillation, peripheral vascular disease, previous stroke/ cerebrovascular accident, left ventricular ejection fraction at baseline, mean pressure gradient at baseline, isolated/mixed aortic stenosis, and less invasive approach (hemisternotomy or right anterior thoracotomy). | p. 24 & 27 statistical analysis |
| Adjustment method | Propensity score matching (PSM) | p. 24 statistical analysis |
| Notes: | | |

**Comparison Group 1**

|  | Description as stated in report/paper | Location in text or source *(pg & ¶/fig/table/other)* |
| --- | --- | --- |
| Group name | Non-Pledget PSM | Table 2 |
| No. randomised to group  *(specify whether no. people or clusters)* | 397 | Table 2 |
| Type of prosthetic valve implanted (mechanical or biological) | Biological | p. 23 introduction |
| Size of prosthetic valve implanted | 19, 21, 23, 25, 27, 29 | Table 2 |
| Confounders adjusted for | Age, sex, BSA, STS, NYHA class III/IV, coronary artery disease, COPD, hypertension, previous MI, renal dysfunction/insufficiency, diabetes mellitus, atrial fibrillation, peripheral vascular disease, previous stroke/ cerebrovascular accident, left ventricular ejection fraction at baseline, mean pressure gradient at baseline, isolated/mixed aortic stenosis, and less invasive approach (hemisternotomy or right anterior thoracotomy). | p. 24 & 27 statistical analysis |
| Adjustment method | PSM | p. 24 statistical analysis |
| Notes: | | |

Outcomes

*Copy and paste table for each outcome.*

**Primary outcome: parvalvular leak**

|  | Description as stated in report/paper | Location in text or source *(pg & ¶/fig/table/other)* |
| --- | --- | --- |
| Outcome name | PVL | Table 3 |
| Time points measured  *(specify whether from start or end of intervention)* | 30-day, 1-,2-, and 5 years of follow up | Table 3/4 figure 2 |
| Time points reported | 2 | Figure 3 |
| Outcome definition *(with diagnostic criteria if relevant)* | Not reported | Table 3 |
| Imputation of missing data *(e.g. assumptions made for ITT analysis)* | Missing covariates for PSM  Median imputation | P. 27 statistical analysis |
| Notes: pledgets vs non-pledgets  All PVL 1.1% (0.4%-2.8%) (n = 4) 1.5% (0.5%-4.0%) (n = 4) .96 | | |

**Secondary outcomes measured post-implantation up to 30 days and during mid-term follow-up**

**Copy table for each outcome present:**

**Thromboembolism, endocarditis, mortality, mean pressure gradient, effective orifice area**

|  | Description as stated in report/paper | Location in text or source *(pg & ¶/fig/table/other)* |
| --- | --- | --- |
| Outcome name | Thromboembolism | Table 3 |
| Time points measured  *(specify whether from start or end of intervention)* | 30-day and 5 years of follow up | Figure 1 |
| Time points reported | 2 | Figure 1 |
| Outcome definition *(with diagnostic criteria if relevant)* | Thromboembolism any | Table 3 |
| Imputation of missing data *(e.g. assumptions made for ITT analysis)* | Only before PSM | P. 27 statistical analysis |
| Notes: pledgets vs non-pledgets  Thromboembolism 5.9% (3.9%-8.9%) (n = 22) 6.1% (4.1%-9.3%) (n = 22) .95 | | |

|  | Description as stated in report/paper | Location in text or source *(pg & ¶/fig/table/other)* |
| --- | --- | --- |
| Outcome name | All-cause mortality | Table 3 |
| Time points measured  *(specify whether from start or end of intervention)* | 1, 2, 3, 4 and 5 years of follow up | Table 3 |
| Time points reported | 5 | Table 3 |
| Outcome definition *(with diagnostic criteria if relevant)* | All-cause mortality | Table 3 |
| Imputation of missing data *(e.g. assumptions made for ITT analysis)* | Only before PSM | P. 27 statistical analysis |
| Notes: pledgets vs non-pledgets  All-cause mortality 13.3% (10.0%-17.6%) (n = 45) 10.5% (7.7%-14.2%) (n = 37) .30 | | |

|  | Description as stated in report/paper | Location in text or source *(pg & ¶/fig/table/other)* |
| --- | --- | --- |
| Outcome name | Mean pressure gradient | Table 3 |
| Time points measured  *(specify whether from start or end of intervention)* | 30 days, 5 years of follow up | Table 4 |
| Time points reported | 2 | Table 3 |
| Outcome definition *(with diagnostic criteria if relevant)* | Mean pressure gradient in mmHg | Table 3 |
| Imputation of missing data *(e.g. assumptions made for ITT analysis)* | Only before PSM | P. 27 statistical analysis |
| Notes: pledgets vs non-pledgets  Mean pressure gradient 12.3 +- 4.4 12.3 +- 4.0 .93 | | |

|  | Description as stated in report/paper | Location in text or source *(pg & ¶/fig/table/other)* |
| --- | --- | --- |
| Outcome name | EOA | Table 3 |
| Time points measured  *(specify whether from start or end of intervention)* | 30-days, 5 years of follow up | Table 4 |
| Time points reported | 2 | Table 3 |
| Outcome definition *(with diagnostic criteria if relevant)* | Effective orifice area in cm2 | Table 3 |
| Imputation of missing data *(e.g. assumptions made for ITT analysis)* | Only before PSM | P. 27 statistical analysis |
| Notes: pledgets vs non-pledgets  EOA 1.35 (0.72-2.87) 1.44 (0.79-2.58) .045 | | |

|  | Description as stated in report/paper | Location in text or source *(pg & ¶/fig/table/other)* |
| --- | --- | --- |
| Outcome name | Endocarditis | Figure 2 |
| Time points measured  *(specify whether from start or end of intervention)* | 1, 2, 3, 4 and 5 years follow up | Figure 2 |
| Time points reported | 5 | Figure 2 |
| Outcome definition *(with diagnostic criteria if relevant)* | Endocarditis | Figure 2 |
| Imputation of missing data *(e.g. assumptions made for ITT analysis)* | Only before PSM | P. 27 statistical analysis |
| Notes: pledgets vs non-pledgets | | |

Other

| **Study funding sources** *(including role of funders)* | Medtronic | p. 33 |
| --- | --- | --- |
| **Possible conflicts of interest** *(for study authors)* | Bart J. J. Velders: institutional research grant and speaker fees paid to his department by Medtronic. Michiel D. Vriesendorp: institutional research grant and reimbursement of travel expenses from Medtronic. Joseph F. Sabik III: North American Principal Investigator of the PERIGON Pivotal Trial for Medtronic. Francois Dagenais: speaker and consultant for Medtronic, COOK Medical, and Edwards Lifesciences. Louis Labrousse: research grant from Medtronic, Edwards Lifesciences, and Abbott. Vinayak Bapat: consultant for Medtronic, Edwards Lifesciences, and Abbott. Yaping Cai: employee of Medtronic. Robert J. M. Klautz: research support, consultation fees, and European Principal Investigator of the PERIGON Pivotal Trial for Medtronic. All other authors reported no conflicts of interest. | p. 7 |
| **Notes:** | | |

Data extraction form:

The Use of Pledget-Reinforced Sutures During Surgical Aortic Valve Replacement: a Systematic Review and Meta-Analysis

Notes on using data extraction form:

Be consistent in the order and style you use to describe the information for each report.

Record any missing information as unclear or not described, to make it clear that the information was not found in the study report(s), not that you forgot to extract it.

Include any instructions and decision rules on the data collection form, or in an accompanying document. It is important to practice using the form and give training to any other authors using the form.

| Review title or ID | Effect of valve suture technique on incidence of paraprosthetic regurgitation and 10-year survival. |
| --- | --- |
| Study ID *(surname of first author and year first full report of study was published e.g. Smith 2001)* | Nair et al. (2010) |
| Report ID |  |
| Report ID of other reports of this study |  |
| Notes | |

General Information

| Date form completed *(dd/mm/yyyy)* | 02/05/2024 |
| --- | --- |
| Name/ID of person extracting data | M. Carvalho Mota |
| Reference citation | Nair et al. Effect of valve suture technique on incidence of paraprosthetic regurgitation and 10-year survival. Ann Thorac Surg. 2010 Apr;89(4):1171-9 |
| Study author contact details |  |
| Publication type *(e.g. full report, abstract, letter)* | Full text |
| Notes: | |

Study eligibility

| Study Characteristics | Eligibility criteria | | Eligibility criteria met? | | | Location in text or source *(pg & ¶/fig/table/other)* |
| --- | --- | --- | --- | --- | --- | --- |
|  |  |  | Yes | No | Unclear |  |
| Type of study | Randomised Controlled Trial / Observational study | |  |  |  | Abstract |
| Language | English | |  |  |  |  |
| Participants | Patients undergoing SAVR | |  |  |  |  |
| Types of comparison | Pledget-reinforced suturing technique with suturing technique without pledgets | |  |  |  |  |
| INCLUDE | | EXCLUDE | | | | |
| Reason for exclusion |  | | | | | |
| Notes: | | | | | | |

**DO NOT PROCEED IF STUDY EXCLUDED FROM REVIEW**

Characteristics of included studies

Methods

|  | **Descriptions as stated in report/paper** | | **Location in text or source** *(pg & ¶/fig/table/other)* |
| --- | --- | --- | --- |
| **Aim of study** *(e.g. efficacy, equivalence, pragmatic)* | to estimate the risk of paraprosthetic regurgitation (PPR) after aortic (AVR) and mitral valve replacement (MVR) using inter-  rupted (IN) or semicontinuous (SC) sutures. The second-  ary objective was to estimate the risk of redo valve surgery and 10-year survival after valve replacement performed using either suture technique. | | Abstract, page 1 |
| **Design***(e.g. parallel, crossover, non-RCT)* | RCT inclusion, but PPR data was collected and analyzed retrospectively so therefore retrospective cohort. | | Methods, page 1 |
| **Unit of allocation**  *(by individuals, cluster/ groups or body parts)* | Interrupted - or semicontinuous sutures. | | Abstract, page 1 |
| **Start date** | December 1991 | | Methods, page 1 |
| **End date** | June 1997 | | Methods, page 1 |
| **Duration of participation**  *(from recruitment to last follow-up)* | Median post-operative follow-up after AVR for the PPR detection in the IN group was 206 days (35 to to 1430 days) and for the SC group was 557 days (0 to 1586 days).  Freedom from PPR after AVR was measured 11 years post-op | | Results page 3, 4  Graph page 7 |
| **Ethical approval needed/ obtained for study** | Yes No Unclear | Obtained within the RCT setting |  |
| **Notes:**       This ran-  domized trial was approved by the institutional review  board and the local research ethics committee and has  been published before [8]. Echocardiographic and clini-  cal data of patients who were not part of the randomized  trial were obtained as part of routine clinical follow-up,  and thus separate consent was not sought as individual  patients are not identified. | | | |

Participants

|  | Description  *Include comparative information for each intervention or comparison group if available* | | Location in text or source *(pg & ¶/fig/table/other)* |
| --- | --- | --- | --- |
| Setting  *(including location and social context)* | hospital in the UK, cardiothoracic surgery department | | Page 1 |
| Inclusion criteria | Patients younger than 70 years of age who underwent  AVR or MVR using a St. Jude mechanical prosthesis  between December 1991 and June 1997 were included.  Patients with previous valve operations, treated endo-  carditis, and concomitant coronary artery disease were  included. | | Page 1 and 2, patients and methods |
| Exclusion criteria | Patients who underwent valve replacement for active endocarditis and double valve disease were excluded. Two patients who died during surgery and 4 who died within the first month after surgery and who did not have PPR in their postop-erative echocardiogram were also excluded. One patient in the aortic valve group was lost  to follow-up after the first year and hence excluded from  statistical analysis of long-term performance and survival  but included in assessing the incidence of PPR. | | Page 1 and 2, patients and methods |
| Informed consent obtained | Yes No Unclear | waiver |  |
| Total no. randomised  *(or total pop. at start of study for NRCTs)* | 67 Mitral + 126 aortic | | Page 2, patients and methods |
| Sample size per treatment arm  *(if applicable, no., type, no. people per cluster)* | 18 patients were identified in the mitral IN and 49  in the mitral SC group. Similarly, 43 patients were  identified in the aortic IN and 83 in the aortic SC group. | | Page 2, patients and methods |
| Baseline imbalances | None | | Page 3 table 2 |
| Withdrawals and exclusions  *(if not provided below by outcome)* | Two patients who died during surgery and 4 who died within the first month after surgery and who did not have PPR in their postop-erative echocardiogram were also excluded. One patient in the aortic valve group was lost  to follow-up after the first year and hence excluded from  statistical analysis of long-term performance and survival  but included in assessing the incidence of PPR. | | Page 1 and 2, patients and methods |
| Age *(mean)* | Median age SC: 63, median age IN: 62 | | Table 2, page 3 |
| Sex *(percentage)* | Overall sex male in the AVR group 100 patients were men  (79.4%) and 26 were women (20.6%).  Sex male SC: 78% (65), sex male IN: 81% (35) | | Table 2, page 3 |
| Follow-up length | Median post-operative follow-up after AVR for the PPR detection in the IN group was 206 days (35 to to 1430 days) and for the SC group was 557 days (0 to 1586 days).  Freedom from PPR after AVR was measured 11 years post-op | | Results page 3, 4  Graph page 7 |
| Notes: | | | |

Intervention groups

*Copy and paste table for each intervention and comparison group*

**Intervention Group 1**

|  | Description as stated in report/paper | Location in text or source *(pg & ¶/fig/table/other)* |
| --- | --- | --- |
| Group name | AVR SC |  |
| No. randomised to group  *(specify whether no. people or clusters)* | 83 | Table 2, page 3 |
| Type of prosthetic valve implanted (mechanical or biological) | St. Jude mechanical aortic prosthesis | Abstract, page 1 |
| Size of prosthetic valve implanted | See table 2 | Table 2, page 3 |
| Confounders adjusted for | No confounders adjusted for. | Page 5 |
| Adjustment method |  |  |
| Notes: the authors do make a prediction model using cox regression analysis: they discover that annular calcification is a predictor of PPR. | | |

**Comparison group 1**

|  | Description as stated in report/paper | Location in text or source *(pg & ¶/fig/table/other)* |
| --- | --- | --- |
| Group name | AVR IN |  |
| No. randomised to group  *(specify whether no. people or clusters)* | 43 | Table 2, page 3 |
| Type of prosthetic valve implanted (mechanical or biological) | St. Jude mechanical aortic prosthesis | Abstract, page 1 |
| Size of prosthetic valve implanted | See table 2 | Table 2, page 3 |
| Confounders adjusted for | No confounders adjusted for. | Page 5 |
| Adjustment method |  |  |
| Notes:  the authors do make a prediction model using cox regression analysis: they discover that annular calcification is a predictor of PPR. | | |

Outcomes

*Copy and paste table for each outcome.*

**Secondary outcomes measured post-implantation up to 30 days and during mid-term follow-up**

**Copy table for each outcome present:**

**Thromboembolism, endocarditis, mortality, mean pressure gradient, effective orifice area**

|  | Description as stated in report/paper | Location in text or source *(pg & ¶/fig/table/other)* |
| --- | --- | --- |
| Outcome name | PPR | Page 4, bottom right |
| Time points measured  *(specify whether from start or end of intervention)* | 0 to 11 years post-op | Fig 3, page 7 |
| Time points reported | 0 to 11 years post-op | Fig 3, page 7 |
| Outcome definition *(with diagnostic criteria if relevant)* | Patients with PPR observed in the transthoracic echocardiogram were discussed with the cardiologist, taking due consideration of all clinical variables to arrive at a consensus plan of action. In general terms, redo valve operation was performed if the PPR was graded as moderate or severe and conservative management was adopted if it was mild or trivial. | Page 2 , follow up |
| Imputation of missing data *(e.g. assumptions made for ITT analysis)* | - |  |
|  | | |

|  | Description as stated in report/paper | Location in text or source *(pg & ¶/fig/table/other)* |
| --- | --- | --- |
| Outcome name | Mortality |  |
| Time points measured  *(specify whether from start or end of intervention)* | 0 to 11 years post-op | Fig 3, page 8 |
| Time points reported | 0 to 11 years post-op | Fig 3, page 8 |
| Outcome definition *(with diagnostic criteria if relevant)* | Death |  |
| Imputation of missing data *(e.g. assumptions made for ITT analysis)* | None reported |  |
|  | | |

Other

| **Study funding sources** *(including role of funders)* | Financial support for part of the study from St. Jude  Medical UK, Ltd, and Edwards Lifesciences, Ltd,  in the organization of the randomized trial that  later contributed to many patients included in this retrospective  study. | Page 8 |
| --- | --- | --- |
| **Possible conflicts of interest** *(for study authors)* | None reported |  |
| **Notes:** | | |

# **File S3. Risk of bias assessments.**

Risk of bias assessment

Responses underlined in green are potential markers for low risk of bias, and responses in red are potential markers for a risk of bias. Where questions relate only to sign posts to other questions, no formatting is used.

| Review title or ID | Importance of implant technique on risk of major paravalvular leak (PVL) after St. Jude mechanical heart valve replacement: a report from the Artificial Valve Endocarditis Reduction Trial (AVERT) |
| --- | --- |
| Study ID *(surname of first author and year first full report of study was published e.g. Smith 2001)* | Englberger et al. (2005) |
| Report ID |  |
| Report ID of other reports of this study |  |
| Notes | |

|  | **Signalling questions** | **Description** | **Response options** |
| --- | --- | --- | --- |
| **Bias due to confounding** | | | |
|  | 1.1 Is there potential for confounding of the effect of intervention in this study?  **If N/PN to 1.1:** the study can be considered to be at low risk of bias due to confounding and no further signalling questions need be considered | Yes, choice for pledgets was surgeon based. | Y |
|  | **If Y/PY to 1.1**: determine whether there is a need to assess time-varying confounding: |  |  |
|  | 1.2. Was the analysis based on splitting participants’ follow up time according to intervention received?  **If N/PN**, answer questions relating to baseline confounding (1.4 to 1.6)  **If Y/PY**, go to question 1.3. |  | NA / Y / PY / PN / N / NI |
|  | 1.3. Were intervention discontinuations or switches likely to be related to factors that are prognostic for the outcome?  **If N/PN**, answer questions relating to baseline confounding (1.4 to 1.6)  **If Y/PY**, answer questions relating to both baseline and time-varying confounding (1.7 and 1.8) |  | NA / Y / PY / PN / N / NI |

|  | **Questions relating to baseline confounding only** | | |
| --- | --- | --- | --- |
|  | 1.4. Did the authors use an appropriate analysis method that controlled for all the important confounding domains? |  | N |
|  | 1.5. **If Y/PY to 1.4**: Were confounding domains that were controlled for measured validly and reliably by the variables available in this study? |  | NA / Y / PY / PN / N / NI |
|  | 1.6. Did the authors control for any post-intervention variables that could have been affected by the intervention? |  | N |
|  | **Questions relating to baseline and time-varying confounding** | |  |
|  | 1.7. Did the authors use an appropriate analysis method that controlled for all the important confounding domains and for time-varying confounding? |  | NA / Y / PY / PN / N / NI |
|  | 1.8. **If Y/PY to 1.7**: Were confounding domains that were controlled for measured validly and reliably by the variables available in this study? |  | NA / Y / PY / PN / N / NI |
|  | **Risk of bias judgement** | Critical | Critical |
|  | Optional: What is the predicted direction of bias due to confounding? |  | Favours experimental / Favours comparator / Unpredictable |

| **Bias in selection of participants into the study** | | | |
| --- | --- | --- | --- |
|  | 2.1. Was selection of participants into the study (or into the analysis) based on participant characteristics observed after the start of intervention?  **If N/PN to 2.1:** go to 2.4 | The cohort is from an rct | N |
|  | 2.2. **If Y/PY to 2.1**: Were the post-intervention variables that influenced selection likely to be associated with intervention?  2.3 **If Y/PY to 2.2**: Were the post-intervention variables that influenced selection likely to be influenced by the outcome or a cause of the outcome? |  | NA / Y / PY / PN / N / NI  NA / Y / PY / PN / N / NI |
|  | 2.4. Do start of follow-up and start of intervention coincide for most participants? | Yes | Y |
|  | 2.5. **If Y/PY to 2.2 and 2.3, or N/PN to 2.4**: Were adjustment techniques used that are likely to correct for the presence of selection biases? |  | NA / Y / PY / PN / N / NI |
|  | **Risk of bias judgement** | Low | Low |
|  | Optional: What is the predicted direction of bias due to selection of participants into the study? |  | Favours experimental / Favours comparator / Towards null /Away from null / Unpredictable |

| **Bias in classification of interventions** | | | |
| --- | --- | --- | --- |
|  | 3.1 Were intervention groups clearly defined? | Yes in patient population | Y |
|  | 3.2 Was the information used to define intervention groups recorded at the start of the intervention? | Yes | Y |
|  | 3.3 Could classification of intervention status have been affected by knowledge of the outcome or risk of the outcome? | No because its an rct | N |
|  | **Risk of bias judgement** | Low | low |
|  | Optional: What is the predicted direction of bias due to classification of interventions? |  | Favours experimental / Favours comparator / Towards null /Away from null / Unpredictable |

| **Bias due to deviations from intended interventions** | | | |
| --- | --- | --- | --- |
|  | **If your aim for this study is to assess the effect of assignment to intervention, answer questions 4.1 and 4.2** | |  |
|  | 4.1. Were there deviations from the intended intervention beyond what would be expected in usual practice? | There are no deviations | N |
|  | 4.2. **If Y/PY to 4.1**: Were these deviations from intended intervention unbalanced between groups *and* likely to have affected the outcome? |  | NA / Y / PY / PN / N / NI |
|  | **If your aim for this study is to assess the effect of starting and adhering to intervention, answer questions 4.3 to 4.6** | |  |
|  | 4.3. Were important co-interventions balanced across intervention groups? |  | NA / Y / PY / PN / N / NI |
|  | 4.4. Was the intervention implemented successfully for most participants? |  | Y / PY / PN / N / NI |
|  | 4.5. Did study participants adhere to the assigned intervention regimen? |  | Y / PY / PN / N / NI |
|  | 4.6. **If N/PN to 4.3, 4.4 or 4.5**: Was an appropriate analysis used to estimate the effect of starting and adhering to the intervention? |  | NA / Y / PY / PN / N / NI |
|  | **Risk of bias judgement** | Low | Low |
|  | Optional: What is the predicted direction of bias due to deviations from the intended interventions? |  | Favours experimental / Favours comparator / Towards null /Away from null / Unpredictable |

| **Bias due to missing data** | | | |
| --- | --- | --- | --- |
|  | 5.1 Were outcome data available for all, or nearly all, participants? | Yes | Y |
|  | 5.2 Were participants excluded due to missing data on intervention status? | No | N |
|  | 5.3 Were participants excluded due to missing data on other variables needed for the analysis? | No | N |
|  | 5.4 **If PN/N to 5.1, or Y/PY to 5.2 or 5.3**: Are the proportion of participants and reasons for missing data similar across interventions? |  | NA / Y / PY / PN / N / NI |
|  | 5.5 **If PN/N to 5.1, or Y/PY to 5.2 or 5.3**: Is there evidence that results were robust to the presence of missing data? |  | NA / Y / PY / PN / N / NI |
|  | **Risk of bias judgement** | Low | Low |
|  | Optional: What is the predicted direction of bias due to missing data? |  | Favours experimental / Favours comparator / Towards null /Away from null / Unpredictable |

| **Bias in measurement of outcomes** | | | |
| --- | --- | --- | --- |
|  | 6.1 Could the outcome measure have been influenced by knowledge of the intervention received? | Probably no (unlikely that an echocardiographist judged PVL differently when pledgets are used). | PN |
|  | 6.2 Were outcome assessors aware of the intervention received by study participants? | Probably yes | PY |
|  | 6.3 Were the methods of outcome assessment comparable across intervention groups? | Yes, same protocol is used | Y |
|  | 6.4 Were any systematic errors in measurement of the outcome related to intervention received? | No | N |
|  | **Risk of bias judgement** | Moderate | Moderate |
|  | Optional: What is the predicted direction of bias due to measurement of outcomes? |  | Favours experimental / Favours comparator / Towards null /Away from null / Unpredictable |

| **Bias in selection of the reported result** | | | |
| --- | --- | --- | --- |
|  | Is the reported effect estimate likely to be selected, on the basis of the results, from... |  |  |
|  | 7.1. ... multiple outcome *measurements* within the outcome domain? | This is not done | N |
|  | 7.2 ... multiple *analyses* of the intervention-outcome relationship? | This is also not done | N |
|  | 7.3 ... different *subgroups*? | No subgroups reported | N |
|  | **Risk of bias judgement** | Low | Low |
|  | Optional: What is the predicted direction of bias due to selection of the reported result? |  | Favours experimental / Favours comparator / Towards null /Away from null / Unpredictable |

| **Overall bias** | | | |
| --- | --- | --- | --- |
|  | **Risk of bias judgement** | Critical | Critical |
|  | Optional: What is the overall predicted direction of bias for this outcome? |  | Favours experimental / Favours comparator / Towards null /Away from null / Unpredictable |

Risk of bias assessment

Responses underlined in green are potential markers for low risk of bias, and responses in red are potential markers for a risk of bias. Where questions relate only to sign posts to other questions, no formatting is used.

| Review title or ID | Impact of suture techniques for aortic valve replacement on prosthesis-patient mismatch. |
| --- | --- |
| Study ID *(surname of first author and year first full report of study was published e.g. Smith 2001)* | Kim et al. (2020) |
| Report ID |  |
| Report ID of other reports of this study |  |
| Notes | |

|  | **Signalling questions** | **Description** | **Response options** |
| --- | --- | --- | --- |
| **Bias due to confounding** | | | |
|  | 1.1 Is there potential for confounding of the effect of intervention in this study?  **If N/PN to 1.1:** the study can be considered to be at low risk of bias due to confounding and no further signalling questions need be considered | Yes | Y |
|  | **If Y/PY to 1.1**: determine whether there is a need to assess time-varying confounding: |  |  |
|  | 1.2. Was the analysis based on splitting participants’ follow up time according to intervention received?  **If N/PN**, answer questions relating to baseline confounding (1.4 to 1.6)  **If Y/PY**, go to question 1.3. |  | NA / Y / PY / PN / N / NI |
|  | 1.3. Were intervention discontinuations or switches likely to be related to factors that are prognostic for the outcome?  **If N/PN**, answer questions relating to baseline confounding (1.4 to 1.6)  **If Y/PY**, answer questions relating to both baseline and time-varying confounding (1.7 and 1.8) |  | NA / Y / PY / PN / N / NI |

|  | **Questions relating to baseline confounding only** | | |
| --- | --- | --- | --- |
|  | 1.4. Did the authors use an appropriate analysis method that controlled for all the important confounding domains? | No | N |
|  | 1.5. **If Y/PY to 1.4**: Were confounding domains that were controlled for measured validly and reliably by the variables available in this study? |  | NA / Y / PY / PN / N / NI |
|  | 1.6. Did the authors control for any post-intervention variables that could have been affected by the intervention? |  | N |
|  | **Questions relating to baseline and time-varying confounding** | |  |
|  | 1.7. Did the authors use an appropriate analysis method that controlled for all the important confounding domains and for time-varying confounding? |  | NA / Y / PY / PN / N / NI |
|  | 1.8. **If Y/PY to 1.7**: Were confounding domains that were controlled for measured validly and reliably by the variables available in this study? |  | NA / Y / PY / PN / N / NI |
|  | **Risk of bias judgement** | Critical | critical |
|  | Optional: What is the predicted direction of bias due to confounding? |  | Favours experimental / Favours comparator / Unpredictable |

| **Bias in selection of participants into the study** | | | |
| --- | --- | --- | --- |
|  | 2.1. Was selection of participants into the study (or into the analysis) based on participant characteristics observed after the start of intervention?  **If N/PN to 2.1:** go to 2.4 | No patients were selected on basis of their intervention | N |
|  | 2.2. **If Y/PY to 2.1**: Were the post-intervention variables that influenced selection likely to be associated with intervention?  2.3 **If Y/PY to 2.2**: Were the post-intervention variables that influenced selection likely to be influenced by the outcome or a cause of the outcome? |  | NA / Y / PY / PN / N / NI  NA / Y / PY / PN / N / NI |
|  | 2.4. Do start of follow-up and start of intervention coincide for most participants? | Yes | Y |
|  | 2.5. **If Y/PY to 2.2 and 2.3, or N/PN to 2.4**: Were adjustment techniques used that are likely to correct for the presence of selection biases? |  | NA / Y / PY / PN / N / NI |
|  | **Risk of bias judgement** | Low | Low |
|  | Optional: What is the predicted direction of bias due to selection of participants into the study? |  | Favours experimental / Favours comparator / Towards null /Away from null / Unpredictable |

| **Bias in classification of interventions** | | | |
| --- | --- | --- | --- |
|  | 3.1 Were intervention groups clearly defined? | Yes in surgical technique | Y |
|  | 3.2 Was the information used to define intervention groups recorded at the start of the intervention? | Yes | Y |
|  | 3.3 Could classification of intervention status have been affected by knowledge of the outcome or risk of the outcome? | This is very unlikely, because the intervention is at one time in time and is then not changed | N |
|  | **Risk of bias judgement** | Low | Low |
|  | Optional: What is the predicted direction of bias due to classification of interventions? |  | Favours experimental / Favours comparator / Towards null /Away from null / Unpredictable |

| **Bias due to deviations from intended interventions** | | | |
| --- | --- | --- | --- |
|  | **If your aim for this study is to assess the effect of assignment to intervention, answer questions 4.1 and 4.2** | |  |
|  | 4.1. Were there deviations from the intended intervention beyond what would be expected in usual practice? | No surgical technique is given | N |
|  | 4.2. **If Y/PY to 4.1**: Were these deviations from intended intervention unbalanced between groups *and* likely to have affected the outcome? |  | NA / Y / PY / PN / N / NI |
|  | **If your aim for this study is to assess the effect of starting and adhering to intervention, answer questions 4.3 to 4.6** | |  |
|  | 4.3. Were important co-interventions balanced across intervention groups? |  | NA / Y / PY / PN / N / NI |
|  | 4.4. Was the intervention implemented successfully for most participants? |  | Y / PY / PN / N / NI |
|  | 4.5. Did study participants adhere to the assigned intervention regimen? |  | Y / PY / PN / N / NI |
|  | 4.6. **If N/PN to 4.3, 4.4 or 4.5**: Was an appropriate analysis used to estimate the effect of starting and adhering to the intervention? |  | NA / Y / PY / PN / N / NI |
|  | **Risk of bias judgement** | Low | Low |
|  | Optional: What is the predicted direction of bias due to deviations from the intended interventions? |  | Favours experimental / Favours comparator / Towards null /Away from null / Unpredictable |

| **Bias due to missing data** | | | |
| --- | --- | --- | --- |
|  | 5.1 Were outcome data available for all, or nearly all, participants? | Nearly all, les then 1 procent lost in follow up | Y |
|  | 5.2 Were participants excluded due to missing data on intervention status? | No | N |
|  | 5.3 Were participants excluded due to missing data on other variables needed for the analysis? | No | N |
|  | 5.4 **If PN/N to 5.1, or Y/PY to 5.2 or 5.3**: Are the proportion of participants and reasons for missing data similar across interventions? |  | NA / Y / PY / PN / N / NI |
|  | 5.5 **If PN/N to 5.1, or Y/PY to 5.2 or 5.3**: Is there evidence that results were robust to the presence of missing data? |  | NA / Y / PY / PN / N / NI |
|  | **Risk of bias judgement** | Low | Low |
|  | Optional: What is the predicted direction of bias due to missing data? |  | Favours experimental / Favours comparator / Towards null /Away from null / Unpredictable |

| **Bias in measurement of outcomes** | | | | |
| --- | --- | --- | --- | --- |
|  | 6.1 Could the outcome measure have been influenced by knowledge of the intervention received? | Probably no (unlikely that an echocardiographist judged PVL differently when pledgets are used). | PN | |
|  | 6.2 Were outcome assessors aware of the intervention received by study participants? | Probably yes | PY | |
|  | 6.3 Were the methods of outcome assessment comparable across intervention groups? | Yes, same protocol is used | Y | |
|  | 6.4 Were any systematic errors in measurement of the outcome related to intervention received? | No | N | |
|  | **Risk of bias judgement** | Moderate | Moderate | |
|  | Optional: What is the predicted direction of bias due to measurement of outcomes? |  | Favours experimental / Favours comparator / Towards null /Away from null / Unpredictable | |
| **Bias in selection of the reported result** | | | | |
|  | Is the reported effect estimate likely to be selected, on the basis of the results, from... |  | |  |
|  | 7.1. ... multiple outcome *measurements* within the outcome domain? | No outcome measurement are missed | | N |
|  | 7.2 ... multiple *analyses* of the intervention-outcome relationship? | There are no multiple analyses of the intervention outcome relation ship | | N |
|  | 7.3 ... different *subgroups*? | No because all the subgroups are reported and they are logically reported seperatly | | N |
|  | **Risk of bias judgement** | Low | | Low |
|  | Optional: What is the predicted direction of bias due to selection of the reported result? |  | | Favours experimental / Favours comparator / Towards null /Away from null / Unpredictable |

| **Overall bias** | | | |
| --- | --- | --- | --- |
|  | **Risk of bias judgement** | Critical | Critical |
|  | Optional: What is the overall predicted direction of bias for this outcome? |  | Favours experimental / Favours comparator / Towards null /Away from null / Unpredictable |

Risk of bias assessment

Responses underlined in green are potential markers for low risk of bias, and responses in red are potential markers for a risk of bias. Where questions relate only to sign posts to other questions, no formatting is used.

| Review title or ID | Use of a nonpledgeted suture technique is safe and efficient for aortic valve replacement |
| --- | --- |
| Study ID *(surname of first author and year first full report of study was published e.g. Smith 2001)* | LaPar et al. 2011 |
| Report ID |  |
| Report ID of other reports of this study |  |
| Notes | |

|  | **Signalling questions** | **Description** | **Response options** |
| --- | --- | --- | --- |
| **Bias due to confounding** | | | |

|  | 1.1 Is there potential for confounding of the effect of intervention in this study?  **If N/PN to 1.1:** the study can be considered to be at low risk of bias due to confounding and no further signalling questions need be considered | Yes | Y |
| --- | --- | --- | --- |
|  | **If Y/PY to 1.1**: determine whether there is a need to assess time-varying confounding: |  |  |
|  | 1.2. Was the analysis based on splitting participants’ follow up time according to intervention received?  **If N/PN**, answer questions relating to baseline confounding (1.4 to 1.6)  **If Y/PY**, go to question 1.3. |  | NA / Y / PY / PN / N / NI |
|  | 1.3. Were intervention discontinuations or switches likely to be related to factors that are prognostic for the outcome?  **If N/PN**, answer questions relating to baseline confounding (1.4 to 1.6)  **If Y/PY**, answer questions relating to both baseline and time-varying confounding (1.7 and 1.8) |  | NA / Y / PY / PN / N / NI |

|  | **Questions relating to baseline confounding only** | | |
| --- | --- | --- | --- |
|  | 1.4. Did the authors use an appropriate analysis method that controlled for all the important confounding domains? | No | N |
|  | 1.5. **If Y/PY to 1.4**: Were confounding domains that were controlled for measured validly and reliably by the variables available in this study? |  | NA / Y / PY / PN / N / NI |
|  | 1.6. Did the authors control for any post-intervention variables that could have been affected by the intervention? | No | N |
|  | **Questions relating to baseline and time-varying confounding** | |  |
|  | 1.7. Did the authors use an appropriate analysis method that controlled for all the important confounding domains and for time-varying confounding? |  | NA / Y / PY / PN / N / NI |
|  | 1.8. **If Y/PY to 1.7**: Were confounding domains that were controlled for measured validly and reliably by the variables available in this study? |  | NA / Y / PY / PN / N / NI |
|  | **Risk of bias judgement** | Critical | Critical |
|  | Optional: What is the predicted direction of bias due to confounding? |  | Favours experimental / Favours comparator / Unpredictable |

| **Bias in selection of participants into the study** | | | |
| --- | --- | --- | --- |
|  | 2.1. Was selection of participants into the study (or into the analysis) based on participant characteristics observed after the start of intervention?  **If N/PN to 2.1:** go to 2.4 | No because all isolated AVR operations were retrospectively analysed which is not an patient characteristics which is observed after the start of the intervention. | N |
|  | 2.2. **If Y/PY to 2.1**: Were the post-intervention variables that influenced selection likely to be associated with intervention?  2.3 **If Y/PY to 2.2**: Were the post-intervention variables that influenced selection likely to be influenced by the outcome or a cause of the outcome? |  | NA / Y / PY / PN / N / NI  NA / Y / PY / PN / N / NI |
|  | 2.4. Do start of follow-up and start of intervention coincide for most participants? | Yes, only patients included who complete the follow up | Y |
|  | 2.5. **If Y/PY to 2.2 and 2.3, or N/PN to 2.4**: Were adjustment techniques used that are likely to correct for the presence of selection biases? |  | NA / Y / PY / PN / N / NI |
|  | **Risk of bias judgement** | Low | Low |
|  | Optional: What is the predicted direction of bias due to selection of participants into the study? |  | Favours experimental / Favours comparator / Towards null /Away from null / Unpredictable |

| **Bias in classification of interventions** | | | |
| --- | --- | --- | --- |
|  | 3.1 Were intervention groups clearly defined? | Yes in operative technique | Y |
|  | 3.2 Was the information used to define intervention groups recorded at the start of the intervention? | Yes during the intervention. | Y |
|  | 3.3 Could classification of intervention status have been affected by knowledge of the outcome or risk of the outcome? | No because both interventions heave the risk for the outcomes. | N |
|  | **Risk of bias judgement** | low | low |
|  | Optional: What is the predicted direction of bias due to classification of interventions? |  | Favours experimental / Favours comparator / Towards null /Away from null / Unpredictable |

| **Bias due to deviations from intended interventions** | | | |
| --- | --- | --- | --- |
|  | **If your aim for this study is to assess the effect of assignment to intervention, answer questions 4.1 and 4.2** | |  |
|  | 4.1. Were there deviations from the intended intervention beyond what would be expected in usual practice? | No because the intervention is clear. And the intervention cannot be deviated from. | / N |
|  | 4.2. **If Y/PY to 4.1**: Were these deviations from intended intervention unbalanced between groups *and* likely to have affected the outcome? |  | NA / Y / PY / PN / N / NI |
|  | **If your aim for this study is to assess the effect of starting and adhering to intervention, answer questions 4.3 to 4.6** | |  |
|  | 4.3. Were important co-interventions balanced across intervention groups? |  | NA / Y / PY / PN / N / NI |
|  | 4.4. Was the intervention implemented successfully for most participants? |  | NA / Y / PY / PN / N / NI |
|  | 4.5. Did study participants adhere to the assigned intervention regimen? |  | NA / Y / PY / PN / N / NI |
|  | 4.6. **If N/PN to 4.3, 4.4 or 4.5**: Was an appropriate analysis used to estimate the effect of starting and adhering to the intervention? |  | NA / Y / PY / PN / N / NI |
|  | **Risk of bias judgement** | Low | Low |
|  | Optional: What is the predicted direction of bias due to deviations from the intended interventions? |  | Favours experimental / Favours comparator / Towards null /Away from null / Unpredictable |

| **Bias due to missing data** | | | |
| --- | --- | --- | --- |
|  | 5.1 Were outcome data available for all, or nearly all, participants? | It seems that no patient is lost in follow up. So yes. | Y |
|  | 5.2 Were participants excluded due to missing data on intervention status? | No. | N |
|  | 5.3 Were participants excluded due to missing data on other variables needed for the analysis? | No | N |
|  | 5.4 **If PN/N to 5.1, or Y/PY to 5.2 or 5.3**: Are the proportion of participants and reasons for missing data similar across interventions? |  | NA / Y / PY / PN / N / NI |
|  | 5.5 **If PN/N to 5.1, or Y/PY to 5.2 or 5.3**: Is there evidence that results were robust to the presence of missing data? |  | NA / Y / PY / PN / N / NI |
|  | **Risk of bias judgement** | Low | Low |
|  | Optional: What is the predicted direction of bias due to missing data? |  | Favours experimental / Favours comparator / Towards null /Away from null / Unpredictable |

| **Bias in measurement of outcomes** | | | |
| --- | --- | --- | --- |
|  | 6.1 Could the outcome measure have been influenced by knowledge of the intervention received? | Probably no (unlikely that an echocardiographist judged PVL differently when pledgets are used). | PN |
|  | 6.2 Were outcome assessors aware of the intervention received by study participants? | Probably yes | PY |
|  | 6.3 Were the methods of outcome assessment comparable across intervention groups? | Yes, same protocol is used | Y |
|  | 6.4 Were any systematic errors in measurement of the outcome related to intervention received? | No | N |
|  | **Risk of bias judgement** | Moderate | Moderate |
|  | Optional: What is the predicted direction of bias due to measurement of outcomes? |  | Favours experimental / Favours comparator / Towards null /Away from null / Unpredictable |

| **Bias in selection of the reported result** | | | |
| --- | --- | --- | --- |
|  | Is the reported effect estimate likely to be selected, on the basis of the results, from... | The outcome measurement are clearly stated in the methods section |  |
|  | 7.1. ... multiple outcome *measurements* within the outcome domain? |  | N |
|  | 7.2 ... multiple *analyses* of the intervention-outcome relationship? | Its seems that there is no selectively reporting. Also the statistical analysis clearly states which tests are done. | N |
|  | 7.3 ... different *subgroups*? | There are two different intervention groups created, but these are logical subgroup analyses, so no. | N |
|  | **Risk of bias judgement** | Low | low |
|  | Optional: What is the predicted direction of bias due to selection of the reported result? |  | Favours experimental / Favours comparator / Towards null /Away from null / Unpredictable |

| **Overall bias** | | | |
| --- | --- | --- | --- |
|  | **Risk of bias judgement** | Critical | Critical |
|  | Optional: What is the overall predicted direction of bias for this outcome? |  | Favours experimental / Favours comparator / Towards null /Away from null / Unpredictable |

Risk of bias assessment

Responses underlined in green are potential markers for low risk of bias, and responses in red are potential markers for a risk of bias. Where questions relate only to sign posts to other questions, no formatting is used.

| Review title or ID | Simple Interrupted Suturing for Aortic Valve Replacement in Patients with Severe Aortic Stenosis |
| --- | --- |
| Study ID *(surname of first author and year first full report of study was published e.g. Smith 2001)* | Lee et al. (2020) |
| Report ID |  |
| Report ID of other reports of this study |  |
| Notes | |

|  | **Signalling questions** | **Description** | **Response options** |
| --- | --- | --- | --- |
| **Bias due to confounding** | | | |
|  | 1.1 Is there potential for confounding of the effect of intervention in this study?  **If N/PN to 1.1:** the study can be considered to be at low risk of bias due to confounding and no further signalling questions need be considered | Yes | Y |
|  | **If Y/PY to 1.1**: determine whether there is a need to assess time-varying confounding: |  |  |
|  | 1.2. Was the analysis based on splitting participants’ follow up time according to intervention received?  **If N/PN**, answer questions relating to baseline confounding (1.4 to 1.6)  **If Y/PY**, go to question 1.3. |  | NA / Y / PY / PN / N / NI |
|  | 1.3. Were intervention discontinuations or switches likely to be related to factors that are prognostic for the outcome?  **If N/PN**, answer questions relating to baseline confounding (1.4 to 1.6)  **If Y/PY**, answer questions relating to both baseline and time-varying confounding (1.7 and 1.8) |  | NA / Y / PY / PN / N / NI |

|  | **Questions relating to baseline confounding only** | | |
| --- | --- | --- | --- |
|  | 1.4. Did the authors use an appropriate analysis method that controlled for all the important confounding domains? | No | N |
|  | 1.5. **If Y/PY to 1.4**: Were confounding domains that were controlled for measured validly and reliably by the variables available in this study? |  | NA / Y / PY / PN / N / NI |
|  | 1.6. Did the authors control for any post-intervention variables that could have been affected by the intervention? |  | N |
|  | **Questions relating to baseline and time-varying confounding** | |  |
|  | 1.7. Did the authors use an appropriate analysis method that controlled for all the important confounding domains and for time-varying confounding? |  | NA / Y / PY / PN / N / NI |
|  | 1.8. **If Y/PY to 1.7**: Were confounding domains that were controlled for measured validly and reliably by the variables available in this study? |  | NA / Y / PY / PN / N / NI |
|  | **Risk of bias judgement** | Critical | critical |
|  | Optional: What is the predicted direction of bias due to confounding? |  | Favours experimental / Favours comparator / Unpredictable |

| **Bias in selection of participants into the study** | | | |
| --- | --- | --- | --- |
|  | 2.1. Was selection of participants into the study (or into the analysis) based on participant characteristics observed after the start of intervention?  **If N/PN to 2.1:** go to 2.4 | Patients are not selected because of characteristics observed after the start of the intervention, so no | N |
|  | 2.2. **If Y/PY to 2.1**: Were the post-intervention variables that influenced selection likely to be associated with intervention?  2.3 **If Y/PY to 2.2**: Were the post-intervention variables that influenced selection likely to be influenced by the outcome or a cause of the outcome? |  | NA / Y / PY / PN / N / NI  NA / Y / PY / PN / N / NI |
|  | 2.4. Do start of follow-up and start of intervention coincide for most participants? |  | Y / PY / PN / N / NI |
|  | 2.5. **If Y/PY to 2.2 and 2.3, or N/PN to 2.4**: Were adjustment techniques used that are likely to correct for the presence of selection biases? |  | NA / Y / PY / PN / N / NI |
|  | **Risk of bias judgement** | low | low |
|  | Optional: What is the predicted direction of bias due to selection of participants into the study? |  | Favours experimental / Favours comparator / Towards null /Away from null / Unpredictable |

| **Bias in classification of interventions** | | | |
| --- | --- | --- | --- |
|  | 3.1 Were intervention groups clearly defined? | Yes in surgical procedure | y |
|  | 3.2 Was the information used to define intervention groups recorded at the start of the intervention? | Yes, the technique in the intervention was recorded before. | Y |
|  | 3.3 Could classification of intervention status have been affected by knowledge of the outcome or risk of the outcome? | No | N |
|  | **Risk of bias judgement** | low | low |
|  | Optional: What is the predicted direction of bias due to classification of interventions? |  | Favours experimental / Favours comparator / Towards null /Away from null / Unpredictable |

| **Bias due to deviations from intended interventions** | | | |
| --- | --- | --- | --- |
|  | **If your aim for this study is to assess the effect of assignment to intervention, answer questions 4.1 and 4.2** | |  |
|  | 4.1. Were there deviations from the intended intervention beyond what would be expected in usual practice? | No, either pledgets were used or not. | N |
|  | 4.2. **If Y/PY to 4.1**: Were these deviations from intended intervention unbalanced between groups *and* likely to have affected the outcome? |  | NA / Y / PY / PN / N / NI |
|  | **If your aim for this study is to assess the effect of starting and adhering to intervention, answer questions 4.3 to 4.6** | |  |
|  | 4.3. Were important co-interventions balanced across intervention groups? |  | Y / PY / PN / N / NI |
|  | 4.4. Was the intervention implemented successfully for most participants? |  | Y / PY / PN / N / NI |
|  | 4.5. Did study participants adhere to the assigned intervention regimen? |  | Y / PY / PN / N / NI |
|  | 4.6. **If N/PN to 4.3, 4.4 or 4.5**: Was an appropriate analysis used to estimate the effect of starting and adhering to the intervention? |  | NA / Y / PY / PN / N / NI |
|  | **Risk of bias judgement** | Low | low |
|  | Optional: What is the predicted direction of bias due to deviations from the intended interventions? |  | Favours experimental / Favours comparator / Towards null /Away from null / Unpredictable |

| **Bias due to missing data** | | | |
| --- | --- | --- | --- |
|  | 5.1 Were outcome data available for all, or nearly all, participants? | Yes | Y |
|  | 5.2 Were participants excluded due to missing data on intervention status? | No | N |
|  | 5.3 Were participants excluded due to missing data on other variables needed for the analysis? | No | N |
|  | 5.4 **If PN/N to 5.1, or Y/PY to 5.2 or 5.3**: Are the proportion of participants and reasons for missing data similar across interventions? |  | NA / Y / PY / PN / N / NI |
|  | 5.5 **If PN/N to 5.1, or Y/PY to 5.2 or 5.3**: Is there evidence that results were robust to the presence of missing data? |  | NA / Y / PY / PN / N / NI |
|  | **Risk of bias judgement** | Low | Low |
|  | Optional: What is the predicted direction of bias due to missing data? |  | Favours experimental / Favours comparator / Towards null /Away from null / Unpredictable |

| **Bias in measurement of outcomes** | | | |
| --- | --- | --- | --- |
| 6.1 Could the outcome measure have been influenced by knowledge of the intervention received? | Probably no (unlikely that an echocardiographist judged PVL differently when pledgets are used). | PN |  |
| 6.2 Were outcome assessors aware of the intervention received by study participants? | Probably yes | PY |  |
| 6.3 Were the methods of outcome assessment comparable across intervention groups? | Yes, same protocol is used | Y |  |
| 6.4 Were any systematic errors in measurement of the outcome related to intervention received? | No | N |  |
| **Risk of bias judgement** | Moderate | Moderate |  |
| Optional: What is the predicted direction of bias due to measurement of outcomes? |  | Favours experimental / Favours comparator / Towards null /Away from null / Unpredictable |  |

| **Bias in selection of the reported result** | | | |
| --- | --- | --- | --- |
|  | Is the reported effect estimate likely to be selected, on the basis of the results, from... |  |  |
|  | 7.1. ... multiple outcome *measurements* within the outcome domain? | PN because also other time points were collected | PN |
|  | 7.2 ... multiple *analyses* of the intervention-outcome relationship? | It’s pre specified how every outcome is given so no | N |
|  | 7.3 ... different *subgroups*? | Different subgroups are given but also cohort as a whole and no effect is kept out. | N |
|  | **Risk of bias judgement** | low | low |
|  | Optional: What is the predicted direction of bias due to selection of the reported result? |  | Favours experimental / Favours comparator / Towards null /Away from null / Unpredictable |

| **Overall bias** | | | |
| --- | --- | --- | --- |
|  | **Risk of bias judgement** | Critical | Critical |
|  | Optional: What is the overall predicted direction of bias for this outcome? |  | Favours experimental / Favours comparator / Towards null /Away from null / Unpredictable |

Risk of bias assessment

Responses underlined in green are potential markers for low risk of bias, and responses in red are potential markers for a risk of bias. Where questions relate only to sign posts to other questions, no formatting is used.

| Review title or ID | Figure of eight suture technique in aortic valve replacement decreases prosthesis-patient |
| --- | --- |
| Study ID *(surname of first author and year first full report of study was published e.g. Smith 2001)* | Rasheed et al. (2023) |
| Report ID |  |
| Report ID of other reports of this study |  |
| Notes | |

|  | **Signalling questions** | **Description** | **Response options** |
| --- | --- | --- | --- |
| **Bias due to confounding** | | | |
|  | 1.1 Is there potential for confounding of the effect of intervention in this study?  **If N/PN to 1.1:** the study can be considered to be at low risk of bias due to confounding and no further signalling questions need be considered | Yes | Y |
|  | **If Y/PY to 1.1**: determine whether there is a need to assess time-varying confounding: |  |  |
|  | 1.2. Was the analysis based on splitting participants’ follow up time according to intervention received?  **If N/PN**, answer questions relating to baseline confounding (1.4 to 1.6)  **If Y/PY**, go to question 1.3. |  | NA / Y / PY / PN / N / NI |
|  | 1.3. Were intervention discontinuations or switches likely to be related to factors that are prognostic for the outcome?  **If N/PN**, answer questions relating to baseline confounding (1.4 to 1.6)  **If Y/PY**, answer questions relating to both baseline and time-varying confounding (1.7 and 1.8) |  | NA / Y / PY / PN / N / NI |

|  | **Questions relating to baseline confounding only** | | |
| --- | --- | --- | --- |
|  | 1.4. Did the authors use an appropriate analysis method that controlled for all the important confounding domains? | No | N |
|  | 1.5. **If Y/PY to 1.4**: Were confounding domains that were controlled for measured validly and reliably by the variables available in this study? |  | NA / Y / PY / PN / N / NI |
|  | 1.6. Did the authors control for any post-intervention variables that could have been affected by the intervention? |  | N |
|  | **Questions relating to baseline and time-varying confounding** | |  |
|  | 1.7. Did the authors use an appropriate analysis method that controlled for all the important confounding domains and for time-varying confounding? |  | NA / Y / PY / PN / N / NI |
|  | 1.8. **If Y/PY to 1.7**: Were confounding domains that were controlled for measured validly and reliably by the variables available in this study? |  | NA / Y / PY / PN / N / NI |
|  | **Risk of bias judgement** | Critical | critical |
|  | Optional: What is the predicted direction of bias due to confounding? |  | Favours experimental / Favours comparator / Unpredictable |
|  | **Signalling questions** | **Description** | **Response options** |
| **Bias due to confounding** | | | |
|  | 1.1 Is there potential for confounding of the effect of intervention in this study?  **If N/PN to 1.1:** the study can be considered to be at low risk of bias due to confounding and no further signalling questions need be considered | Yes | Y |
|  | **If Y/PY to 1.1**: determine whether there is a need to assess time-varying confounding: |  |  |
|  | 1.2. Was the analysis based on splitting participants’ follow up time according to intervention received?  **If N/PN**, answer questions relating to baseline confounding (1.4 to 1.6)  **If Y/PY**, go to question 1.3. |  | NA / Y / PY / PN / N / NI |
|  | 1.3. Were intervention discontinuations or switches likely to be related to factors that are prognostic for the outcome?  **If N/PN**, answer questions relating to baseline confounding (1.4 to 1.6)  **If Y/PY**, answer questions relating to both baseline and time-varying confounding (1.7 and 1.8) |  | NA / Y / PY / PN / N / NI |

|  | **Questions relating to baseline confounding only** | | |
| --- | --- | --- | --- |
|  | 1.4. Did the authors use an appropriate analysis method that controlled for all the important confounding domains? | No | N |
|  | 1.5. **If Y/PY to 1.4**: Were confounding domains that were controlled for measured validly and reliably by the variables available in this study? |  | NA / Y / PY / PN / N / NI |
|  | 1.6. Did the authors control for any post-intervention variables that could have been affected by the intervention? |  | N |
|  | **Questions relating to baseline and time-varying confounding** | |  |
|  | 1.7. Did the authors use an appropriate analysis method that controlled for all the important confounding domains and for time-varying confounding? |  | NA / Y / PY / PN / N / NI |
|  | 1.8. **If Y/PY to 1.7**: Were confounding domains that were controlled for measured validly and reliably by the variables available in this study? |  | NA / Y / PY / PN / N / NI |
|  | **Risk of bias judgement** | Critical | critical |
|  | Optional: What is the predicted direction of bias due to confounding? |  | Favours experimental / Favours comparator / Unpredictable |

| **Bias in selection of participants into the study** | | | |
| --- | --- | --- | --- |
|  | 2.1. Was selection of participants into the study (or into the analysis) based on participant characteristics observed after the start of intervention?  **If N/PN to 2.1:** go to 2.4 | Every patient receiving surgical AVR receiving either pledgeted or figure-of-eight suture technique was included | N |
|  | 2.2. **If Y/PY to 2.1**: Were the post-intervention variables that influenced selection likely to be associated with intervention?  2.3 **If Y/PY to 2.2**: Were the post-intervention variables that influenced selection likely to be influenced by the outcome or a cause of the outcome? |  | NA / Y / PY / PN / N / NI  NA / Y / PY / PN / N / NI |
|  | 2.4. Do start of follow-up and start of intervention coincide for most participants? | Yes, it seems that no patients are lost in follow up and then again added, since there is only one timpoint measured, although unclear when this is. | Y |
|  | 2.5. **If Y/PY to 2.2 and 2.3, or N/PN to 2.4**: Were adjustment techniques used that are likely to correct for the presence of selection biases? |  | NA / Y / PY / PN / N / NI |
|  | **Risk of bias judgement** | low | Low |
|  | Optional: What is the predicted direction of bias due to selection of participants into the study? |  | Favours experimental / Favours comparator / Towards null /Away from null / Unpredictable |

| **Bias in classification of interventions** | | | |
| --- | --- | --- | --- |
|  | 3.1 Were intervention groups clearly defined? | Yes in surgical approach | Y |
|  | 3.2 Was the information used to define intervention groups recorded at the start of the intervention? | Yes is this is provided | Y |
|  | 3.3 Could classification of intervention status have been affected by knowledge of the outcome or risk of the outcome? | No | N |
|  | **Risk of bias judgement** | Low | low |
|  | Optional: What is the predicted direction of bias due to classification of interventions? |  | Favours experimental / Favours comparator / Towards null /Away from null / Unpredictable |

| **Bias due to deviations from intended interventions** | | | |
| --- | --- | --- | --- |
|  | **If your aim for this study is to assess the effect of assignment to intervention, answer questions 4.1 and 4.2** | |  |
|  | 4.1. Were there deviations from the intended intervention beyond what would be expected in usual practice? | This is not the case as the intervention is at one point in time and further treatment is the same across groups | N |
|  | 4.2. **If Y/PY to 4.1**: Were these deviations from intended intervention unbalanced between groups *and* likely to have affected the outcome? |  | NA / Y / PY / PN / N / NI |
|  | **If your aim for this study is to assess the effect of starting and adhering to intervention, answer questions 4.3 to 4.6** | |  |
|  | 4.3. Were important co-interventions balanced across intervention groups? |  | Y / PY / PN / N / NI |
|  | 4.4. Was the intervention implemented successfully for most participants? |  | Y / PY / PN / N / NI |
|  | 4.5. Did study participants adhere to the assigned intervention regimen? |  | Y / PY / PN / N / NI |
|  | 4.6. **If N/PN to 4.3, 4.4 or 4.5**: Was an appropriate analysis used to estimate the effect of starting and adhering to the intervention? |  | NA / Y / PY / PN / N / NI |
|  | **Risk of bias judgement** | low | low |
|  | Optional: What is the predicted direction of bias due to deviations from the intended interventions? |  | Favours experimental / Favours comparator / Towards null /Away from null / Unpredictable |

| **Bias due to missing data** | | | |
| --- | --- | --- | --- |
|  | 5.1 Were outcome data available for all, or nearly all, participants? | Yes see table 2 | Y |
|  | 5.2 Were participants excluded due to missing data on intervention status? | No | N |
|  | 5.3 Were participants excluded due to missing data on other variables needed for the analysis? | Yes | Y |
|  | 5.4 **If PN/N to 5.1, or Y/PY to 5.2 or 5.3**: Are the proportion of participants and reasons for missing data similar across interventions? |  | NA / Y / PY / PN / N / NI |
|  | 5.5 **If PN/N to 5.1, or Y/PY to 5.2 or 5.3**: Is there evidence that results were robust to the presence of missing data? |  | NA / Y / PY / PN / N / NI |
|  | **Risk of bias judgement** | Moderate | Moderate |
|  | Optional: What is the predicted direction of bias due to missing data? |  | Favours experimental / Favours comparator / Towards null /Away from null / Unpredictable |

| **Bias in measurement of outcomes** | | | |
| --- | --- | --- | --- |
| 6.1 Could the outcome measure have been influenced by knowledge of the intervention received? | Probably no (unlikely that an echocardiographist judged PVL differently when pledgets are used). | PN |  |
| 6.2 Were outcome assessors aware of the intervention received by study participants? | Probably yes | PY |  |
| 6.3 Were the methods of outcome assessment comparable across intervention groups? | Yes, same protocol is used | Y |  |
| 6.4 Were any systematic errors in measurement of the outcome related to intervention received? | No | N |  |
| **Risk of bias judgement** | Moderate | Moderate |  |
| Optional: What is the predicted direction of bias due to measurement of outcomes? |  | Favours experimental / Favours comparator / Towards null /Away from null / Unpredictable |  |

| **Bias in selection of the reported result** | | | |
| --- | --- | --- | --- |
|  | Is the reported effect estimate likely to be selected, on the basis of the results, from... |  |  |
|  | 7.1. ... multiple outcome *measurements* within the outcome domain? | The different outcome measurement within the same domain are given. | N |
|  | 7.2 ... multiple *analyses* of the intervention-outcome relationship? | no | N |
|  | 7.3 ... different *subgroups*? | There are different subgroups however these are logical because they could effect the outcome measures | N |
|  | **Risk of bias judgement** | Low | Low |
|  | Optional: What is the predicted direction of bias due to selection of the reported result? |  | Favours experimental / Favours comparator / Towards null /Away from null / Unpredictable |

| **Overall bias** | | | |
| --- | --- | --- | --- |
|  | **Risk of bias judgement** | Critical | Critical |
|  | Optional: What is the overall predicted direction of bias for this outcome? |  | Favours experimental / Favours comparator / Towards null /Away from null / Unpredictable |

Risk of bias assessment

Responses underlined in green are potential markers for low risk of bias, and responses in red are potential markers for a risk of bias. Where questions relate only to sign posts to other questions, no formatting is used.

| Review title or ID | Simple Interrupted Suturing for Aortic Valve Replacement in Patients with Severe Aortic Stenosis |
| --- | --- |
| Study ID *(surname of first author and year first full report of study was published e.g. Smith 2001)* | Tabata et al. (2014) |
| Report ID |  |
| Report ID of other reports of this study |  |
| Notes | |

|  | **Signalling questions** | **Description** | **Response options** |
| --- | --- | --- | --- |
| **Bias due to confounding** | | | |
|  | 1.1 Is there potential for confounding of the effect of intervention in this study?  **If N/PN to 1.1:** the study can be considered to be at low risk of bias due to confounding and no further signalling questions need be considered | PY, some adjustment made however important confounder like age not included | PY |
|  | **If Y/PY to 1.1**: determine whether there is a need to assess time-varying confounding: |  |  |
|  | 1.2. Was the analysis based on splitting participants’ follow up time according to intervention received?  **If N/PN**, answer questions relating to baseline confounding (1.4 to 1.6)  **If Y/PY**, go to question 1.3. |  | NA / Y / PY / PN / N / NI |
|  | 1.3. Were intervention discontinuations or switches likely to be related to factors that are prognostic for the outcome?  **If N/PN**, answer questions relating to baseline confounding (1.4 to 1.6)  **If Y/PY**, answer questions relating to both baseline and time-varying confounding (1.7 and 1.8) |  | NA / Y / PY / PN / N / NI |

|  | **Questions relating to baseline confounding only** | | |
| --- | --- | --- | --- |
|  | 1.4. Did the authors use an appropriate analysis method that controlled for all the important confounding domains? | No | N |
|  | 1.5. **If Y/PY to 1.4**: Were confounding domains that were controlled for measured validly and reliably by the variables available in this study? |  | NA / Y / PY / PN / N / NI |
|  | 1.6. Did the authors control for any post-intervention variables that could have been affected by the intervention? | No | N |
|  | **Questions relating to baseline and time-varying confounding** | |  |
|  | 1.7. Did the authors use an appropriate analysis method that controlled for all the important confounding domains and for time-varying confounding? |  | NA / Y / PY / PN / N / NI |
|  | 1.8. **If Y/PY to 1.7**: Were confounding domains that were controlled for measured validly and reliably by the variables available in this study? |  | NA / Y / PY / PN / N / NI |
|  | **Risk of bias judgement** | serious | Moderate |
|  | Optional: What is the predicted direction of bias due to confounding? |  | Favours experimental / Favours comparator / Unpredictable |

| **Bias in selection of participants into the study** | | | |
| --- | --- | --- | --- |
|  | 2.1. Was selection of participants into the study (or into the analysis) based on participant characteristics observed after the start of intervention?  **If N/PN to 2.1:** go to 2.4 | Patients were included because they needed SAVR for any reason so this is observed before the intervention. | N |
|  | 2.2. **If Y/PY to 2.1**: Were the post-intervention variables that influenced selection likely to be associated with intervention?  2.3 **If Y/PY to 2.2**: Were the post-intervention variables that influenced selection likely to be influenced by the outcome or a cause of the outcome? |  | NA / Y / PY / PN / N / NI  NA / Y / PY / PN / N / NI |
|  | 2.4. Do start of follow-up and start of intervention coincide for most participants? | Almost all particapant | Y |
|  | 2.5. **If Y/PY to 2.2 and 2.3, or N/PN to 2.4**: Were adjustment techniques used that are likely to correct for the presence of selection biases? |  | NA / Y / PY / PN / N / NI |
|  | **Risk of bias judgement** | Low | Low |
|  | Optional: What is the predicted direction of bias due to selection of participants into the study? |  | Favours experimental / Favours comparator / Towards null /Away from null / Unpredictable |

| **Bias in classification of interventions** | | | |
| --- | --- | --- | --- |
|  | 3.1 Were intervention groups clearly defined? | Yes in the surgical procedure is the information given | Y |
|  | 3.2 Was the information used to define intervention groups recorded at the start of the intervention? | Yes same as above | Y |
|  | 3.3 Could classification of intervention status have been affected by knowledge of the outcome or risk of the outcome? | No | N |
|  | **Risk of bias judgement** | Low | Low |
|  | Optional: What is the predicted direction of bias due to classification of interventions? |  | Favours experimental / Favours comparator / Towards null /Away from null / Unpredictable |

| **Bias due to deviations from intended interventions** | | | |
| --- | --- | --- | --- |
|  | **If your aim for this study is to assess the effect of assignment to intervention, answer questions 4.1 and 4.2** | |  |
|  | 4.1. Were there deviations from the intended intervention beyond what would be expected in usual practice? | No deviations | N |
|  | 4.2. **If Y/PY to 4.1**: Were these deviations from intended intervention unbalanced between groups *and* likely to have affected the outcome? |  | NA / Y / PY / PN / N / NI |
|  | **If your aim for this study is to assess the effect of starting and adhering to intervention, answer questions 4.3 to 4.6** | |  |
|  | 4.3. Were important co-interventions balanced across intervention groups? |  | Y / PY / PN / N / NI |
|  | 4.4. Was the intervention implemented successfully for most participants? |  | Y / PY / PN / N / NI |
|  | 4.5. Did study participants adhere to the assigned intervention regimen? |  | Y / PY / PN / N / NI |
|  | 4.6. **If N/PN to 4.3, 4.4 or 4.5**: Was an appropriate analysis used to estimate the effect of starting and adhering to the intervention? |  | NA / Y / PY / PN / N / NI |
|  | **Risk of bias judgement** | Low | Low |
|  | Optional: What is the predicted direction of bias due to deviations from the intended interventions? |  | Favours experimental / Favours comparator / Towards null /Away from null / Unpredictable |

| **Bias due to missing data** | | | |
| --- | --- | --- | --- |
|  | 5.1 Were outcome data available for all, or nearly all, participants? | Only 92.8% had follow up echocardiography | PY |
|  | 5.2 Were participants excluded due to missing data on intervention status? | No | N |
|  | 5.3 Were participants excluded due to missing data on other variables needed for the analysis? | N/A | NI |
|  | 5.4 **If PN/N to 5.1, or Y/PY to 5.2 or 5.3**: Are the proportion of participants and reasons for missing data similar across interventions? |  | NA / Y / PY / PN / N / NI |
|  | 5.5 **If PN/N to 5.1, or Y/PY to 5.2 or 5.3**: Is there evidence that results were robust to the presence of missing data? |  | NA / Y / PY / PN / N / NI |
|  | **Risk of bias judgement** | Moderate | Moderate |
|  | Optional: What is the predicted direction of bias due to missing data? |  | Favours experimental / Favours comparator / Towards null /Away from null / Unpredictable |

| **Bias in measurement of outcomes** | | | |
| --- | --- | --- | --- |
| 6.1 Could the outcome measure have been influenced by knowledge of the intervention received? | Probably no (unlikely that an echocardiographist judged PVL differently when pledgets are used). | PN |  |
| 6.2 Were outcome assessors aware of the intervention received by study participants? | Probably yes | PY |  |
| 6.3 Were the methods of outcome assessment comparable across intervention groups? | Yes, same protocol is used | Y |  |
| 6.4 Were any systematic errors in measurement of the outcome related to intervention received? | No | N |  |
| **Risk of bias judgement** | Moderate | Moderate |  |
| Optional: What is the predicted direction of bias due to measurement of outcomes? |  | Favours experimental / Favours comparator / Towards null /Away from null / Unpredictable |  |

| **Bias in selection of the reported result** | | | |
| --- | --- | --- | --- |
|  | Is the reported effect estimate likely to be selected, on the basis of the results, from... | No because also the outcome is given as a whole |  |
|  | 7.1. ... multiple outcome *measurements* within the outcome domain? |  | N |
|  | 7.2 ... multiple *analyses* of the intervention-outcome relationship? | Methods for statistical analysis are reported | N |
|  | 7.3 ... different *subgroups*? | Both the group as a whole and different subgroups are reported so no | N |
|  | **Risk of bias judgement** | Low | Low |
|  | Optional: What is the predicted direction of bias due to selection of the reported result? |  | Favours experimental / Favours comparator / Towards null /Away from null / Unpredictable |

| **Overall bias** | | | |
| --- | --- | --- | --- |
|  | **Risk of bias judgement** | Serious | Serious |
|  | Optional: What is the overall predicted direction of bias for this outcome? |  | Favours experimental / Favours comparator / Towards null /Away from null / Unpredictable |

Risk of bias assessment

Responses underlined in green are potential markers for low risk of bias, and responses in red are potential markers for a risk of bias. Where questions relate only to sign posts to other questions, no formatting is used.

| Review title or ID | **Suture technique does not affect hemodynamic performance of the small supra-annular Trifecta bioprosthesis** |
| --- | --- |
| Study ID *(surname of first author and year first full report of study was published e.g. Smith 2001)* | Ugur et al. (2014) |
| Report ID |  |
| Report ID of other reports of this study |  |
| Notes | |

|  | **Signalling questions** | **Description** | **Response options** |
| --- | --- | --- | --- |
| **Bias due to confounding** | | | |
|  | 1.1 Is there potential for confounding of the effect of intervention in this study?  **If N/PN to 1.1:** the study can be considered to be at low risk of bias due to confounding and no further signalling questions need be considered |  | Y |
|  | **If Y/PY to 1.1**: determine whether there is a need to assess time-varying confounding: |  |  |
|  | 1.2. Was the analysis based on splitting participants’ follow up time according to intervention received?  **If N/PN**, answer questions relating to baseline confounding (1.4 to 1.6)  **If Y/PY**, go to question 1.3. |  | NA / Y / PY / PN / N / NI |
|  | 1.3. Were intervention discontinuations or switches likely to be related to factors that are prognostic for the outcome?  **If N/PN**, answer questions relating to baseline confounding (1.4 to 1.6)  **If Y/PY**, answer questions relating to both baseline and time-varying confounding (1.7 and 1.8) |  | NA / Y / PY / PN / N / NI |

|  | **Questions relating to baseline confounding only** | | |
| --- | --- | --- | --- |
|  | 1.4. Did the authors use an appropriate analysis method that controlled for all the important confounding domains? | No methods used | N |
|  | 1.5. **If Y/PY to 1.4**: Were confounding domains that were controlled for measured validly and reliably by the variables available in this study? |  | NA / Y / PY / PN / N / NI |
|  | 1.6. Did the authors control for any post-intervention variables that could have been affected by the intervention? |  | N |
|  | **Questions relating to baseline and time-varying confounding** | |  |
|  | 1.7. Did the authors use an appropriate analysis method that controlled for all the important confounding domains and for time-varying confounding? |  | NA / Y / PY / PN / N / NI |
|  | 1.8. **If Y/PY to 1.7**: Were confounding domains that were controlled for measured validly and reliably by the variables available in this study? |  | NA / Y / PY / PN / N / NI |
|  | **Risk of bias judgement** | Critical | Critical |
|  | Optional: What is the predicted direction of bias due to confounding? |  | Favours experimental / Favours comparator / Unpredictable |

| **Bias in selection of participants into the study** | | | |
| --- | --- | --- | --- |
|  | 2.1. Was selection of participants into the study (or into the analysis) based on participant characteristics observed after the start of intervention?  **If N/PN to 2.1:** go to 2.4 | 393 eligible but 346 had 1-year echocardiographic examinations available | Y |
|  | 2.2. **If Y/PY to 2.1**: Were the post-intervention variables that influenced selection likely to be associated with intervention?  2.3 **If Y/PY to 2.2**: Were the post-intervention variables that influenced selection likely to be influenced by the outcome or a cause of the outcome? | Such as mortality | PY  PY |
|  | 2.4. Do start of follow-up and start of intervention coincide for most participants? |  | Y |
|  | 2.5. **If Y/PY to 2.2 and 2.3, or N/PN to 2.4**: Were adjustment techniques used that are likely to correct for the presence of selection biases? |  | N |
|  | **Risk of bias judgement** | Moderate | Moderate |
|  | Optional: What is the predicted direction of bias due to selection of participants into the study? |  | Favours experimental / Favours comparator / Towards null /Away from null / Unpredictable |

| **Bias in classification of interventions** | | | |
| --- | --- | --- | --- |
|  | 3.1 Were intervention groups clearly defined? | Yes | Y |
|  | 3.2 Was the information used to define intervention groups recorded at the start of the intervention? | Yes | Y |
|  | 3.3 Could classification of intervention status have been affected by knowledge of the outcome or risk of the outcome? |  | N |
|  | **Risk of bias judgement** | Low | Low |
|  | Optional: What is the predicted direction of bias due to classification of interventions? |  | Favours experimental / Favours comparator / Towards null /Away from null / Unpredictable |

| **Bias due to deviations from intended interventions** | | | |
| --- | --- | --- | --- |
|  | **If your aim for this study is to assess the effect of assignment to intervention, answer questions 4.1 and 4.2** | |  |
|  | 4.1. Were there deviations from the intended intervention beyond what would be expected in usual practice? | There are no deviations | N |
|  | 4.2. **If Y/PY to 4.1**: Were these deviations from intended intervention unbalanced between groups *and* likely to have affected the outcome? |  | NA / Y / PY / PN / N / NI |
|  | **If your aim for this study is to assess the effect of starting and adhering to intervention, answer questions 4.3 to 4.6** | |  |
|  | 4.3. Were important co-interventions balanced across intervention groups? | No, isolated AVR was different in table 1 | N |
|  | 4.4. Was the intervention implemented successfully for most participants? |  | Y |
|  | 4.5. Did study participants adhere to the assigned intervention regimen? |  | Y |
|  | 4.6. **If N/PN to 4.3, 4.4 or 4.5**: Was an appropriate analysis used to estimate the effect of starting and adhering to the intervention? |  | NA / Y / PY / PN / N / NI |
|  | **Risk of bias judgement** | Moderate | Moderate |
|  | Optional: What is the predicted direction of bias due to deviations from the intended interventions? |  | Favours experimental / Favours comparator / Towards null /Away from null / Unpredictable |

| **Bias due to missing data** | | | |
| --- | --- | --- | --- |
|  | 5.1 Were outcome data available for all, or nearly all, participants? | PY, some excluded due to missing 1-year echo outcomes | PY |
|  | 5.2 Were participants excluded due to missing data on intervention status? | No | N |
|  | 5.3 Were participants excluded due to missing data on other variables needed for the analysis? | No | N |
|  | 5.4 **If PN/N to 5.1, or Y/PY to 5.2 or 5.3**: Are the proportion of participants and reasons for missing data similar across interventions? |  | NA / Y / PY / PN / N / NI |
|  | 5.5 **If PN/N to 5.1, or Y/PY to 5.2 or 5.3**: Is there evidence that results were robust to the presence of missing data? |  | NA / Y / PY / PN / N / NI |
|  | **Risk of bias judgement** | Moderate | Moderate |
|  | Optional: What is the predicted direction of bias due to missing data? |  | Favours experimental / Favours comparator / Towards null /Away from null / Unpredictable |

| **Bias in measurement of outcomes** | | |
| --- | --- | --- |
| 6.1 Could the outcome measure have been influenced by knowledge of the intervention received? | Probably no (unlikely that an echocardiographer judged PVL differently when pledgets are used). | PN |
| 6.2 Were outcome assessors aware of the intervention received by study participants? | Probably yes | PY |
| 6.3 Were the methods of outcome assessment comparable across intervention groups? | Yes, same protocol is used | Y |
| 6.4 Were any systematic errors in measurement of the outcome related to intervention received? | No | N |
| **Risk of bias judgement** | Moderate | Moderate |
| Optional: What is the predicted direction of bias due to measurement of outcomes? |  | Favours experimental / Favours comparator / Towards null /Away from null / Unpredictable |

| **Bias in selection of the reported result** | | | |
| --- | --- | --- | --- |
|  | Is the reported effect estimate likely to be selected, on the basis of the results, from... |  |  |
|  | 7.1. ... multiple outcome *measurements* within the outcome domain? |  | N |
|  | 7.2 ... multiple *analyses* of the intervention-outcome relationship? |  | N |
|  | 7.3 ... different *subgroups*? |  | N |
|  | **Risk of bias judgement** | Low | Low |
|  | Optional: What is the predicted direction of bias due to selection of the reported result? |  | Favours experimental / Favours comparator / Towards null /Away from null / Unpredictable |

| **Overall bias** | | | |
| --- | --- | --- | --- |
|  | **Risk of bias judgement** | Critical | Critical |
|  | Optional: What is the overall predicted direction of bias for this outcome? |  | Favours experimental / Favours comparator / Towards null /Away from null / Unpredictable |

Risk of bias assessment

Responses underlined in green are potential markers for low risk of bias, and responses in red are potential markers for a risk of bias. Where questions relate only to sign posts to other questions, no formatting is used.

| Review title or ID | Simple Interrupted Suturing for Aortic Valve Replacement in Patients with Severe Aortic Stenosis |
| --- | --- |
| Study ID *(surname of first author and year first full report of study was published e.g. Smith 2001)* | Velders et al. (2023) |
| Report ID |  |
| Report ID of other reports of this study |  |
| Notes | |

|  | **Signalling questions** | **Description** | **Response options** |
| --- | --- | --- | --- |
| **Bias due to confounding** | | | |
|  | 1.1 Is there potential for confounding of the effect of intervention in this study?  **If N/PN to 1.1:** the study can be considered to be at low risk of bias due to confounding and no further signalling questions need be considered |  | Y |
|  | **If Y/PY to 1.1**: determine whether there is a need to assess time-varying confounding: |  |  |
|  | 1.2. Was the analysis based on splitting participants’ follow up time according to intervention received?  **If N/PN**, answer questions relating to baseline confounding (1.4 to 1.6)  **If Y/PY**, go to question 1.3. |  | NA / Y / PY / PN / N / NI |
|  | 1.3. Were intervention discontinuations or switches likely to be related to factors that are prognostic for the outcome?  **If N/PN**, answer questions relating to baseline confounding (1.4 to 1.6)  **If Y/PY**, answer questions relating to both baseline and time-varying confounding (1.7 and 1.8) |  | NA / Y / PY / PN / N / NI |

|  | **Questions relating to baseline confounding only** | | |
| --- | --- | --- | --- |
|  | 1.4. Did the authors use an appropriate analysis method that controlled for all the important confounding domains? | Adjustment for many measured confounders via PSM but not for surgeon (experience) | PY |
|  | 1.5. **If Y/PY to 1.4**: Were confounding domains that were controlled for measured validly and reliably by the variables available in this study? |  | NA / Y / PY / PN / N / NI |
|  | 1.6. Did the authors control for any post-intervention variables that could have been affected by the intervention? |  | N |
|  | **Questions relating to baseline and time-varying confounding** | |  |
|  | 1.7. Did the authors use an appropriate analysis method that controlled for all the important confounding domains and for time-varying confounding? |  | NA / Y / PY / PN / N / NI |
|  | 1.8. **If Y/PY to 1.7**: Were confounding domains that were controlled for measured validly and reliably by the variables available in this study? |  | NA / Y / PY / PN / N / NI |
|  | **Risk of bias judgement** | Moderate | Moderate |
|  | Optional: What is the predicted direction of bias due to confounding? |  | Favours experimental / Favours comparator / Unpredictable |

| **Bias in selection of participants into the study** | | | |
| --- | --- | --- | --- |
|  | 2.1. Was selection of participants into the study (or into the analysis) based on participant characteristics observed after the start of intervention?  **If N/PN to 2.1:** go to 2.4 | All patients were selected from a database where patients were included before intervention. | N |
|  | 2.2. **If Y/PY to 2.1**: Were the post-intervention variables that influenced selection likely to be associated with intervention?  2.3 **If Y/PY to 2.2**: Were the post-intervention variables that influenced selection likely to be influenced by the outcome or a cause of the outcome? |  | NA / Y / PY / PN / N / NI  NA / Y / PY / PN / N / NI |
|  | 2.4. Do start of follow-up and start of intervention coincide for most participants? | Patients are lost in follow up, but no missed out on for a particular time | Y |
|  | 2.5. **If Y/PY to 2.2 and 2.3, or N/PN to 2.4**: Were adjustment techniques used that are likely to correct for the presence of selection biases? |  | NA / Y / PY / PN / N / NI |
|  | **Risk of bias judgement** | low | Low |
|  | Optional: What is the predicted direction of bias due to selection of participants into the study? |  | Favours experimental / Favours comparator / Towards null /Away from null / Unpredictable |

| **Bias in classification of interventions** | | | |
| --- | --- | --- | --- |
|  | 3.1 Were intervention groups clearly defined? | Pledgets and no pledgets SAVR | Y |
|  | 3.2 Was the information used to define intervention groups recorded at the start of the intervention? | In study design | Y |
|  | 3.3 Could classification of intervention status have been affected by knowledge of the outcome or risk of the outcome? | No | N |
|  | **Risk of bias judgement** | low | low |
|  | Optional: What is the predicted direction of bias due to classification of interventions? |  | Favours experimental / Favours comparator / Towards null /Away from null / Unpredictable |

| **Bias due to deviations from intended interventions** | | | |
| --- | --- | --- | --- |
|  | **If your aim for this study is to assess the effect of assignment to intervention, answer questions 4.1 and 4.2** | |  |
|  | 4.1. Were there deviations from the intended intervention beyond what would be expected in usual practice? | Either pledgets are used or not, intervention is at one time point | N |
|  | 4.2. **If Y/PY to 4.1**: Were these deviations from intended intervention unbalanced between groups *and* likely to have affected the outcome? |  | NA / Y / PY / PN / N / NI |
|  | **If your aim for this study is to assess the effect of starting and adhering to intervention, answer questions 4.3 to 4.6** | |  |
|  | 4.3. Were important co-interventions balanced across intervention groups? |  | Y / PY / PN / N / NI |
|  | 4.4. Was the intervention implemented successfully for most participants? |  | Y / PY / PN / N / NI |
|  | 4.5. Did study participants adhere to the assigned intervention regimen? |  | Y / PY / PN / N / NI |
|  | 4.6. **If N/PN to 4.3, 4.4 or 4.5**: Was an appropriate analysis used to estimate the effect of starting and adhering to the intervention? |  | NA / Y / PY / PN / N / NI |
|  | **Risk of bias judgement** | LOW | Low |
|  | Optional: What is the predicted direction of bias due to deviations from the intended interventions? |  | Favours experimental / Favours comparator / Towards null /Away from null / Unpredictable |

| **Bias due to missing data** | | | |
| --- | --- | --- | --- |
|  | 5.1 Were outcome data available for all, or nearly all, participants? | All patients incluced in PSM are used in the outcomes | Y |
|  | 5.2 Were participants excluded due to missing data on intervention status? | No | N |
|  | 5.3 Were participants excluded due to missing data on other variables needed for the analysis? | No missing data at baseline was computed to include as many particpants as possible. | PN |
|  | 5.4 **If PN/N to 5.1, or Y/PY to 5.2 or 5.3**: Are the proportion of participants and reasons for missing data similar across interventions? |  | NA / Y / PY / PN / N / NI |
|  | 5.5 **If PN/N to 5.1, or Y/PY to 5.2 or 5.3**: Is there evidence that results were robust to the presence of missing data? |  | NA / Y / PY / PN / N / NI |
|  | **Risk of bias judgement** | Low | Low |
|  | Optional: What is the predicted direction of bias due to missing data? |  | Favours experimental / Favours comparator / Towards null /Away from null / Unpredictable |

| **Bias in measurement of outcomes** | | | |
| --- | --- | --- | --- |
| 6.1 Could the outcome measure have been influenced by knowledge of the intervention received? | Probably no (unlikely that an echocardiographist judged PVL differently when pledgets are used). | PN |  |
| 6.2 Were outcome assessors aware of the intervention received by study participants? | Probably yes | PY |  |
| 6.3 Were the methods of outcome assessment comparable across intervention groups? | Yes, same protocol is used | Y |  |
| 6.4 Were any systematic errors in measurement of the outcome related to intervention received? | No | N |  |
| **Risk of bias judgement** | Moderate | Moderate |  |
| Optional: What is the predicted direction of bias due to measurement of outcomes? |  | Favours experimental / Favours comparator / Towards null /Away from null / Unpredictable |  |

| **Bias in selection of the reported result** | | | |
| --- | --- | --- | --- |
|  | Is the reported effect estimate likely to be selected, on the basis of the results, from... |  |  |
|  | 7.1. ... multiple outcome *measurements* within the outcome domain? | Methods for calculating pressure gradient etc. | N |
|  | 7.2 ... multiple *analyses* of the intervention-outcome relationship? | Not the case | N |
|  | 7.3 ... different *subgroups*? | Subgroup analysis is logical, cohort analysis from larger cohort but selected on basis of clear inclusion criteria | N |
|  | **Risk of bias judgement** | Low | Low |
|  | Optional: What is the predicted direction of bias due to selection of the reported result? |  | Favours experimental / Favours comparator / Towards null /Away from null / Unpredictable |

| **Overall bias** | | | |
| --- | --- | --- | --- |
|  | **Risk of bias judgement** | Moderate | Moderate |
|  | Optional: What is the overall predicted direction of bias for this outcome? |  | Favours experimental / Favours comparator / Towards null /Away from null / Unpredictable |

Risk of bias assessment

Responses underlined in green are potential markers for low risk of bias, and responses in red are potential markers for a risk of bias. Where questions relate only to sign posts to other questions, no formatting is used.

| Review title or ID | Simple Interrupted Suturing for Aortic Valve Replacement in Patients with Severe Aortic Stenosis |
| --- | --- |
| Study ID *(surname of first author and year first full report of study was published e.g. Smith 2001)* | Nair et al. (2010) |
| Report ID |  |
| Report ID of other reports of this study |  |
| Notes | |

## Risk of bias assessment

Responses underlined in green are potential markers for low risk of bias, and responses in red are potential markers for a risk of bias. Where questions relate only to sign posts to other questions, no formatting is used.

|  | **Signalling questions** | **Description** | **Response options** |
| --- | --- | --- | --- |
| **Bias due to confounding** | | | |
|  | 1.1 Is there potential for confounding of the effect of intervention in this study?  **If N/PN to 1.1:** the study can be considered to be at low risk of bias due to confounding and no further signalling questions need be considered | Yes | Y |
|  | **If Y/PY to 1.1**: determine whether there is a need to assess time-varying confounding: |  |  |
|  | 1.2. Was the analysis based on splitting participants’ follow up time according to intervention received?  **If N/PN**, answer questions relating to baseline confounding (1.4 to 1.6)  **If Y/PY**, go to question 1.3. | No | N |
|  | 1.3. Were intervention discontinuations or switches likely to be related to factors that are prognostic for the outcome?  **If N/PN**, answer questions relating to baseline confounding (1.4 to 1.6)  **If Y/PY**, answer questions relating to both baseline and time-varying confounding (1.7 and 1.8) |  | NA / Y / PY / PN / N / NI |

|  | **Questions relating to baseline confounding only** | | |
| --- | --- | --- | --- |
|  | 1.4. Did the authors use an appropriate analysis method that controlled for all the important confounding domains? | No | N |
|  | 1.5. **If Y/PY to 1.4**: Were confounding domains that were controlled for measured validly and reliably by the variables available in this study? |  | NA / Y / PY / PN / N / NI |
|  | 1.6. Did the authors control for any post-intervention variables that could have been affected by the intervention? | No | N |
|  | **Questions relating to baseline and time-varying confounding** | |  |
|  | 1.7. Did the authors use an appropriate analysis method that controlled for all the important confounding domains and for time-varying confounding? |  | NA / Y / PY / PN / N / NI |
|  | 1.8. **If Y/PY to 1.7**: Were confounding domains that were controlled for measured validly and reliably by the variables available in this study? |  | NA / Y / PY / PN / N / NI |
|  | **Risk of bias judgement** | Critical | Critical |
|  | Optional: What is the predicted direction of bias due to confounding? |  | Favours experimental / Favours comparator / Unpredictable |

| **Bias in selection of participants into the study** | | | |
| --- | --- | --- | --- |
|  | 2.1. Was selection of participants into the study (or into the analysis) based on participant characteristics observed after the start of intervention?  **If N/PN to 2.1:** go to 2.4 | No | N |
|  | 2.2. **If Y/PY to 2.1**: Were the post-intervention variables that influenced selection likely to be associated with intervention?  2.3 **If Y/PY to 2.2**: Were the post-intervention variables that influenced selection likely to be influenced by the outcome or a cause of the outcome? |  | NA / Y / PY / PN / N / NI  NA / Y / PY / PN / N / NI |
|  | 2.4. Do start of follow-up and start of intervention coincide for most participants? | Yes | Y |
|  | 2.5. **If Y/PY to 2.2 and 2.3, or N/PN to 2.4**: Were adjustment techniques used that are likely to correct for the presence of selection biases? |  | NA / Y / PY / PN / N / NI |
|  | **Risk of bias judgement** | Low | Low |
|  | Optional: What is the predicted direction of bias due to selection of participants into the study? |  | Favours experimental / Favours comparator / Towards null /Away from null / Unpredictable |

| **Bias in classification of interventions** | | | |
| --- | --- | --- | --- |
|  | 3.1 Were intervention groups clearly defined? | Yes | Y |
|  | 3.2 Was the information used to define intervention groups recorded at the start of the intervention? | Yes | Y |
|  | 3.3 Could classification of intervention status have been affected by knowledge of the outcome or risk of the outcome? | No | N |
|  | **Risk of bias judgement** | Low | Low |
|  | Optional: What is the predicted direction of bias due to classification of interventions? |  | Favours experimental / Favours comparator / Towards null /Away from null / Unpredictable |

| **Bias due to deviations from intended interventions** | | | |
| --- | --- | --- | --- |
|  | **If your aim for this study is to assess the effect of assignment to intervention, answer questions 4.1 and 4.2** | |  |
|  | 4.1. Were there deviations from the intended intervention beyond what would be expected in usual practice? | No | N |
|  | 4.2. **If Y/PY to 4.1**: Were these deviations from intended intervention unbalanced between groups *and* likely to have affected the outcome? |  | NA / Y / PY / PN / N / NI |
|  | **If your aim for this study is to assess the effect of starting and adhering to intervention, answer questions 4.3 to 4.6** | |  |
|  | 4.3. Were important co-interventions balanced across intervention groups? | Yes | Y |
|  | 4.4. Was the intervention implemented successfully for most participants? | Yes | Y |
|  | 4.5. Did study participants adhere to the assigned intervention regimen? | Yes | Y |
|  | 4.6. **If N/PN to 4.3, 4.4 or 4.5**: Was an appropriate analysis used to estimate the effect of starting and adhering to the intervention? |  | NA / Y / PY / PN / N / NI |
|  | **Risk of bias judgement** | Low | Low |
|  | Optional: What is the predicted direction of bias due to deviations from the intended interventions? |  | Favours experimental / Favours comparator / Towards null /Away from null / Unpredictable |

| **Bias due to missing data** | | | |
| --- | --- | --- | --- |
|  | 5.1 Were outcome data available for all, or nearly all, participants? | Yes | Y |
|  | 5.2 Were participants excluded due to missing data on intervention status? | No | N |
|  | 5.3 Were participants excluded due to missing data on other variables needed for the analysis? | Yes | Y |
|  | 5.4 **If PN/N to 5.1, or Y/PY to 5.2 or 5.3**: Are the proportion of participants and reasons for missing data similar across interventions? |  | NA / Y / PY / PN / N / NI |
|  | 5.5 **If PN/N to 5.1, or Y/PY to 5.2 or 5.3**: Is there evidence that results were robust to the presence of missing data? |  | NA / Y / PY / PN / N / NI |
|  | **Risk of bias judgement** | low | Low |
|  | Optional: What is the predicted direction of bias due to missing data? |  | Favours experimental / Favours comparator / Towards null /Away from null / Unpredictable |

| **Bias in measurement of outcomes** | | | |
| --- | --- | --- | --- |
|  | 6.1 Could the outcome measure have been influenced by knowledge of the intervention received? | no | N |
|  | 6.2 Were outcome assessors aware of the intervention received by study participants? | yes | Y |
|  | 6.3 Were the methods of outcome assessment comparable across intervention groups? | yes | Y |
|  | 6.4 Were any systematic errors in measurement of the outcome related to intervention received? | Probably no | PN |
|  | **Risk of bias judgement** | Moderate | Moderate |
|  | Optional: What is the predicted direction of bias due to measurement of outcomes? |  | Favours experimental / Favours comparator / Towards null /Away from null / Unpredictable |

| **Bias in selection of the reported result** | | | |
| --- | --- | --- | --- |
|  | Is the reported effect estimate likely to be selected, on the basis of the results, from... | no |  |
|  | 7.1. ... multiple outcome *measurements* within the outcome domain? |  | N |
|  | 7.2 ... multiple *analyses* of the intervention-outcome relationship? | no | N |
|  | 7.3 ... different *subgroups*? | no | N |
|  | **Risk of bias judgement** | low | Low |
|  | Optional: What is the predicted direction of bias due to selection of the reported result? |  | Favours experimental / Favours comparator / Towards null /Away from null / Unpredictable |

| **Overall bias** | | | |
| --- | --- | --- | --- |
|  | **Risk of bias judgement** | Critical | Critical |
|  | Optional: What is the overall predicted direction of bias for this outcome? |  | Favours experimental / Favours comparator / Towards null /Away from null / Unpredictable |


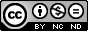


This work is licensed under a [Creative Commons Attribution-NonCommercial-NoDerivatives 4.0 International License](http://creativecommons.org/licenses/by-nc-nd/4.0/)
